# Supplementary figures and images for: Deciphering infected cell types, hub gene networks and cell-cell communication in infectious bronchitis virus via single-cell RNA sequencing
Source: PLoS Pathog. 2024 May 14;20(5):e1012232. doi: 10.1371/journal.ppat.1012232 (PMC11125504; doi:10.1371/journal.ppat.1012232)

DAPI

IBV-N

Merge

Bursa

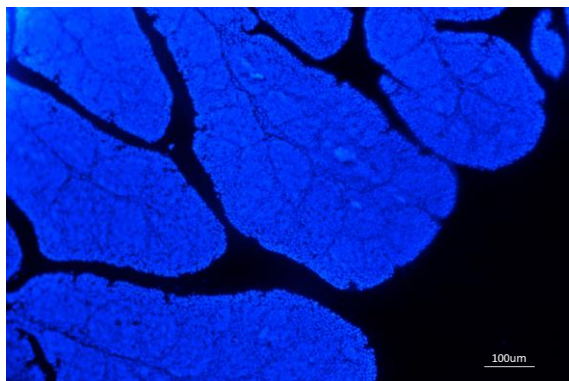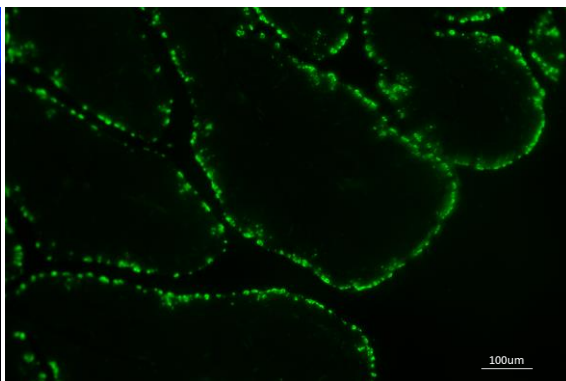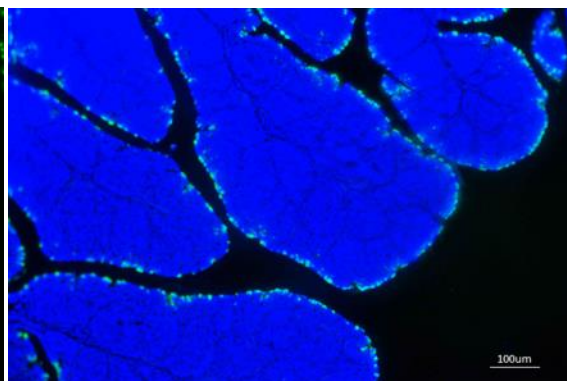

HG

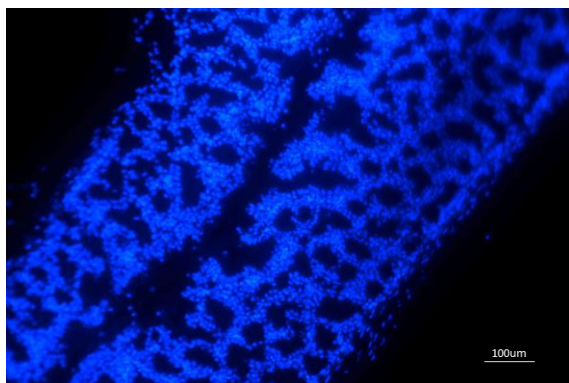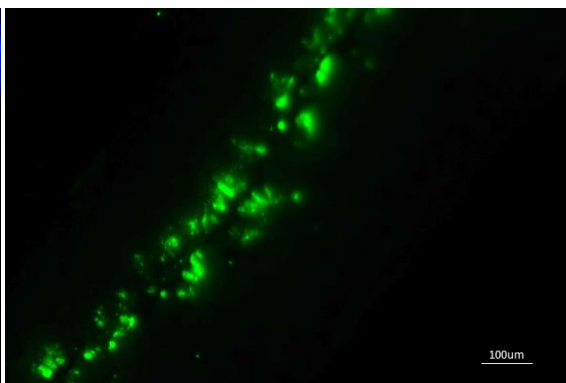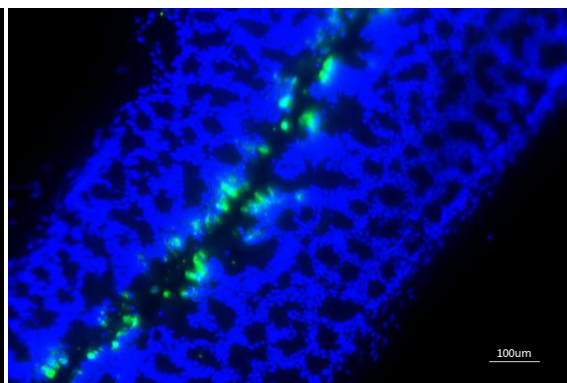

CT

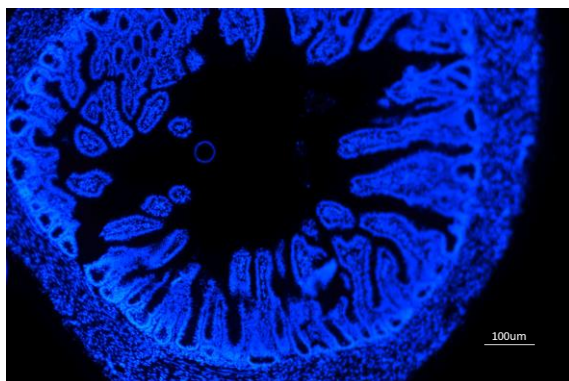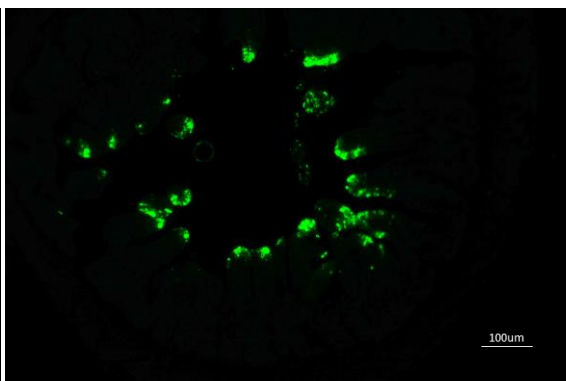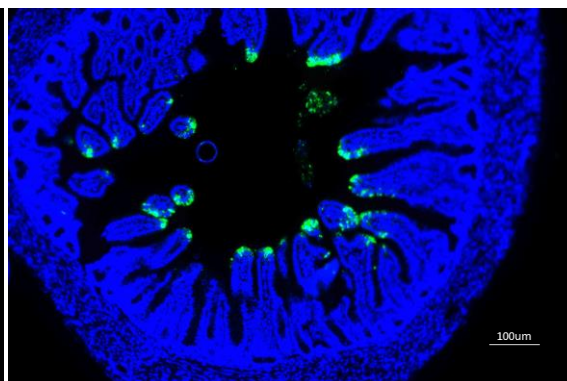

Supplement: S1 Fig — Detection using an antibody against IBV N protein, with virus-positive areas indicated by green fluorescence. HG, Harderian gland; CT, Cecal tonsil. (PDF) [file ppat.1012232.s001.pdf]

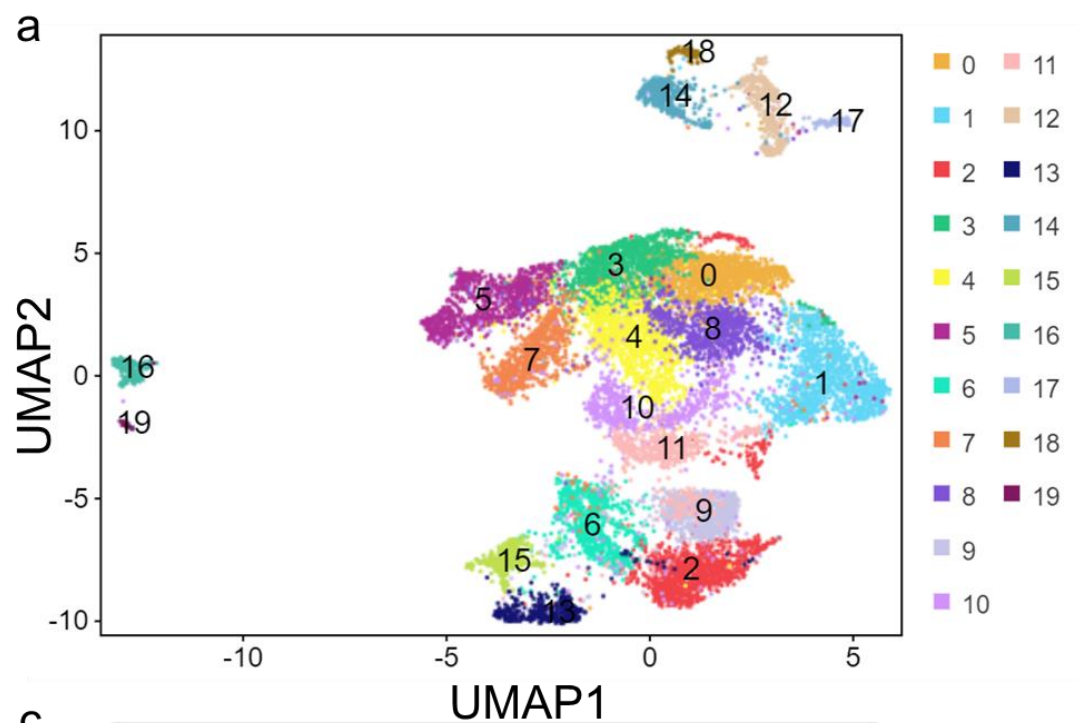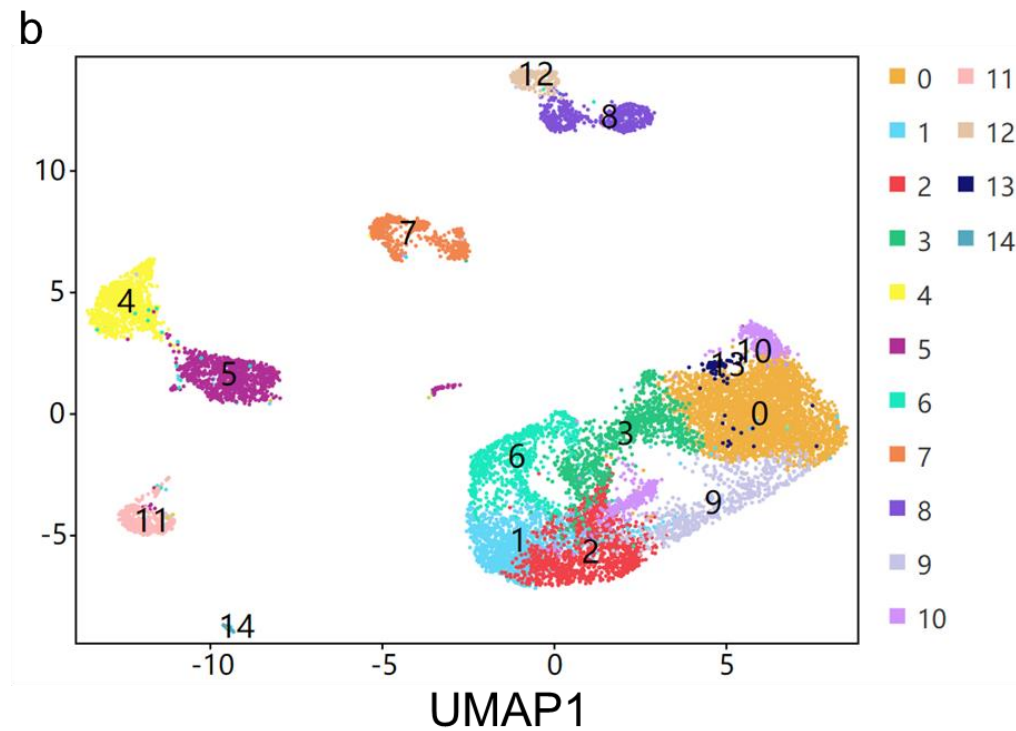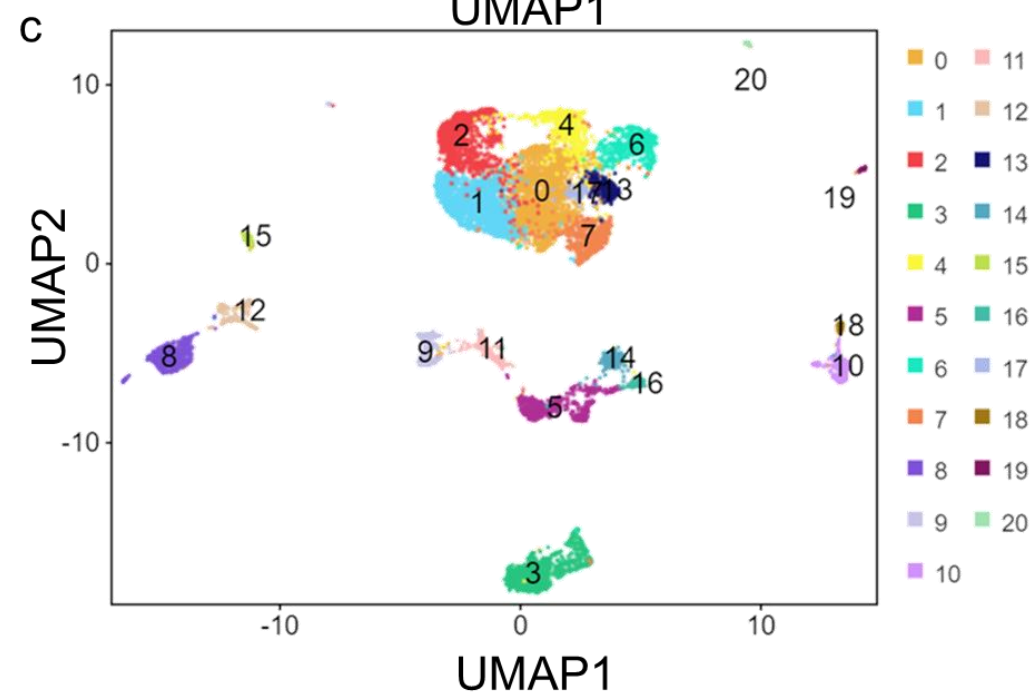

Supplement: S2 Fig — (a) Kidney tissue cell clusters, resolution = 0.85. (b) Bursa tissue cell clusters, resolution = 0.6. (c) Trachea tissue cell clusters, resolution = 0.5. (PDF) [file ppat.1012232.s002.pdf]

a

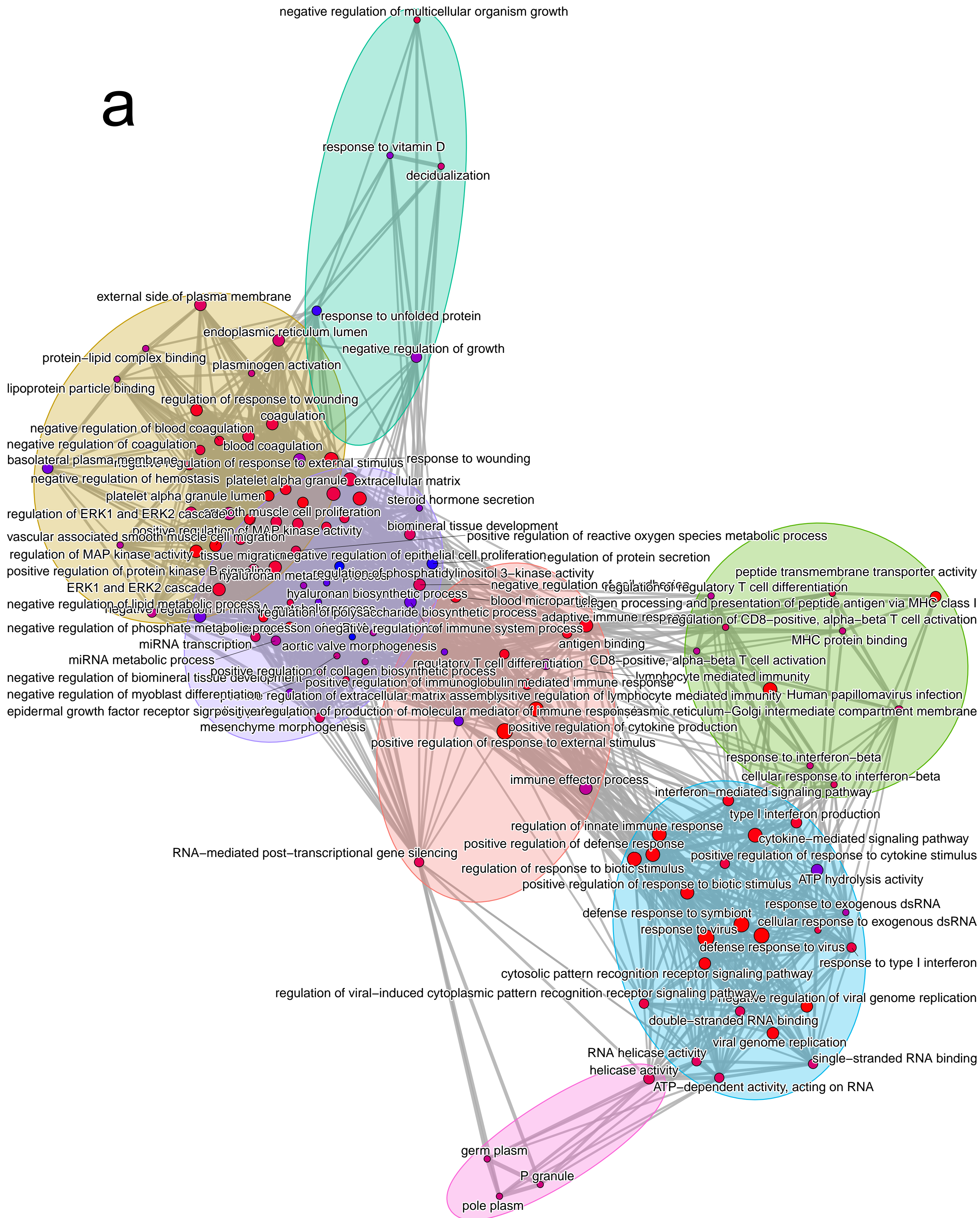

number of genes

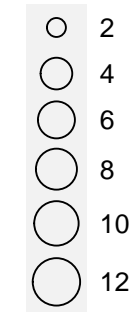

qvalue

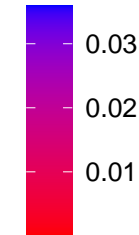

groups

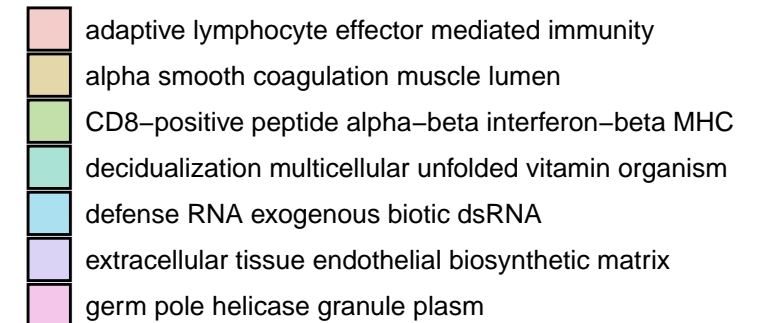

**b**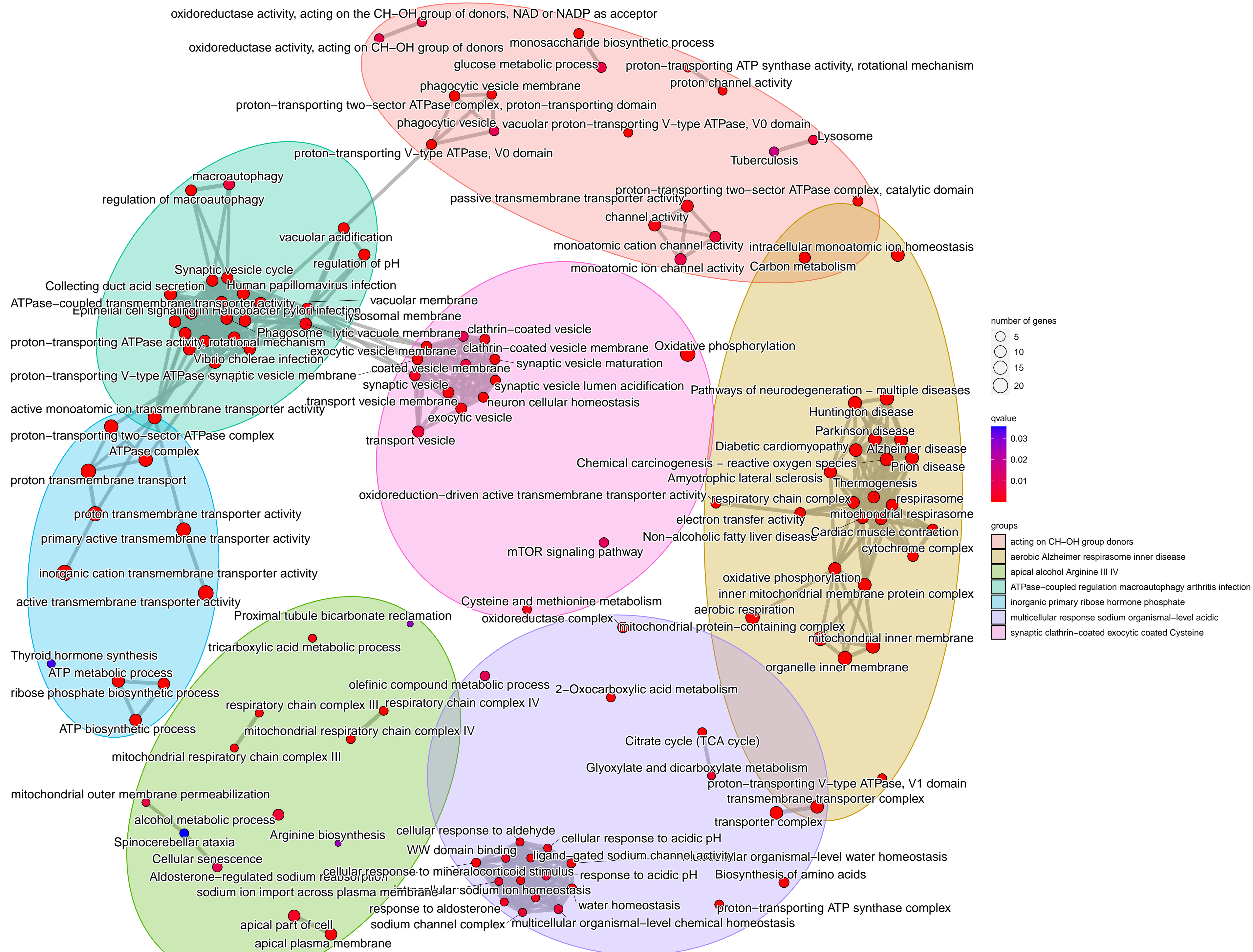

Supplement: S3 Fig — (a) Enrichment of upregulated genes after infection. (b) Enrichment of downregulated genes after infection. log2FC>2, q-value<0.05. (PDF) [file ppat.1012232.s003.pdf]

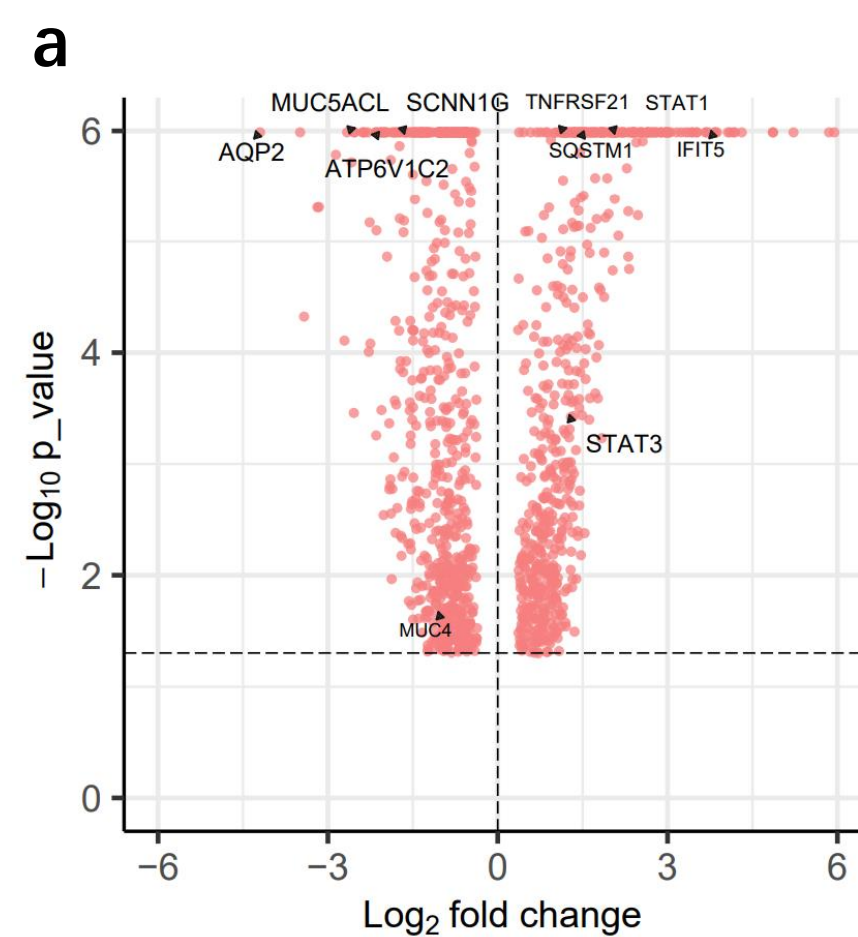

Distal\_collecting tubule

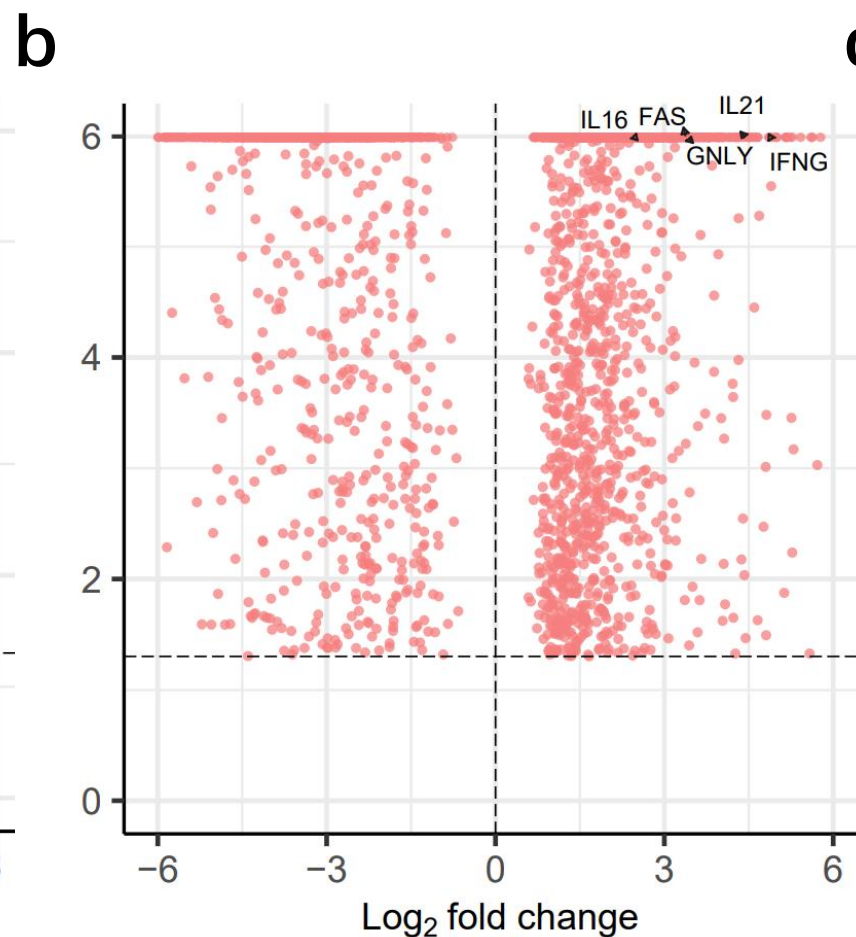

CD8 T cell

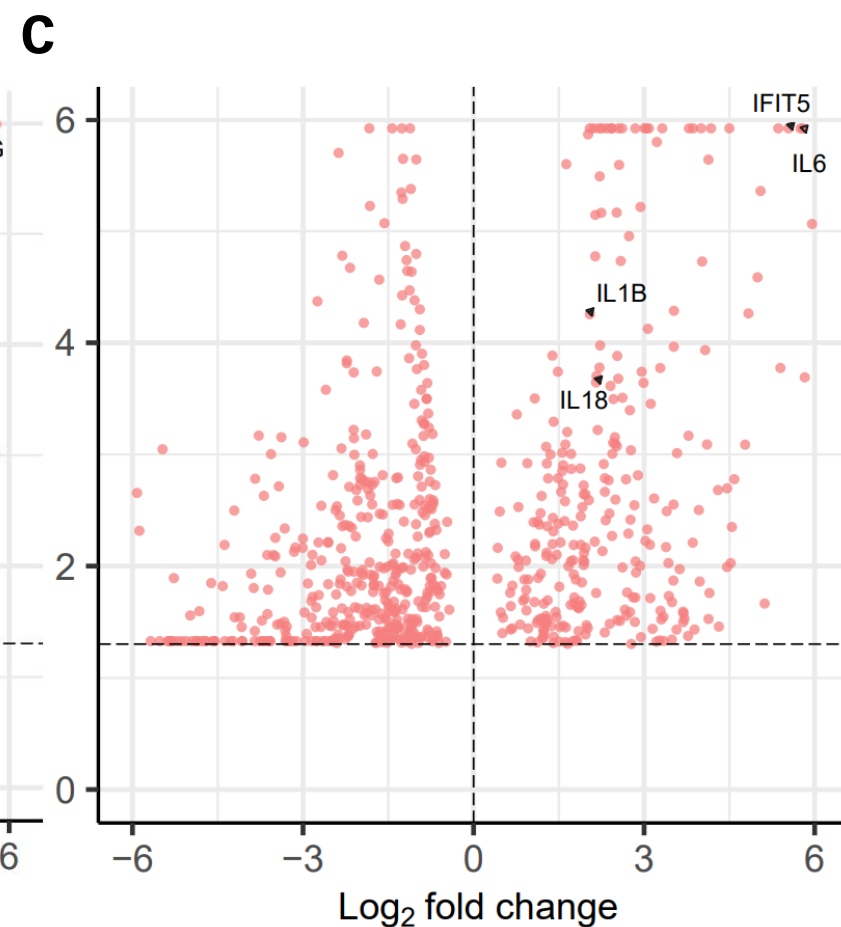

Macrophage

Supplement: S4 Fig — The figure shows the results of differential gene expression analysis (infection group vs. control group) in kidney tissues of (a) distal convoluted tubule cells, (b) CD8 T cells, and (c) macrophage clusters, comparing the infected group to the control group. (PDF) [file ppat.1012232.s004.pdf]

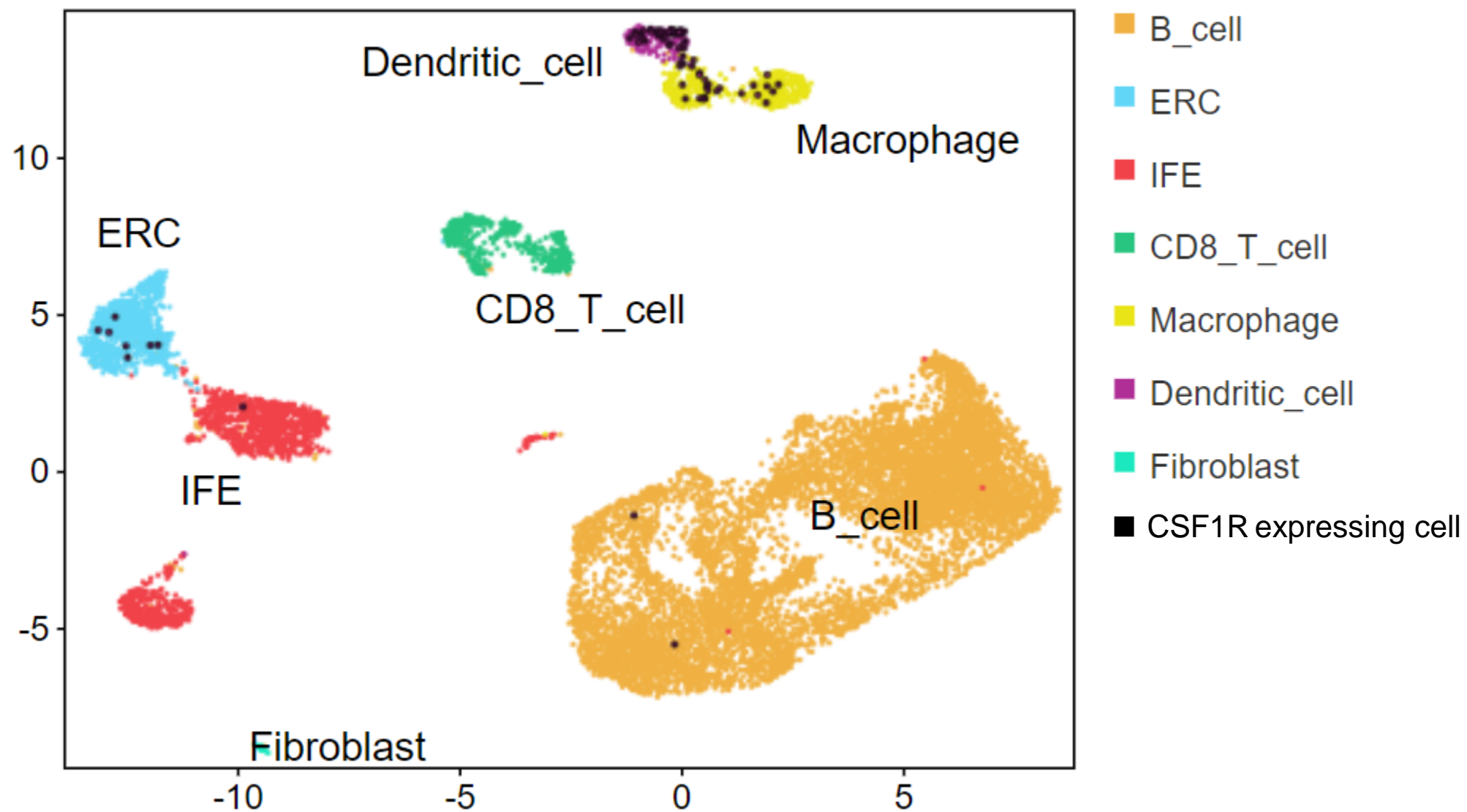

Supplement: S5 Fig — (PDF) [file ppat.1012232.s005.pdf]

a

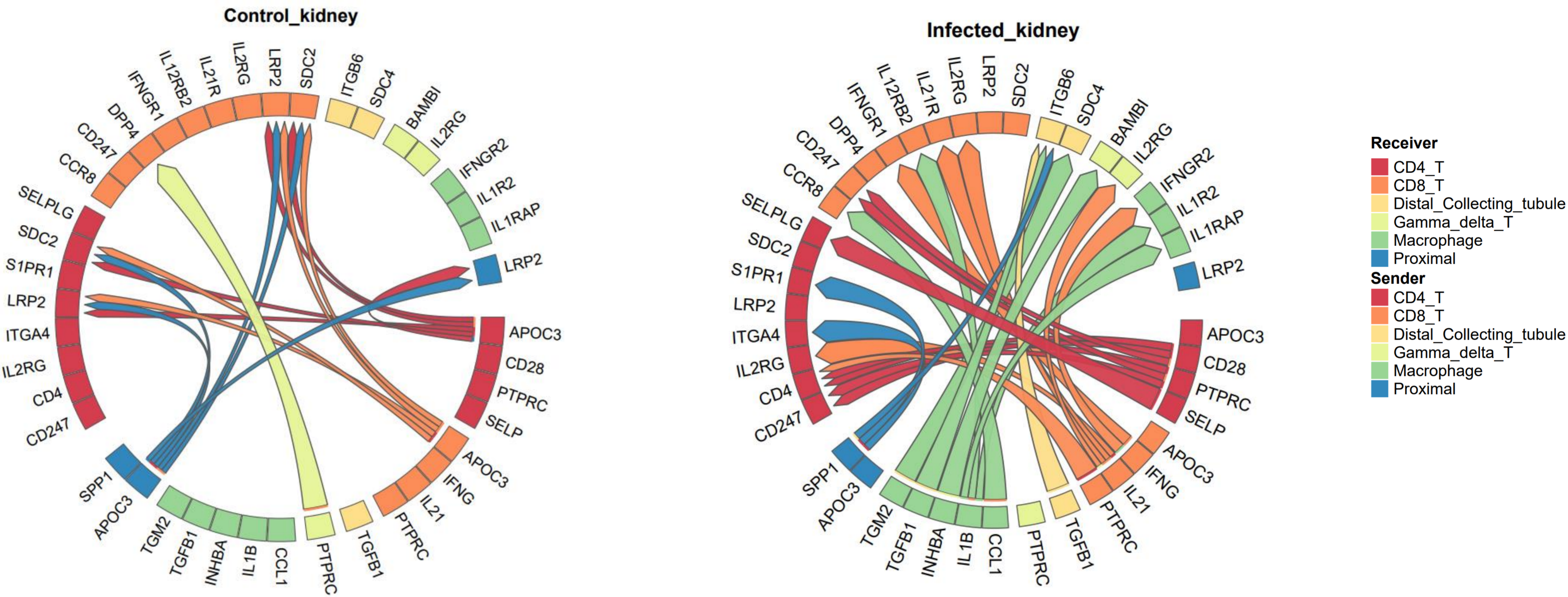

b

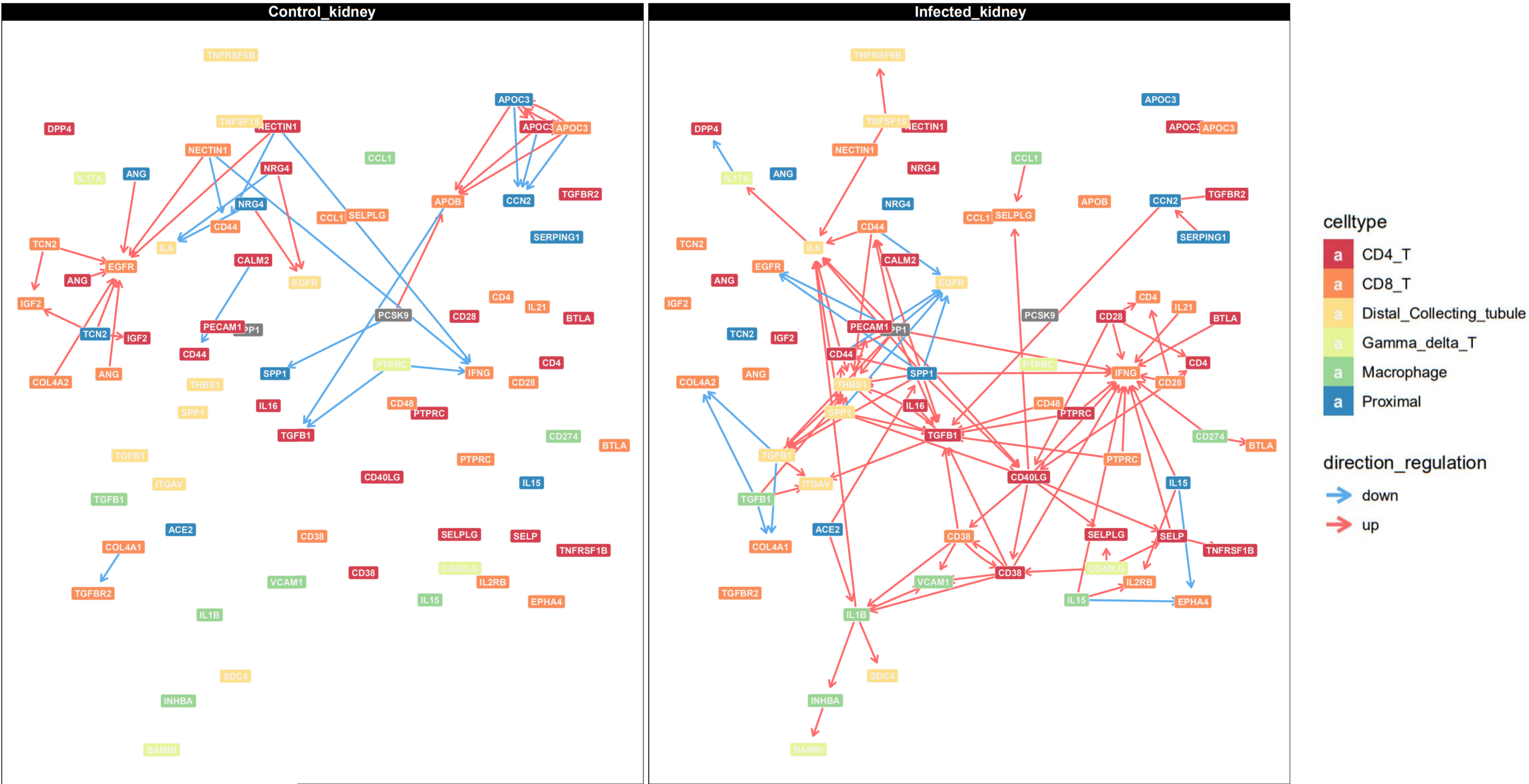

Supplement: S6 Fig — (a) The circus plot illustrates the top 40 differential ligand-receptor pairs between IBV-infected and uninfected chicken kidneys, categorized by cell type. The directionality from sender to receiver cell types is indicated by arrows, with the color of each arrow denoting the cell type expressing the ligand. (b) The inter-group differential intercellular signaling communication network predicted by MultiNicheNet. This network displays the potential divergent cell-to-cell signaling cascade patterns between IBV-infected and uninfected groups. This network is composed of predicted ligand-target associations, where "upregulated" links signify a positive correlation in expression between ligand-receptor pairs and target genes in receptor cell types. Conversely, "downregulated" links indicate a negative correlation. Target genes, identified as the top 100 genes regulated by specific ligands with the highest regulatory potential within the nichenet network, exhibit expression correlation with specific upstream ligand-receptor pairs (Pearson or Spearman correlation > 0.6). (PDF) [file ppat.1012232.s006.pdf]

a

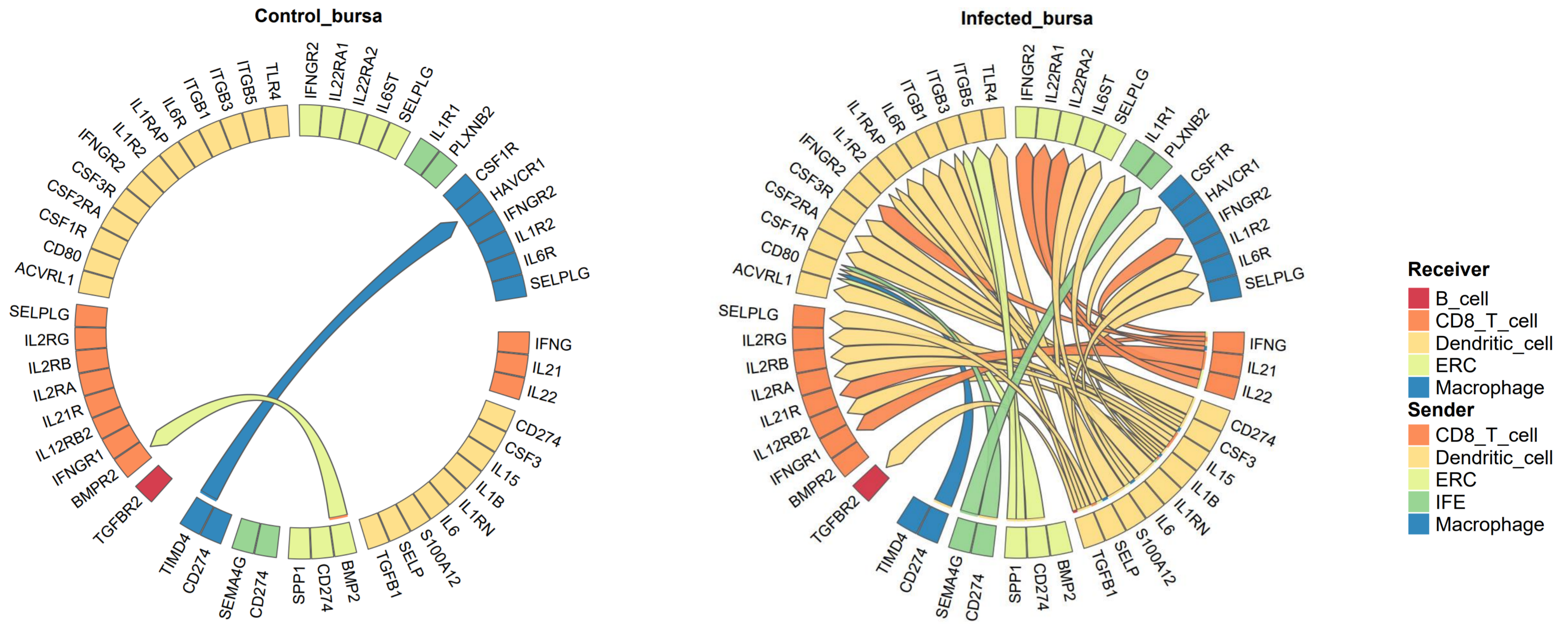

b

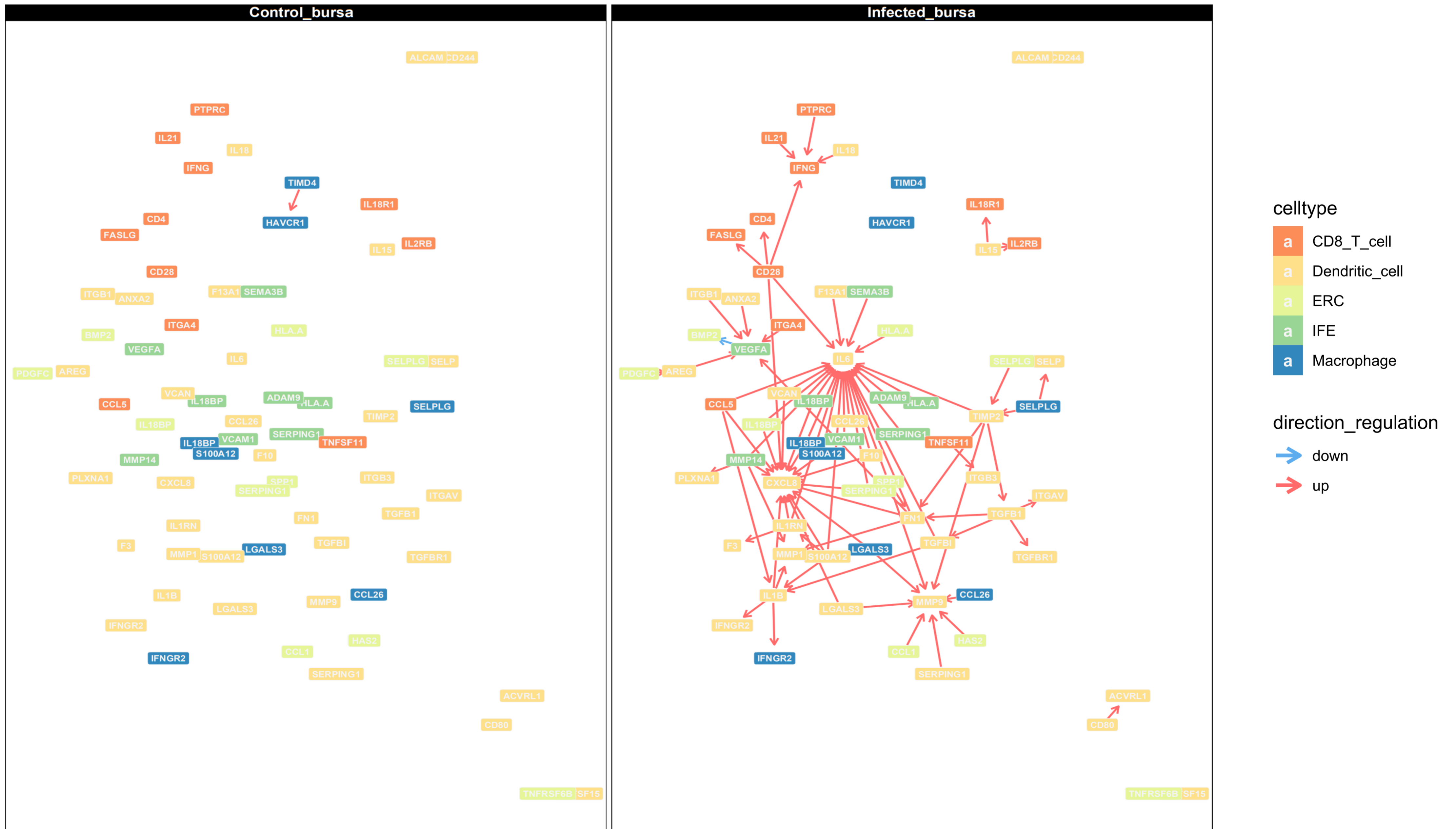

Supplement: S7 Fig — Similar to S2a Fig. (a) The circus plot illustrates the top 40 differential ligand-receptor pairs between IBV-infected and uninfected chicken bursa, categorized by cell type. (b) The inter-group differential intercellular signaling communication network predicted by MultiNicheNet. Target genes, identified as the top 50 genes regulated by specific ligands with the highest regulatory potential within the nichenet network, exhibit expression correlation with upstream ligand-receptor pairs (Pearson and Spearman correlation > 0.85). (PDF) [file ppat.1012232.s007.pdf]

**a**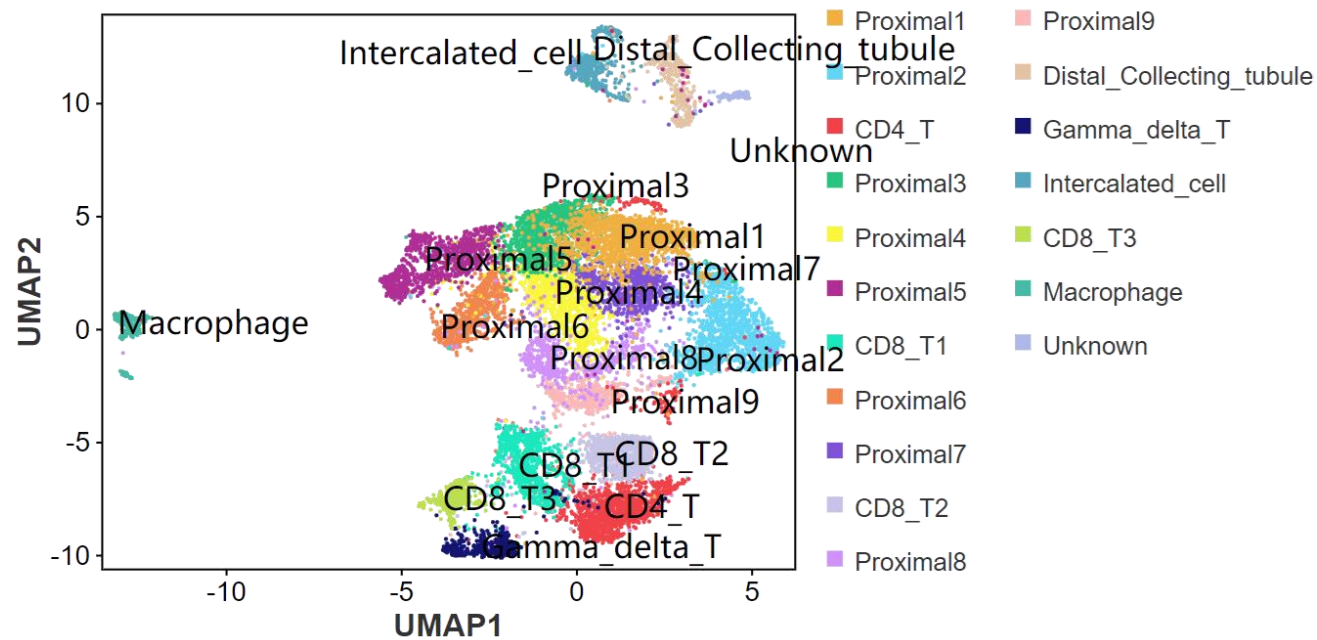**b**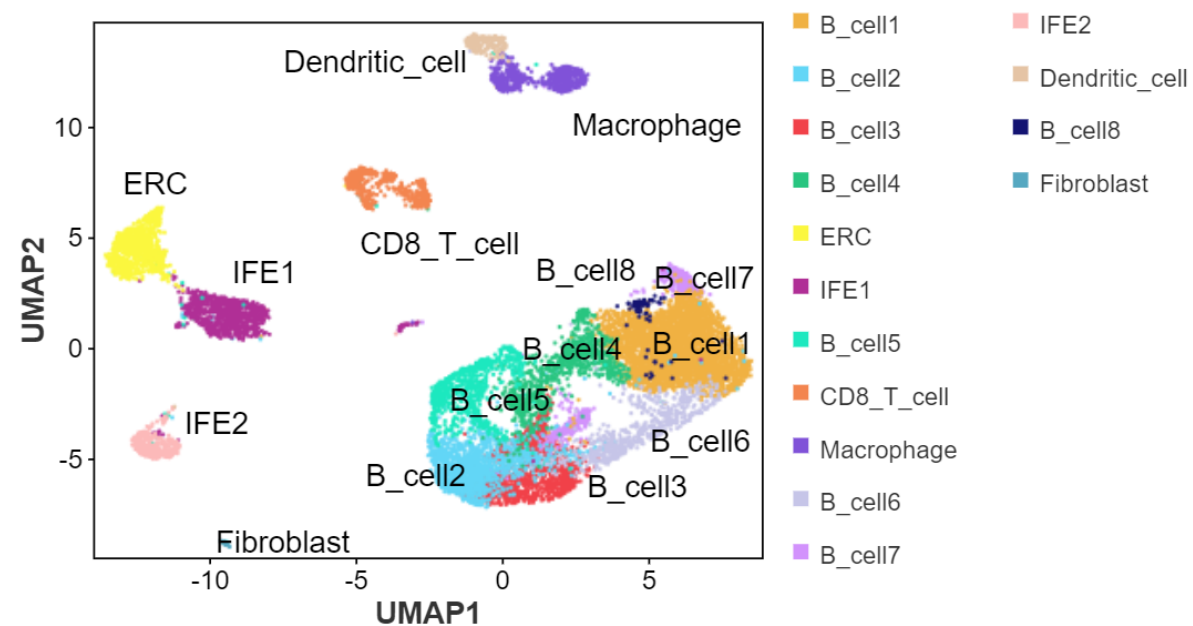**c**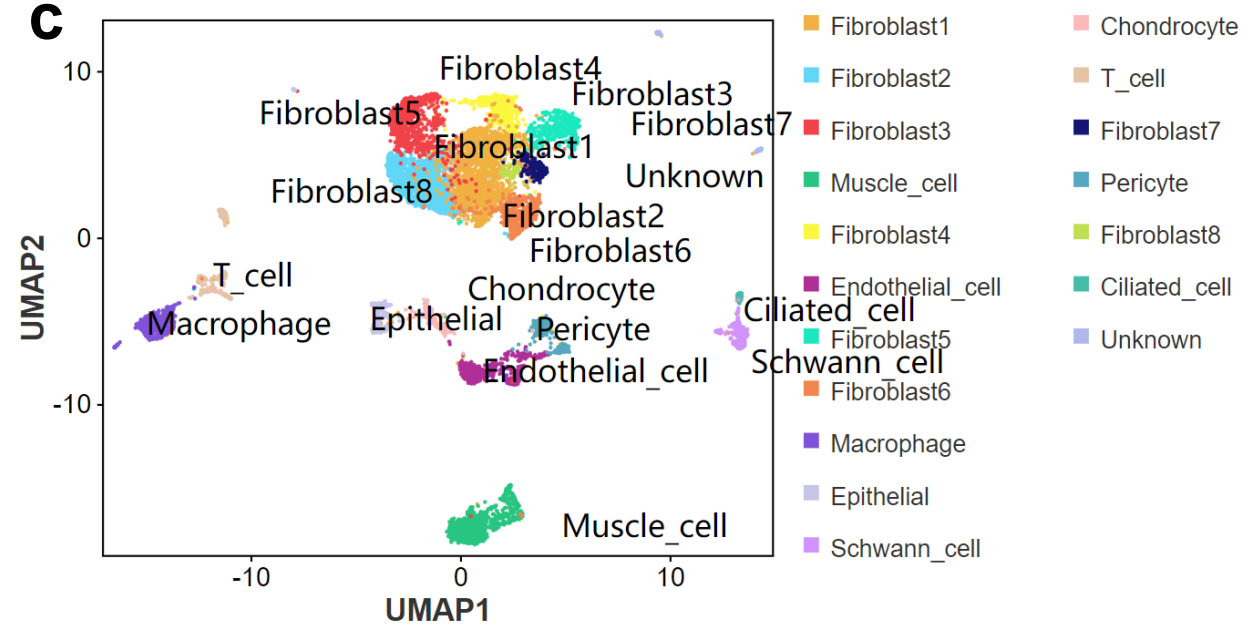

Supplement: S9 Fig — (a) In kidney, proximal tubule cells are subdivided into 9 distinct subclusters, CD8 T cells into 3 subgroups. (b) In bursa, B cells are divided into 8 subclusters. (c) In tracheal, fibroblasts are divided into 8 subclusters. (PDF) [file ppat.1012232.s009.pdf]

### Scale independence

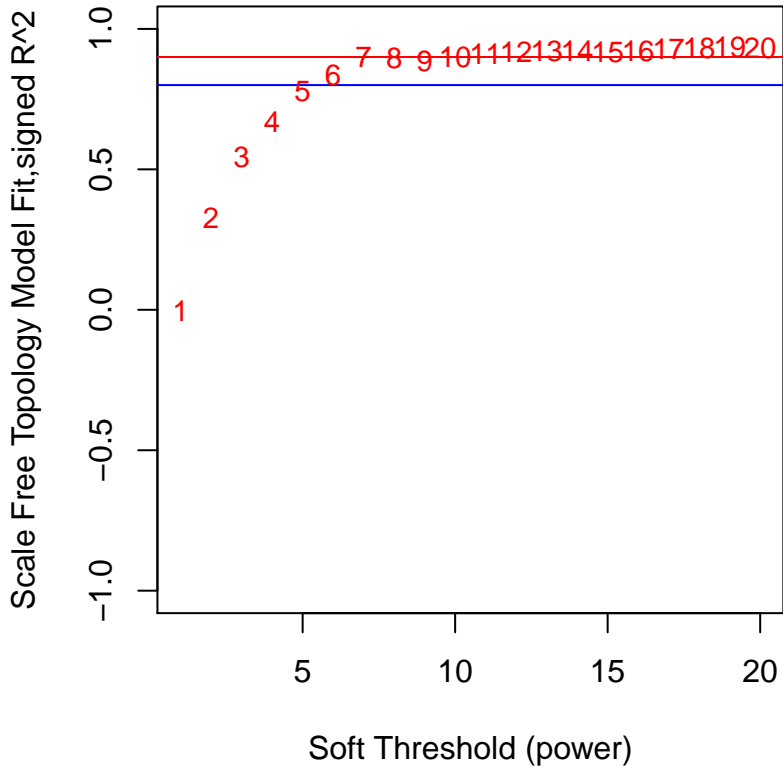

### Mean connectivity

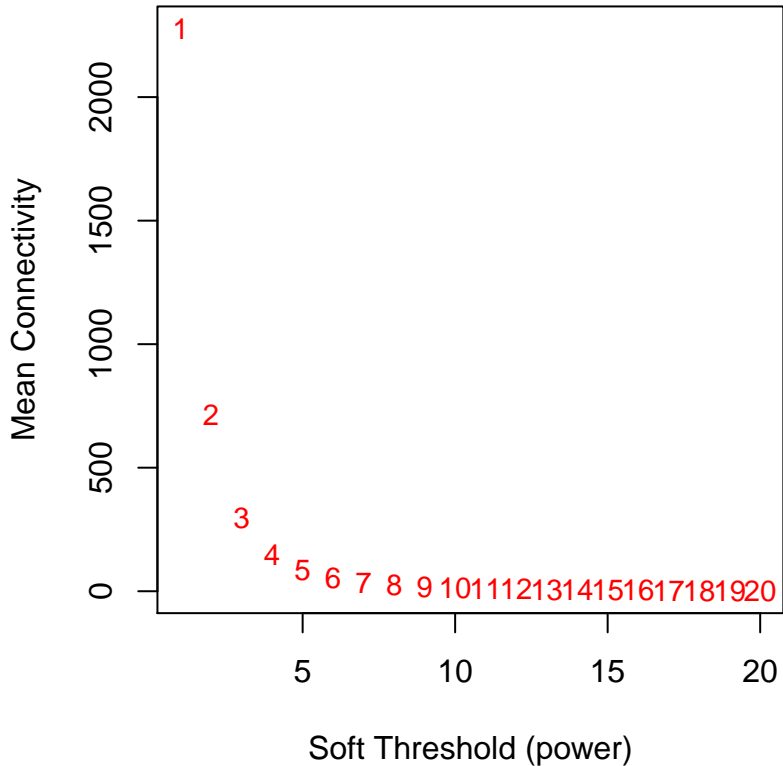

Supplement: S10 Fig — Weighted gene co-expression network showing the relationship between scale-free topology model fit (R2) and soft-thresholding power, depicted on the left; and the relationship between mean connectivity and soft-thresholding power, depicted on the right. (PDF) [file ppat.1012232.s010.pdf]

Module–Module relationships

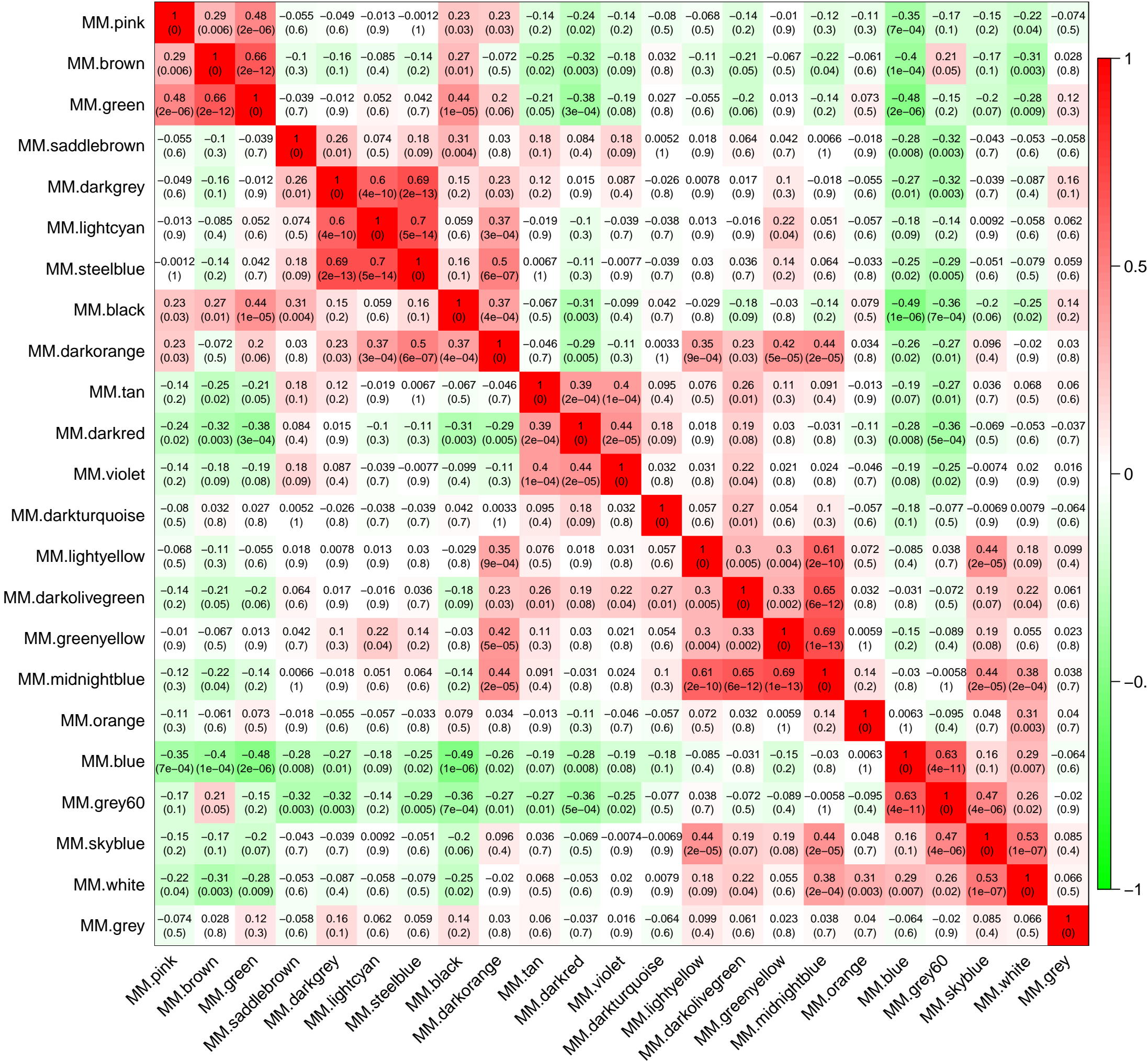

Supplement: S12 Fig — The heatmap shows the correlation coefficient among gene modules. (PDF) [file ppat.1012232.s012.pdf]

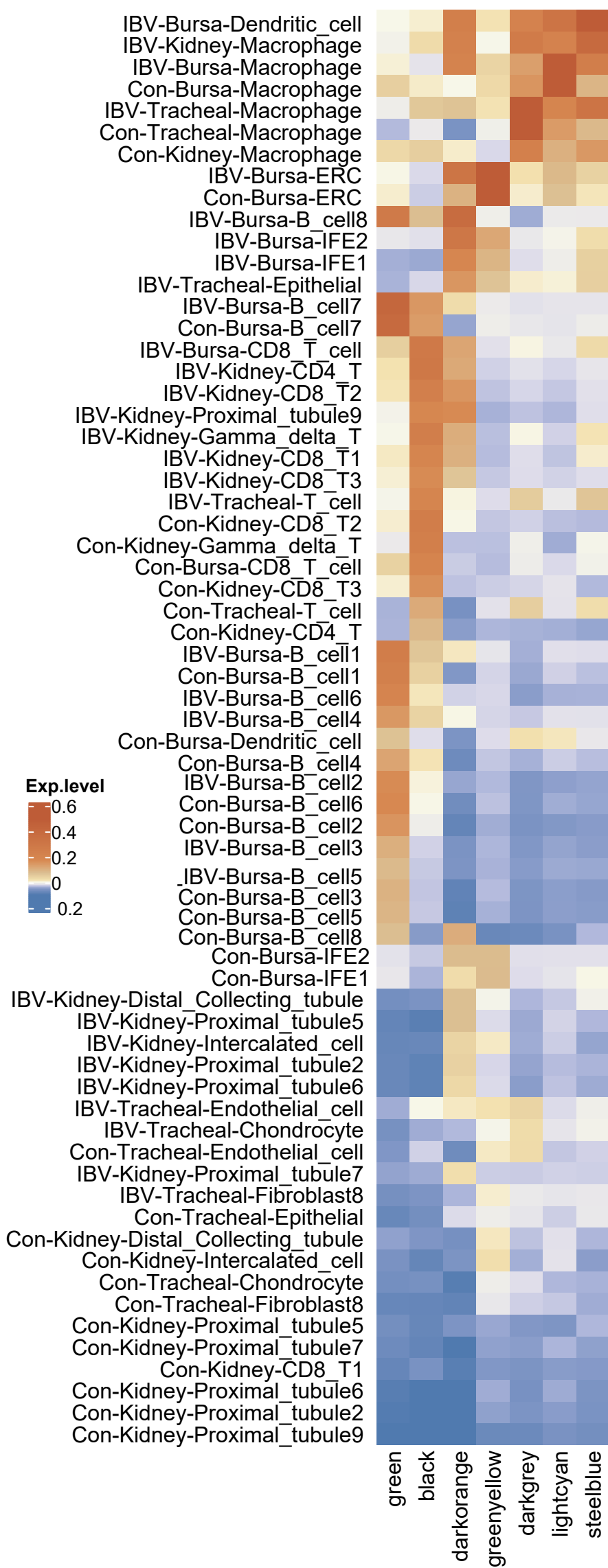

Supplement: S13 Fig — (PDF) [file ppat.1012232.s013.pdf]

# lightcyan module

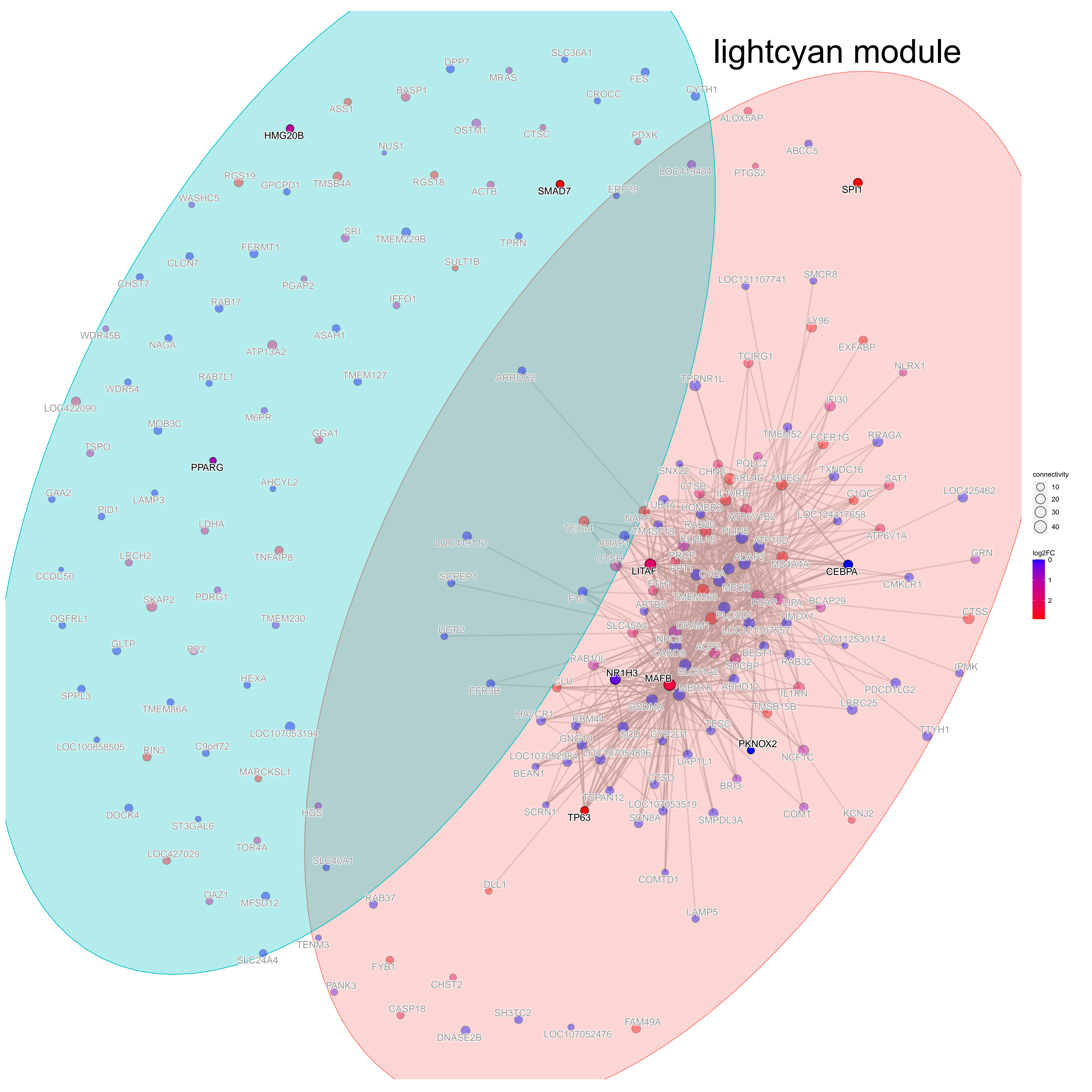

green module

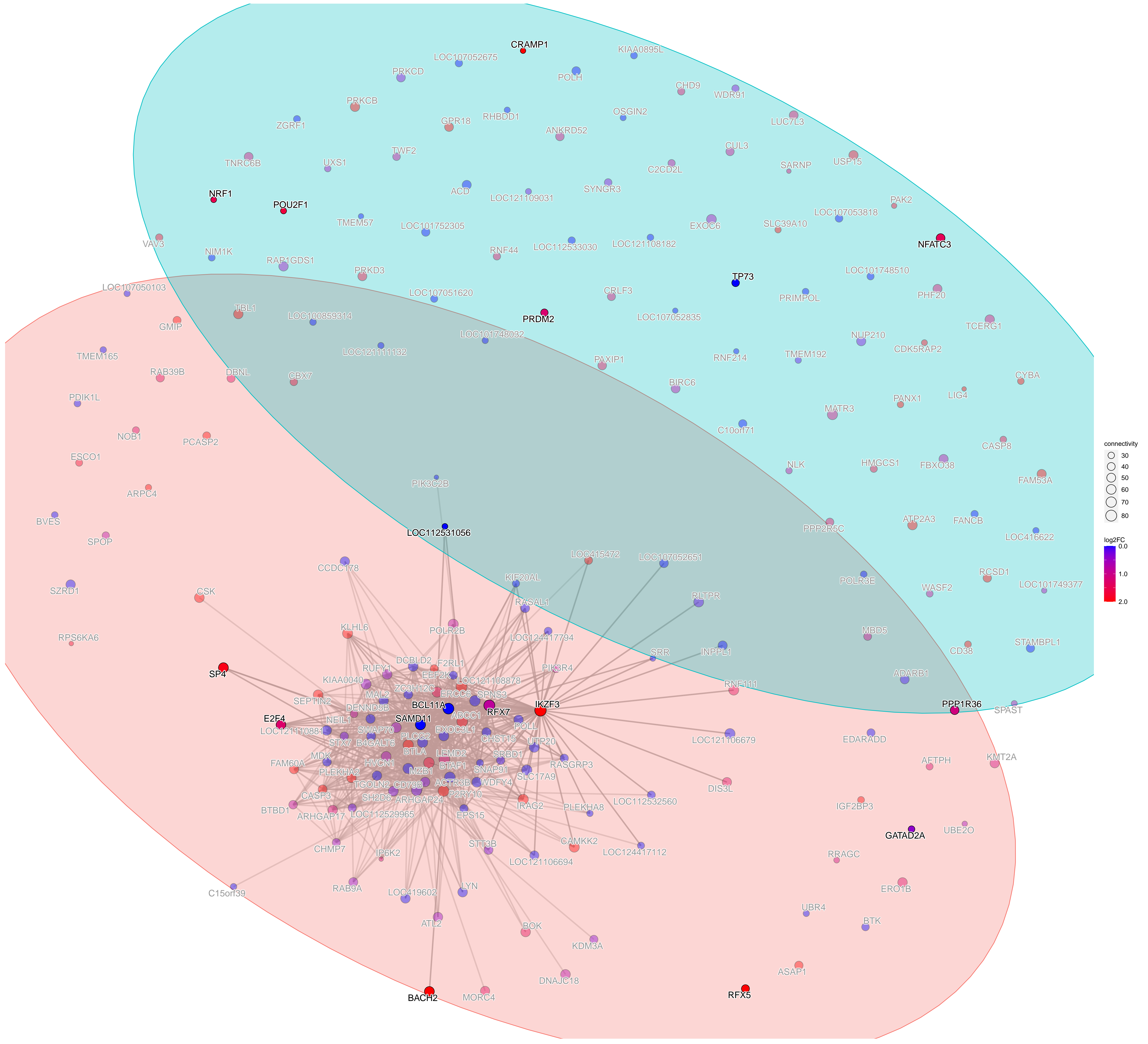

greenyellow module

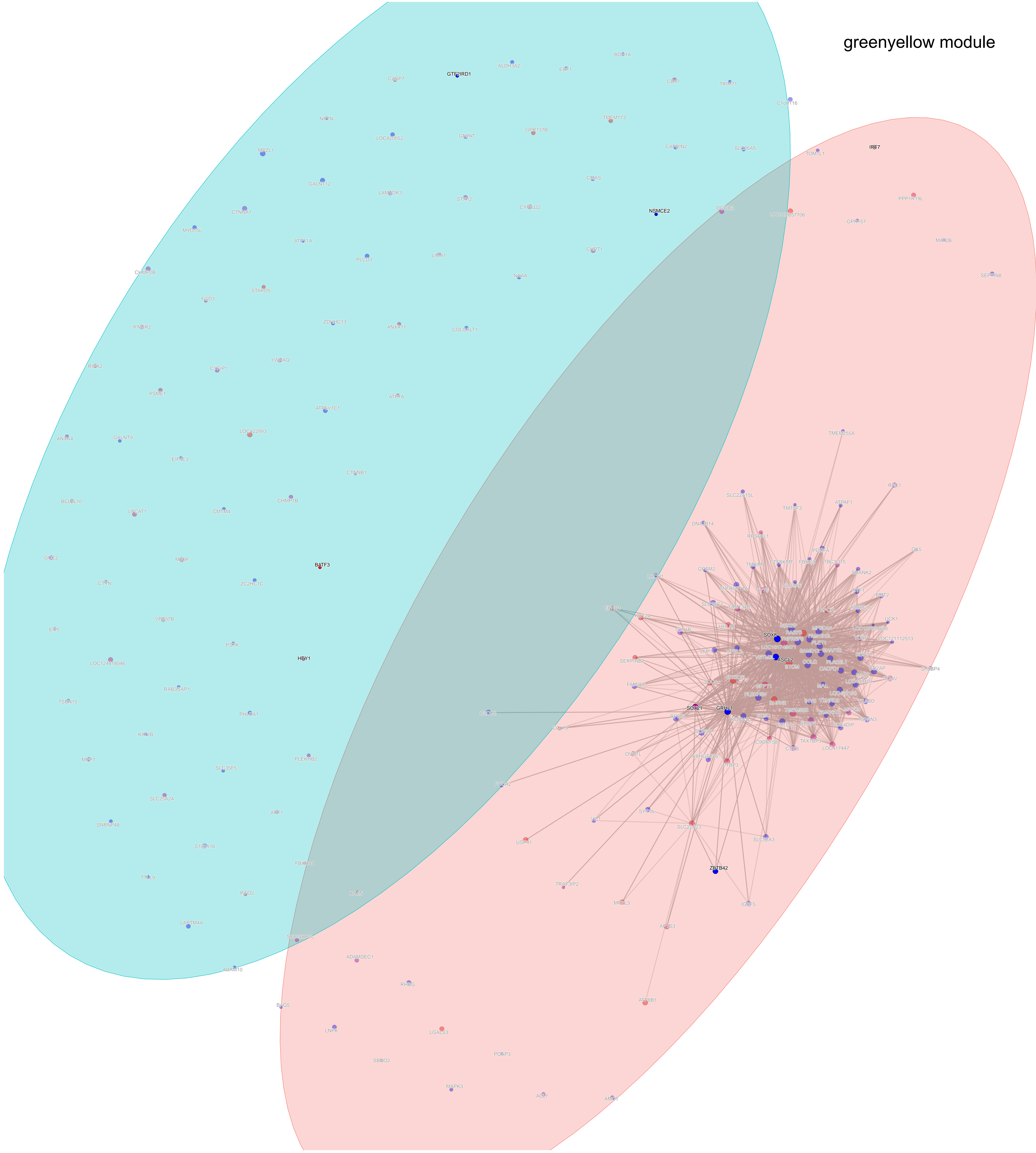

# darkgrey module

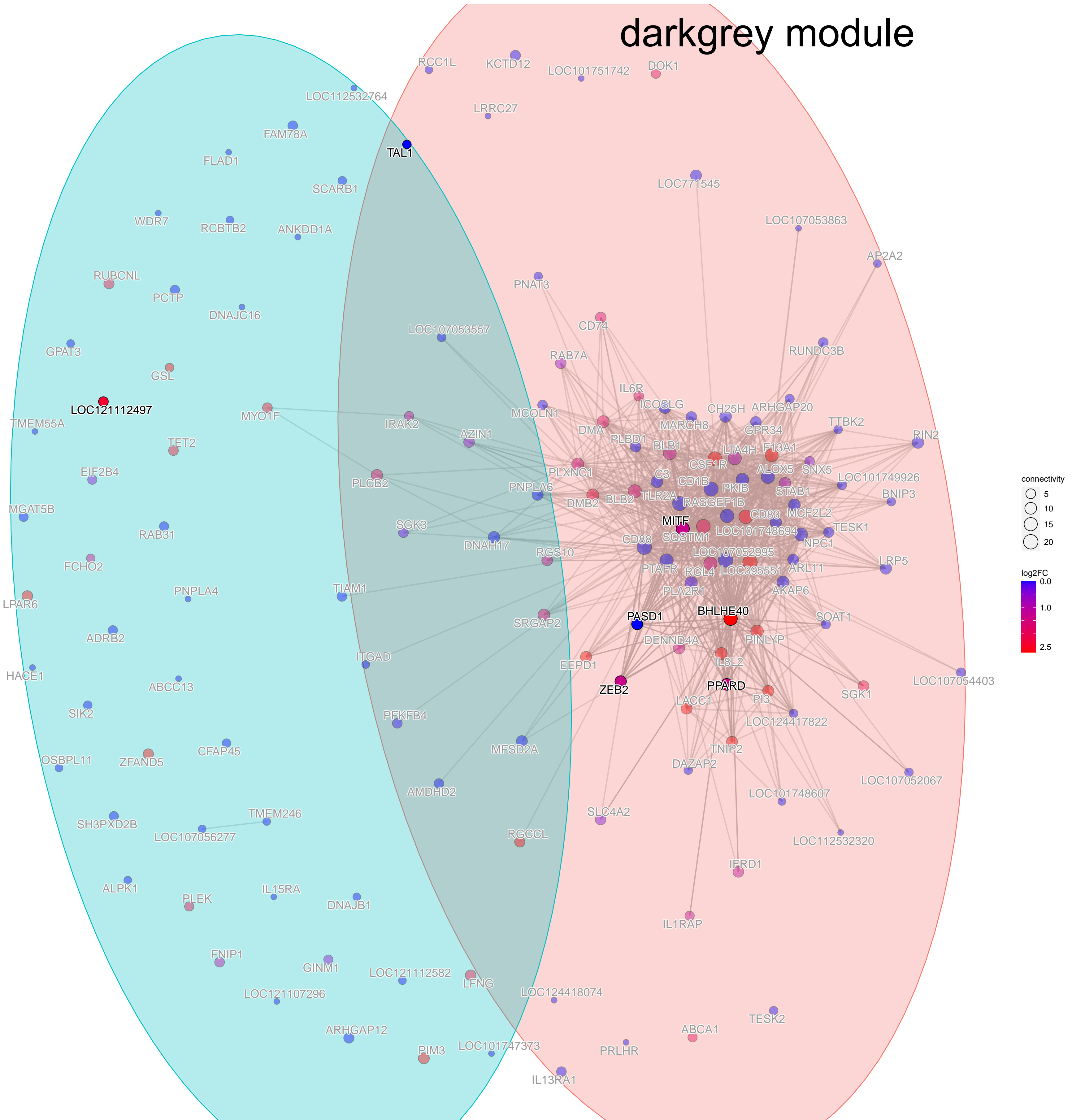

Supplement: S15 Fig — Similar to Fig 8. Network diagram displays nodes sized by gene connectivity within the module, with node color indicating the results of differential gene expression analysis(p.adjust<0.05). log2FC = log2(IBV/Control). Transcription factors are highlighted. (PDF) [file ppat.1012232.s015.pdf]
