# Supplementary material for: Deciphering infected cell types, hub gene networks and cell-cell communication in infectious bronchitis virus via single-cell RNA sequencing
Source: PLoS Pathog. 2024 May 14;20(5):e1012232. doi: 10.1371/journal.ppat.1012232 (PMC11125504; doi:10.1371/journal.ppat.1012232)
Supplement: S8 Fig — Similar to S2a Fig. (a) The circus plot illustrates the top 40 differential ligand-receptor pairs between IBV-infected and uninfected chicken trachea, categorized by cell type. (b) The inter-group differential intercellular signaling communication network predicted by MultiNicheNet. Target genes, identified as the top 250 genes regulated by specific ligands with the highest regulatory potential within the nichenet network, exhibit expression correlation with upstream ligand-receptor pairs (Pearson or Spearman correlation > 0.5). (PDF) [file ppat.1012232.s008.pdf]

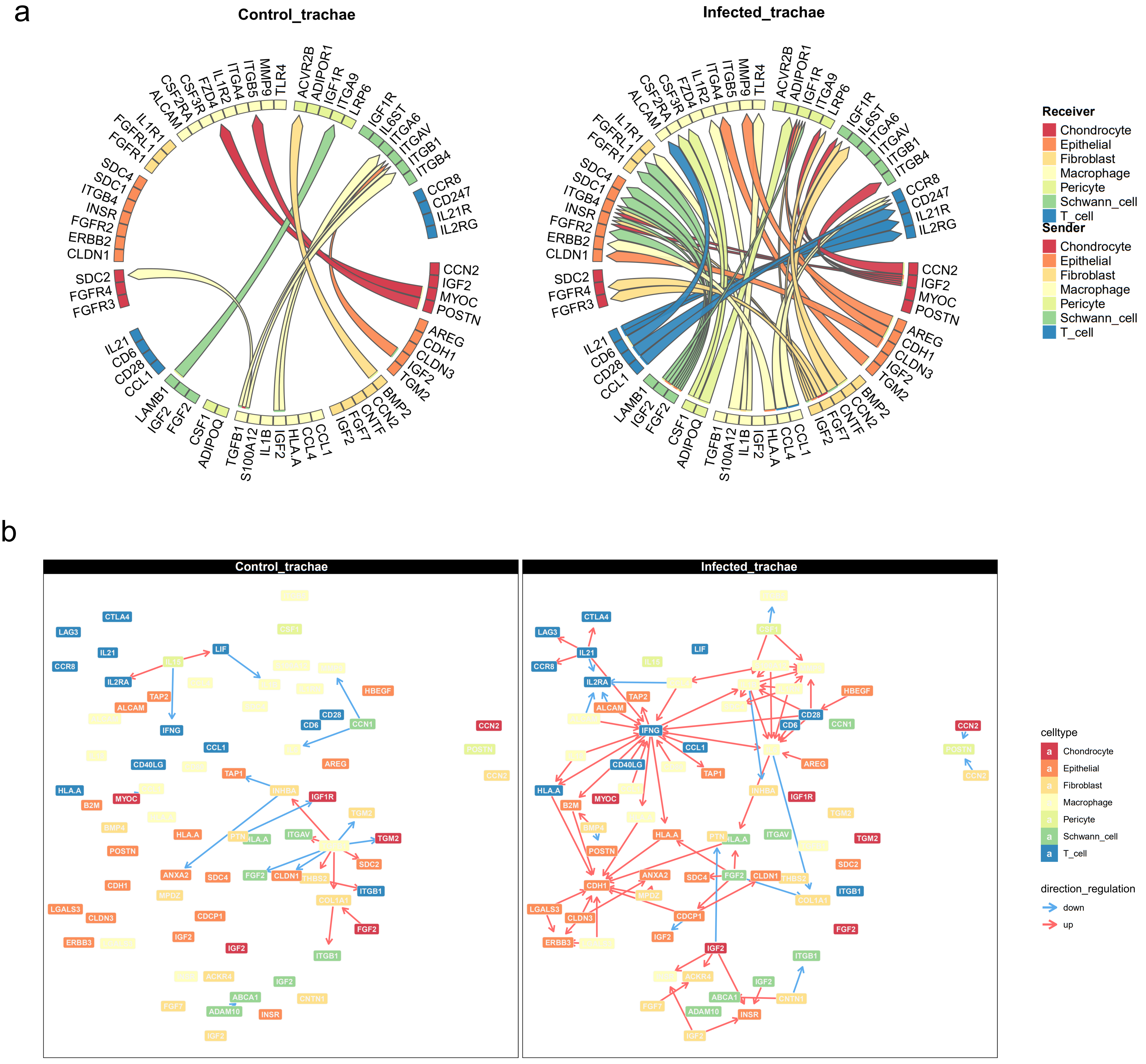
a
Control_trachae
Infected_trachae
Receiver
Chondrocyte
Epithelial
Fibroblast
Macrophage
Pericyte
Schwann_cell
T_cell
Sender
Chondrocyte
Epithelial
Fibroblast
Macrophage
Pericyte
Schwann_cell
T_cell
ACVR2B ADIPOR1 IGF1R ITGA9 LRP6
IGF1R IL6ST ITGA6 ITGAV ITGB1 ITGB4
CCR8 CD247 IL21R IL2RG
CCN2 IGF2 MYOC POSTN
AREG CDH1 CLDN3 IGF2 TGM2
BMP2 CCN2 CNTF FGF7 IGF2
CCL1 CCL4 HLA.A IGF2 IL1B S100A12 TGFB1
ADIPOQ CSF1
FGF2 IGF2 LAMB1
CCL1 CD28 CD6 IL21
FGFR3 FGFR4 SDC2
CLDN1 ERBB2 FGFR2 INSR ITGB4 SDC1 SDC4
FGFR1 FGFRL1 IL1R1
ALCAM CSF2RA CSF3R FZD4 IL1R2 ITGA4 ITGB5 MMP9 TLR4
b
Control_trachae
Infected_trachae
celltype
Chondrocyte
Epithelial
Fibroblast
Macrophage
Pericyte
Schwann_cell
T_cell
direction_regulation
down
up
CTLA4
LAG3
IL21
CCR8
IL2RA
LIF
IL15
CSF1
TAP2
ALCAM
HBEGF
CD28
CD6
CCN1
IFNG
CCN2
POSTN
CCL1
CD40LG
AREG
TAP1
HLA.A
MYOC
INHBA
IGF1R
B2M
TGM2
BMP4
HLA.A
PTN
ITGAV
POSTN
SDC2
ANXA2
SDC4
FGF2
CLDN1
THBS2
ITGB1
CDH1
MPDZ
COL1A1
LGALS3
CLDN3
CDCP1
FGF2
IGF2
IGF2
ERBB3
ITGB1
ACKR4
IGF2
ABCA1
CNTN1
FGF7
ADAM10
INSR
IGF2
