# Supplementary material for: Deciphering infected cell types, hub gene networks and cell-cell communication in infectious bronchitis virus via single-cell RNA sequencing
Source: PLoS Pathog. 2024 May 14;20(5):e1012232. doi: 10.1371/journal.ppat.1012232 (PMC11125504; doi:10.1371/journal.ppat.1012232)
Supplement: S11 Fig — (PDF) [file ppat.1012232.s011.pdf]

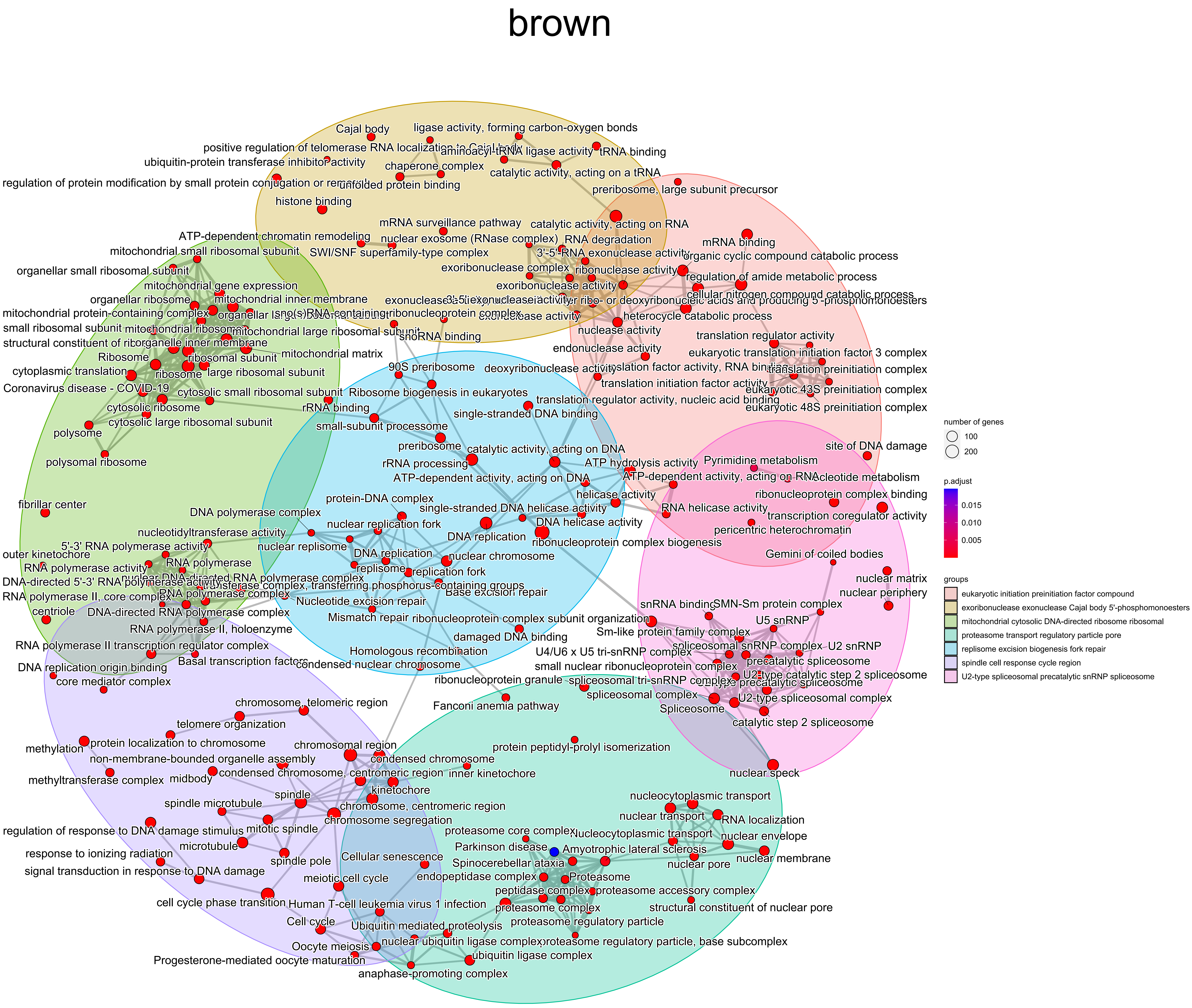

brown
Cajal body
ligase activity, forming carbon-oxygen bonds
tRNA binding
ubiquitin-protein transferase inhibitor activity
chaperone complex
catalytic activity, acting on a tRNA
unfolded protein binding
preribosome, large subunit precursor
histone binding
mRNA surveillance pathway
catalytic activity, acting on RNA
ATP-dependent chromatin remodeling
nuclear exosome (RNase complex)
RNA degradation
mRNA binding
mitochondrial small ribosomal subunit
SWI/SNF superfamily-type complex
organic cyclic compound catabolic process
organellar small ribosomal subunit
exoribonuclease complex
ribonuclease activity
regulation of amide metabolic process
mitochondrial gene expression
exoribonuclease activity
cellular nitrogen compound catabolic process
organellar ribosome
mitochondrial inner membrane
mitochondrial protein-containing complex
small ribosomal subunit
exonuclease activity
heterocycle catabolic process
snoRNA binding
nuclease activity
translation regulator activity
mitochondrial matrix
endonuclease activity
eukaryotic translation initiation factor 3 complex
Ribosome
ribosomal subunit
cytoplasmic translation
ribosome
large ribosomal subunit
90S preribosome
deoxyribonuclease activity
translation preinitiation complex
Coronavirus disease - COVID-19
translation initiation factor activity
eukaryotic 43S preinitiation complex
cytosolic small ribosomal subunit
Ribosome biogenesis in eukaryotes
cytosolic ribosome
rRNA binding
translation regulator activity, nucleic acid binding
eukaryotic 48S preinitiation complex
cytosolic large ribosomal subunit
single-stranded DNA binding
polysome
small-subunit processome
number of genes
100
200
preribosome
site of DNA damage
polysomal ribosome
catalytic activity, acting on DNA
ATP hydrolysis activity
Pyrimidine metabolism
rRNA processing
ATP-dependent activity, acting on DNA
p.adjust
helicase activity
ribonucleoprotein complex binding
0.015
fibrillar center
protein-DNA complex
single-stranded DNA helicase activity
RNA helicase activity
transcription coregulator activity
0.010
DNA polymerase complex
nuclear replication fork
DNA helicase activity
pericentric heterochromatin
0.005
nucleotidyltransferase activity
DNA replication
ribonucleoprotein complex biogenesis
5'-3' RNA polymerase activity
nuclear replisome
outer kinetochore
DNA replication
nuclear chromosome
Gemini of coiled bodies
RNA polymerase
replisome
replication fork
nuclear matrix
RNA polymerase activity
groups
eukaryotic initiation preinitiation factor compound
nuclear periphery
RNA polymerase II, core complex
Base excision repair
exoribonuclease exonuclease Cajal body 5'-phosphomonoesters
Nucleotide excision repair
snRNA binding
SMN-Sm protein complex
mitochondrial cytosolic DNA-directed ribosome ribosomal
centriole
DNA-directed RNA polymerase complex
proteasome transport regulatory particle pore
Mismatch repair
ribonucleoprotein complex subunit organization
U5 snRNP
RNA polymerase II, holoenzyme
Sm-like protein family complex
replisome excision biogenesis fork repair
damaged DNA binding
spindle cell response cycle region
RNA polymerase II transcription regulator complex
spliceosomal snRNP complex
U2 snRNP
U2-type spliceosomal precatalytic snRNP spliceosome
Homologous recombination
U4/U6 x U5 tri-snRNP complex
DNA replication origin binding
Basal transcription factors
condensed nuclear chromosome
precatalytic spliceosome
small nuclear ribonucleoprotein complex
U2-type catalytic step 2 spliceosome
core mediator complex
ribonucleoprotein granule
spliceosomal tri-snRNP complex
spliceosomal complex
U2-type spliceosomal complex
chromosome, telomeric region
Fanconi anemia pathway
Spliceosome
catalytic step 2 spliceosome
telomere organization
methylation
protein localization to chromosome
chromosomal region
protein peptidyl-prolyl isomerization
condensed chromosome
non-membrane-bounded organelle assembly
methyltransferase complex
midbody
condensed chromosome, centromeric region
inner kinetochore
nuclear speck
kinetochore
spindle
nucleocytoplasmic transport
spindle microtubule
chromosome, centromeric region
nuclear transport
RNA localization
chromosome segregation
regulation of response to DNA damage stimulus
mitotic spindle
proteasome core complex
Nucleocytoplasmic transport
nuclear envelope
microtubule
Parkinson disease
Amyotrophic lateral sclerosis
response to ionizing radiation
spindle pole
Cellular senescence
Spinocerebellar ataxia
nuclear pore
nuclear membrane
signal transduction in response to DNA damage
endopeptidase complex
Proteasome
meiotic cell cycle
peptidase complex
proteasome accessory complex
cell cycle phase transition
Human T-cell leukemia virus 1 infection
proteasome complex
structural constituent of nuclear pore
Cell cycle
Ubiquitin mediated proteolysis
proteasome regulatory particle
Oocyte meiosis
nuclear ubiquitin ligase complex
proteasome regulatory particle, base subcomplex
Progesterone-mediated oocyte maturation
ubiquitin ligase complex
anaphase-promoting complex

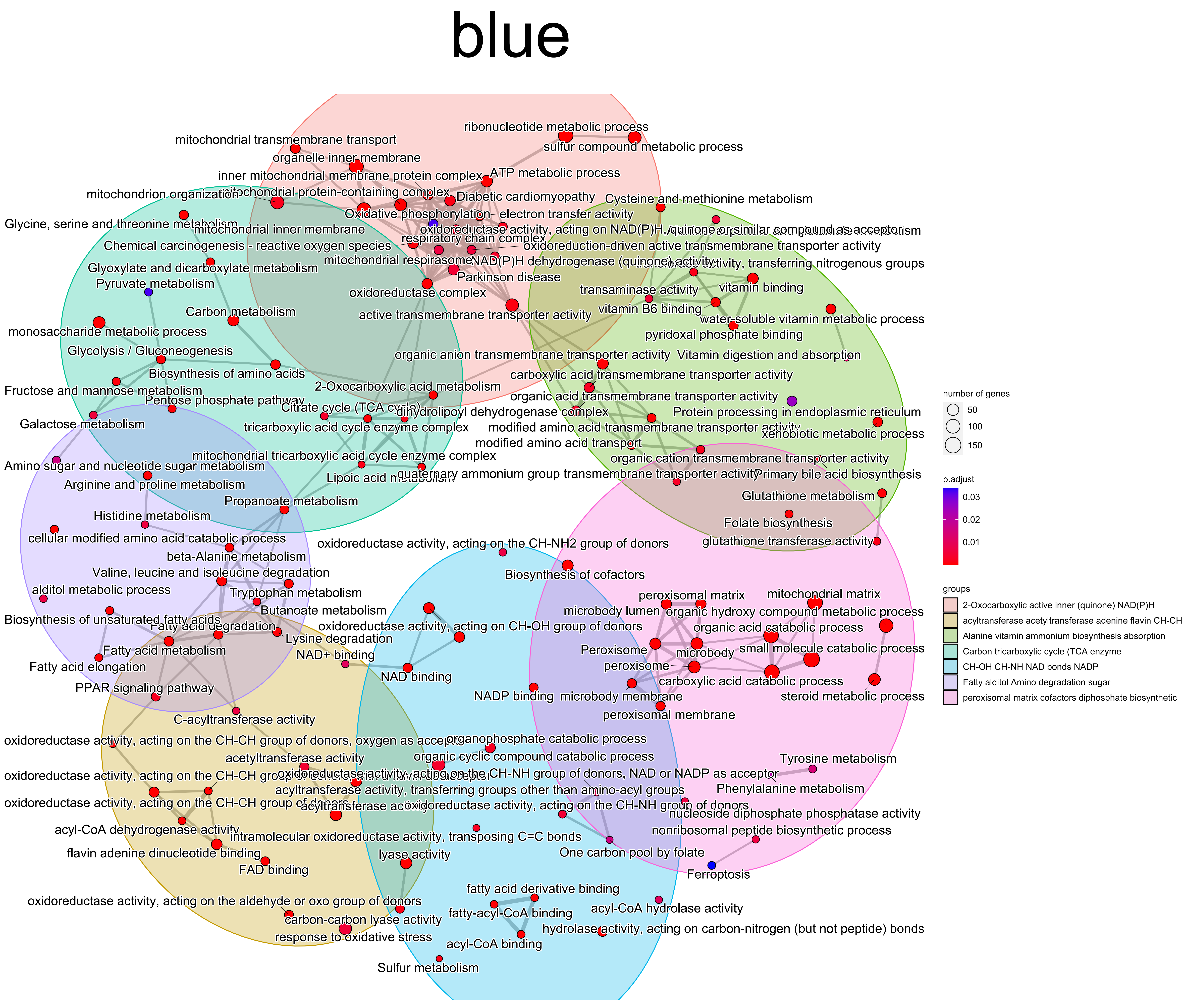

blue
ribonucleotide metabolic process
sulfur compound metabolic process
mitochondrial transmembrane transport
organelle inner membrane
inner mitochondrial membrane protein complex
ATP metabolic process
mitochondrion organization
mitochondrial protein-containing complex
Diabetic cardiomyopathy
Cysteine and methionine metabolism
Oxidative phosphorylation
electron transfer activity
Glycine, serine and threonine metabolism
mitochondrial inner membrane
oxidoreductase activity, acting on NAD(P)H, quinone or similar compound as acceptor
respiratory chain complex
Chemical carcinogenesis - reactive oxygen species
oxidoreduction-driven active transmembrane transporter activity
mitochondrial respirasome
NAD(P)H dehydrogenase (quinone) activity
transferase activity, transferring nitrogenous groups
Glyoxylate and dicarboxylate metabolism
Parkinson disease
Pyruvate metabolism
oxidoreductase complex
transaminase activity
vitamin binding
active transmembrane transporter activity
vitamin B6 binding
water-soluble vitamin metabolic process
Carbon metabolism
pyridoxal phosphate binding
monosaccharide metabolic process
Glycolysis / Gluconeogenesis
organic anion transmembrane transporter activity
Vitamin digestion and absorption
Biosynthesis of amino acids
carboxylic acid transmembrane transporter activity
Fructose and mannose metabolism
2-Oxocarboxylic acid metabolism
Pentose phosphate pathway
organic acid transmembrane transporter activity
Citrate cycle (TCA cycle)
dihydrolipoyl dehydrogenase complex
Protein processing in endoplasmic reticulum
Galactose metabolism
tricarboxylic acid cycle enzyme complex
modified amino acid transmembrane transporter activity
xenobiotic metabolic process
modified amino acid transport
mitochondrial tricarboxylic acid cycle enzyme complex
organic cation transmembrane transporter activity
Amino sugar and nucleotide sugar metabolism
quaternary ammonium group transmembrane transporter activity
Primary bile acid biosynthesis
Lipoic acid metabolism
Arginine and proline metabolism
Glutathione metabolism
Propanoate metabolism
Histidine metabolism
Folate biosynthesis
cellular modified amino acid catabolic process
glutathione transferase activity
oxidoreductase activity, acting on the CH-NH2 group of donors
beta-Alanine metabolism
Biosynthesis of cofactors
Valine, leucine and isoleucine degradation
alditol metabolic process
Tryptophan metabolism
peroxisomal matrix
mitochondrial matrix
microbody lumen
organic hydroxy compound metabolic process
Butanoate metabolism
Biosynthesis of unsaturated fatty acids
oxidoreductase activity, acting on CH-OH group of donors
organic acid catabolic process
Fatty acid degradation
Lysine degradation
Peroxisome
microbody
small molecule catabolic process
Fatty acid metabolism
NAD+ binding
peroxisome
Fatty acid elongation
NAD binding
carboxylic acid catabolic process
NADP binding
microbody membrane
steroid metabolic process
PPAR signaling pathway
peroxisomal membrane
C-acyltransferase activity
organophosphate catabolic process
oxidoreductase activity, acting on the CH-CH group of donors, oxygen as acceptor
organic cyclic compound catabolic process
acetyltransferase activity
Tyrosine metabolism
oxidoreductase activity, acting on the CH-NH group of donors, NAD or NADP as acceptor
acyltransferase activity, transferring groups other than amino-acyl groups
Phenylalanine metabolism
oxidoreductase activity, acting on the CH-CH group of donors
oxidoreductase activity, acting on the CH-NH group of donors
nucleoside diphosphate phosphatase activity
acyl-CoA dehydrogenase activity
nonribosomal peptide biosynthetic process
intramolecular oxidoreductase activity, transposing C=C bonds
flavin adenine dinucleotide binding
One carbon pool by folate
lyase activity
FAD binding
Ferroptosis
fatty acid derivative binding
oxidoreductase activity, acting on the aldehyde or oxo group of donors
acyl-CoA hydrolase activity
fatty-acyl-CoA binding
carbon-carbon lyase activity
hydrolase activity, acting on carbon-nitrogen (but not peptide) bonds
response to oxidative stress
acyl-CoA binding
Sulfur metabolism
number of genes
50
100
150
p.adjust
0.03
0.02
0.01
groups
2-Oxocarboxylic active inner (quinone) NAD(P)H
acyltransferase acetyltransferase adenine flavin CH-CH
Alanine vitamin ammonium biosynthesis absorption
Carbon tricarboxylic cycle (TCA enzyme
CH-OH CH-NH NAD bonds NADP
Fatty alditol Amino degradation sugar
peroxisomal matrix cofactors diphosphate biosynthetic

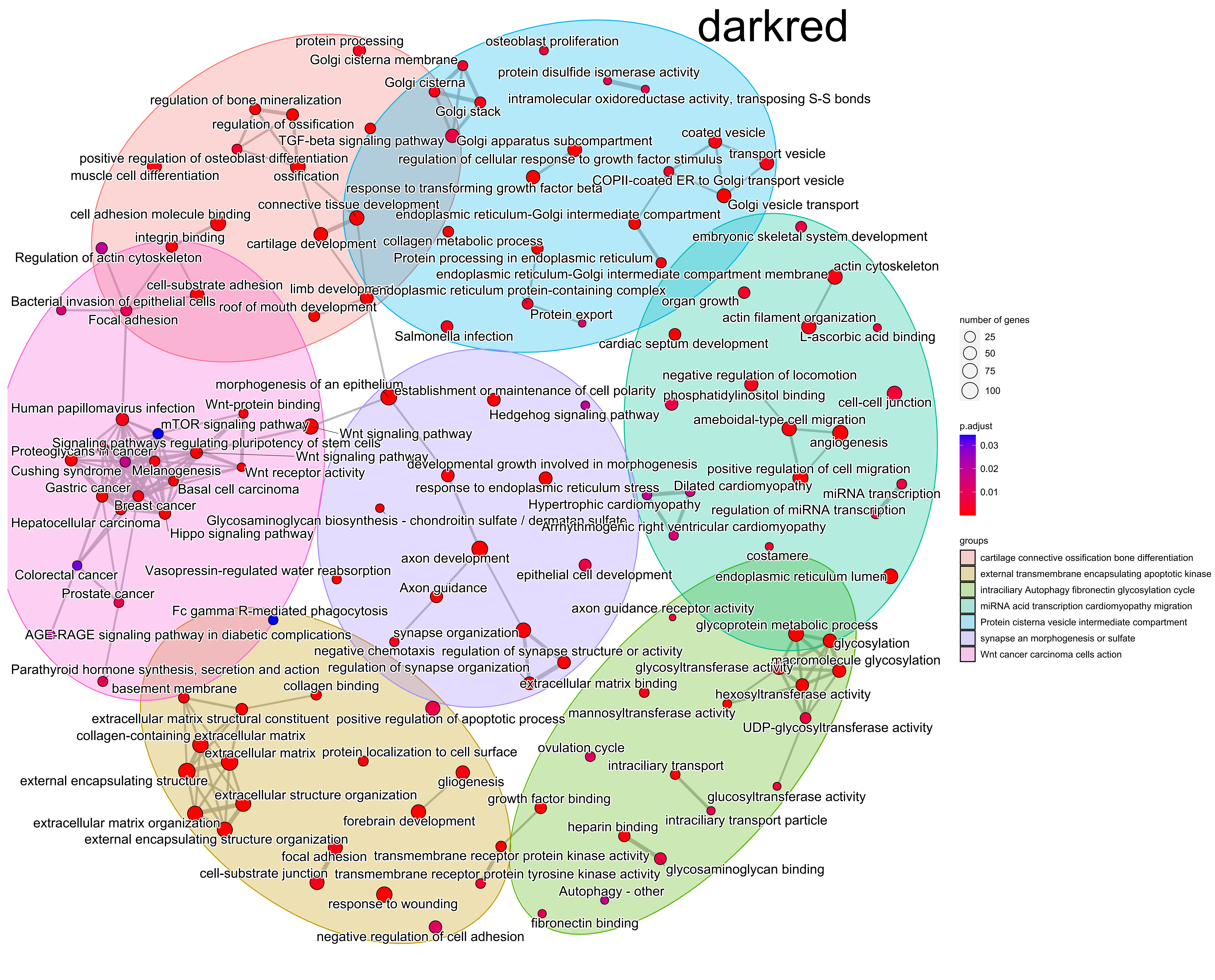

darkred
protein processing
osteoblast proliferation
Golgi cisterna membrane
protein disulfide isomerase activity
Golgi cisterna
intramolecular oxidoreductase activity, transposing S-S bonds
regulation of bone mineralization
Golgi stack
regulation of ossification
TGF-beta signaling pathway
Golgi apparatus subcompartment
coated vesicle
positive regulation of osteoblast differentiation
regulation of cellular response to growth factor stimulus
transport vesicle
muscle cell differentiation
ossification
COPII-coated ER to Golgi transport vesicle
response to transforming growth factor beta
connective tissue development
Golgi vesicle transport
cell adhesion molecule binding
endoplasmic reticulum-Golgi intermediate compartment
integrin binding
cartilage development
collagen metabolic process
embryonic skeletal system development
Regulation of actin cytoskeleton
Protein processing in endoplasmic reticulum
endoplasmic reticulum-Golgi intermediate compartment membrane
actin cytoskeleton
cell-substrate adhesion
limb development
endoplasmic reticulum protein-containing complex
Bacterial invasion of epithelial cells
roof of mouth development
organ growth
Focal adhesion
Protein export
actin filament organization
Salmonella infection
cardiac septum development
L-ascorbic acid binding
negative regulation of locomotion
morphogenesis of an epithelium
establishment or maintenance of cell polarity
phosphatidylinositol binding
Human papillomavirus infection
Wnt-protein binding
Hedgehog signaling pathway
cell-cell junction
mTOR signaling pathway
ameboidal-type cell migration
Wnt signaling pathway
angiogenesis
Signaling pathways regulating pluripotency of stem cells
Proteoglycans in cancer
Wnt signaling pathway
Wnt receptor activity
developmental growth involved in morphogenesis
positive regulation of cell migration
Cushing syndrome
Melanogenesis
Dilated cardiomyopathy
Basal cell carcinoma
response to endoplasmic reticulum stress
miRNA transcription
Gastric cancer
Hypertrophic cardiomyopathy
Breast cancer
regulation of miRNA transcription
Glycosaminoglycan biosynthesis - chondroitin sulfate / dermatan sulfate
Hepatocellular carcinoma
Arrhythmogenic right ventricular cardiomyopathy
Hippo signaling pathway
costamere
axon development
Colorectal cancer
Vasopressin-regulated water reabsorption
epithelial cell development
endoplasmic reticulum lumen
Axon guidance
Prostate cancer
axon guidance receptor activity
Fc gamma R-mediated phagocytosis
glycoprotein metabolic process
synapse organization
AGE-RAGE signaling pathway in diabetic complications
glycosylation
negative chemotaxis
regulation of synapse structure or activity
macromolecule glycosylation
Parathyroid hormone synthesis, secretion and action
regulation of synapse organization
glycosyltransferase activity
basement membrane
collagen binding
extracellular matrix binding
hexosyltransferase activity
extracellular matrix structural constituent
positive regulation of apoptotic process
mannosyltransferase activity
collagen-containing extracellular matrix
UDP-glycosyltransferase activity
extracellular matrix
protein localization to cell surface
ovulation cycle
external encapsulating structure
intraciliary transport
gliogenesis
extracellular structure organization
glucosyltransferase activity
growth factor binding
extracellular matrix organization
forebrain development
intraciliary transport particle
heparin binding
external encapsulating structure organization
focal adhesion
transmembrane receptor protein kinase activity
glycosaminoglycan binding
cell-substrate junction
transmembrane receptor protein tyrosine kinase activity
Autophagy - other
response to wounding
fibronectin binding
negative regulation of cell adhesion
number of genes
25
50
75
100
p.adjust
0.03
0.02
0.01
groups
cartilage connective ossification bone differentiation
external transmembrane encapsulating apoptotic kinase
intraciliary Autophagy fibronectin glycosylation cycle
miRNA acid transcription cardiomyopathy migration
Protein cisterna vesicle intermediate compartment
synapse an morphogenesis or sulfate
Wnt cancer carcinoma cells action

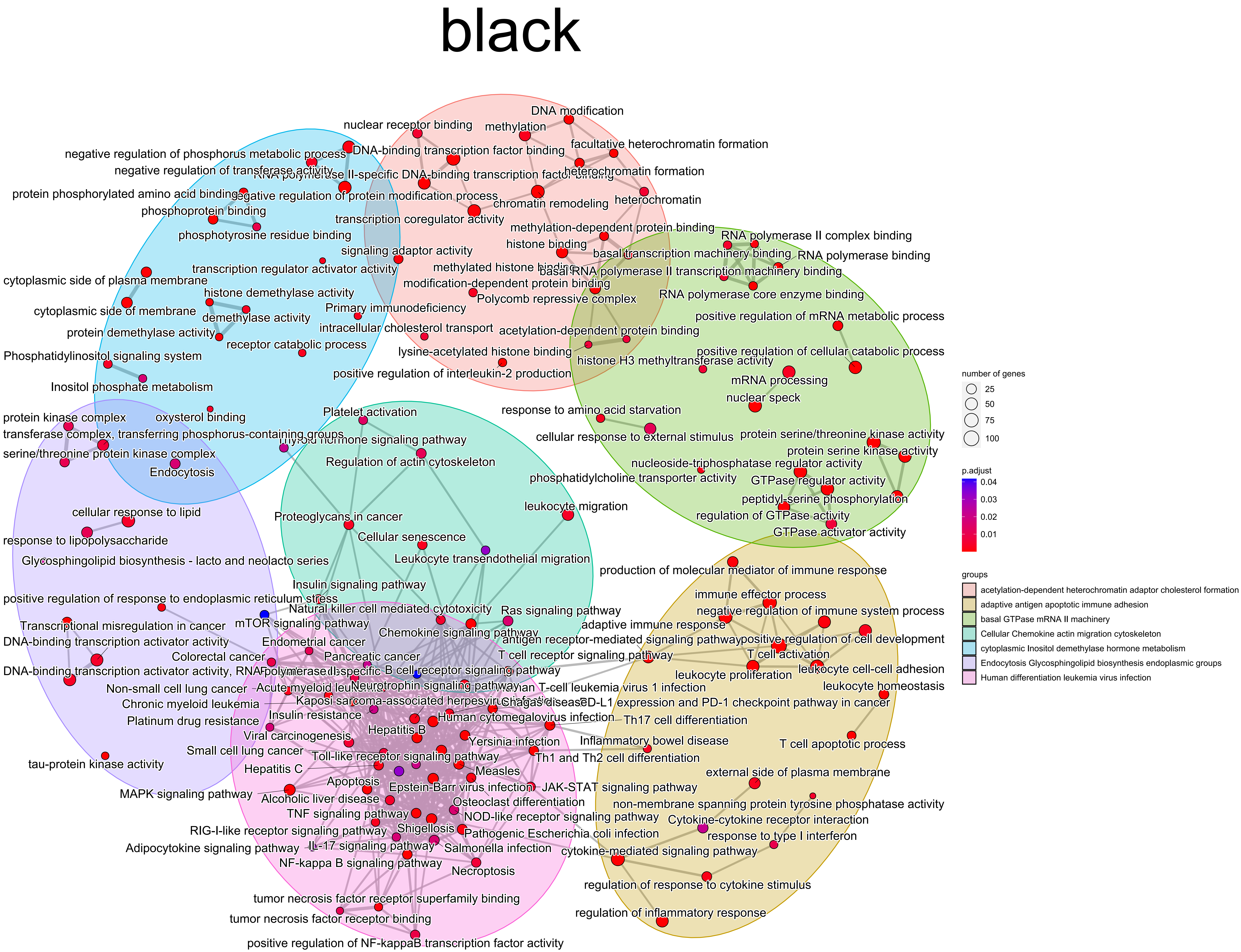
black
nuclear receptor binding
DNA modification
methylation
facultative heterochromatin formation
negative regulation of phosphorus metabolic process
DNA-binding transcription factor binding
negative regulation of transferase activity
heterochromatin formation
protein phosphorylated amino acid binding
negative regulation of protein modification process
chromatin remodeling
heterochromatin
phosphoprotein binding
transcription coregulator activity
phosphotyrosine residue binding
methylation-dependent protein binding
RNA polymerase II complex binding
histone binding
basal transcription machinery binding
RNA polymerase binding
signaling adaptor activity
transcription regulator activator activity
methylated histone binding
basal RNA polymerase II transcription machinery binding
cytoplasmic side of plasma membrane
modification-dependent protein binding
histone demethylase activity
Polycomb repressive complex
RNA polymerase core enzyme binding
cytoplasmic side of membrane
Primary immunodeficiency
demethylase activity
positive regulation of mRNA metabolic process
protein demethylase activity
intracellular cholesterol transport
acetylation-dependent protein binding
receptor catabolic process
lysine-acetylated histone binding
positive regulation of cellular catabolic process
Phosphatidylinositol signaling system
histone H3 methyltransferase activity
positive regulation of interleukin-2 production
mRNA processing
Inositol phosphate metabolism
nuclear speck
Platelet activation
response to amino acid starvation
protein kinase complex
oxysterol binding
cellular response to external stimulus
protein serine/threonine kinase activity
transferase complex, transferring phosphorus-containing groups
protein serine kinase activity
serine/threonine protein kinase complex
Regulation of actin cytoskeleton
nucleoside-triphosphatase regulator activity
Endocytosis
phosphatidylcholine transporter activity
GTPase regulator activity
peptidyl-serine phosphorylation
leukocyte migration
cellular response to lipid
regulation of GTPase activity
Proteoglycans in cancer
GTPase activator activity
response to lipopolysaccharide
Cellular senescence
Leukocyte transendothelial migration
Glycosphingolipid biosynthesis - lacto and neolacto series
production of molecular mediator of immune response
Insulin signaling pathway
positive regulation of response to endoplasmic reticulum stress
immune effector process
Natural killer cell mediated cytotoxicity
negative regulation of immune system process
Ras signaling pathway
mTOR signaling pathway
adaptive immune response
Transcriptional misregulation in cancer
Chemokine signaling pathway
antigen receptor-mediated signaling pathway
positive regulation of cell development
DNA-binding transcription activator activity
Endometrial cancer
T cell receptor signaling pathway
T cell activation
Colorectal cancer
Pancreatic cancer
leukocyte cell-cell adhesion
B cell receptor signaling pathway
leukocyte proliferation
Non-small cell lung cancer
Acute myeloid leukemia
Neurotrophin signaling pathway
leukocyte homeostasis
Chronic myeloid leukemia
Kaposi sarcoma-associated herpesvirus infection
Chagas disease
PD-L1 expression and PD-1 checkpoint pathway in cancer
Insulin resistance
Human cytomegalovirus infection
Th17 cell differentiation
Platinum drug resistance
Viral carcinogenesis
Hepatitis B
Yersinia infection
Inflammatory bowel disease
T cell apoptotic process
Small cell lung cancer
Toll-like receptor signaling pathway
Th1 and Th2 cell differentiation
tau-protein kinase activity
Hepatitis C
Measles
external side of plasma membrane
Apoptosis
Epstein-Barr virus infection
JAK-STAT signaling pathway
MAPK signaling pathway
Alcoholic liver disease
Osteoclast differentiation
non-membrane spanning protein tyrosine phosphatase activity
TNF signaling pathway
NOD-like receptor signaling pathway
Cytokine-cytokine receptor interaction
RIG-I-like receptor signaling pathway
Shigellosis
Pathogenic Escherichia coli infection
response to type I interferon
Adipocytokine signaling pathway
IL-17 signaling pathway
Salmonella infection
cytokine-mediated signaling pathway
NF-kappa B signaling pathway
Necroptosis
regulation of response to cytokine stimulus
tumor necrosis factor receptor superfamily binding
tumor necrosis factor receptor binding
regulation of inflammatory response
positive regulation of NF-kappaB transcription factor activity
number of genes
25
50
75
100
p.adjust
0.04
0.03
0.02
0.01
groups
acetylation-dependent heterochromatin adaptor cholesterol formation
adaptive antigen apoptotic immune adhesion
basal GTPase mRNA II machinery
Cellular Chemokine actin migration cytoskeleton
cytoplasmic Inositol demethylase hormone metabolism
Endocytosis Glycosphingolipid biosynthesis endoplasmic groups
Human differentiation leukemia virus infection

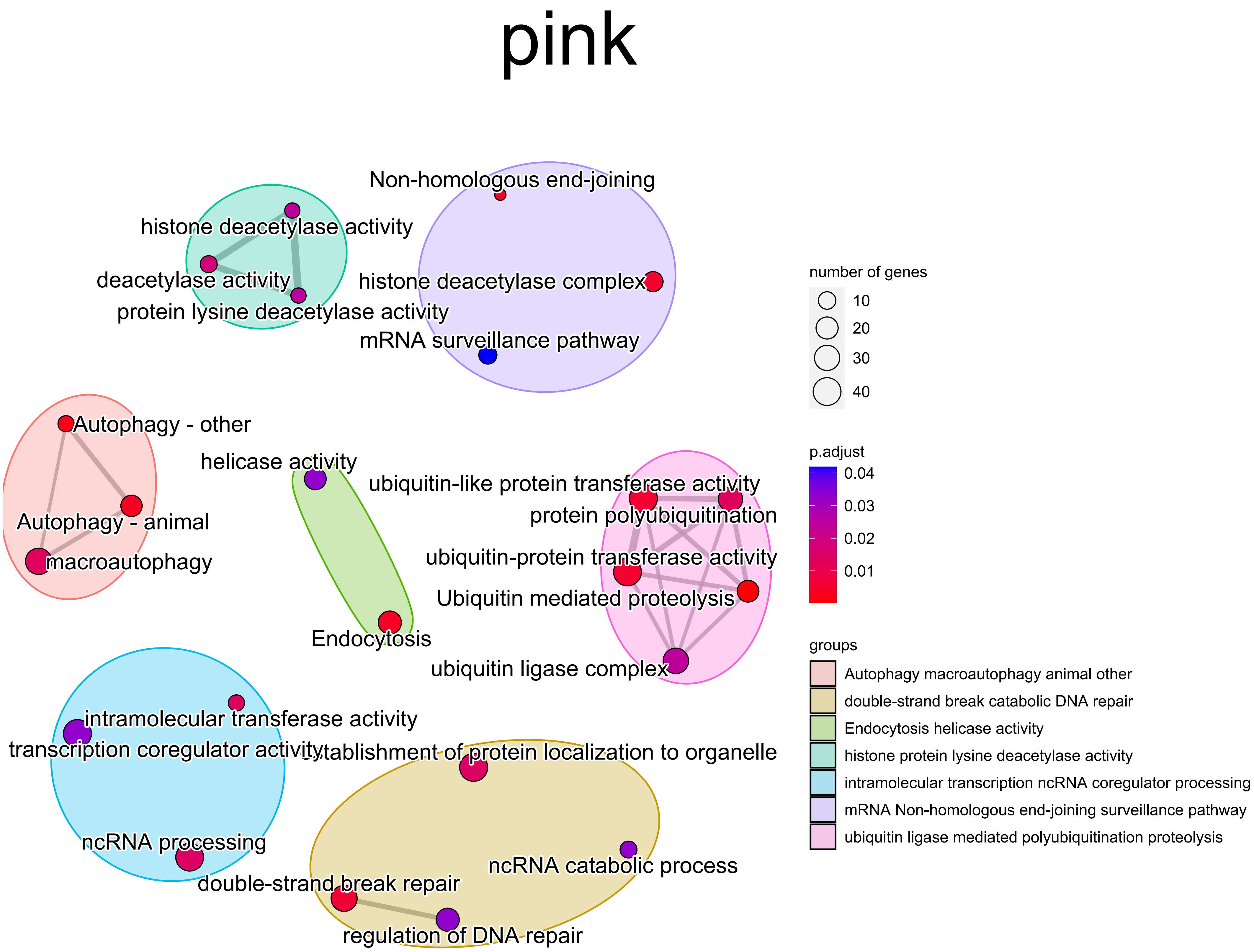

pink
Non-homologous end-joining
histone deacetylase activity
deacetylase activity
protein lysine deacetylase activity
histone deacetylase complex
mRNA surveillance pathway
Autophagy - other
helicase activity
ubiquitin-like protein transferase activity
protein polyubiquitination
Autophagy - animal
macroautophagy
ubiquitin-protein transferase activity
Ubiquitin mediated proteolysis
Endocytosis
ubiquitin ligase complex
intramolecular transferase activity
transcription coregulator activity
establishment of protein localization to organelle
ncRNA processing
ncRNA catabolic process
double-strand break repair
regulation of DNA repair
number of genes
10
20
30
40
p.adjust
0.04
0.03
0.02
0.01
groups
Autophagy macroautophagy animal other
double-strand break catabolic DNA repair
Endocytosis helicase activity
histone protein lysine deacetylase activity
intramolecular transcription ncRNA coregulator processing
mRNA Non-homologous end-joining surveillance pathway
ubiquitin ligase mediated polyubiquitination proteolysis

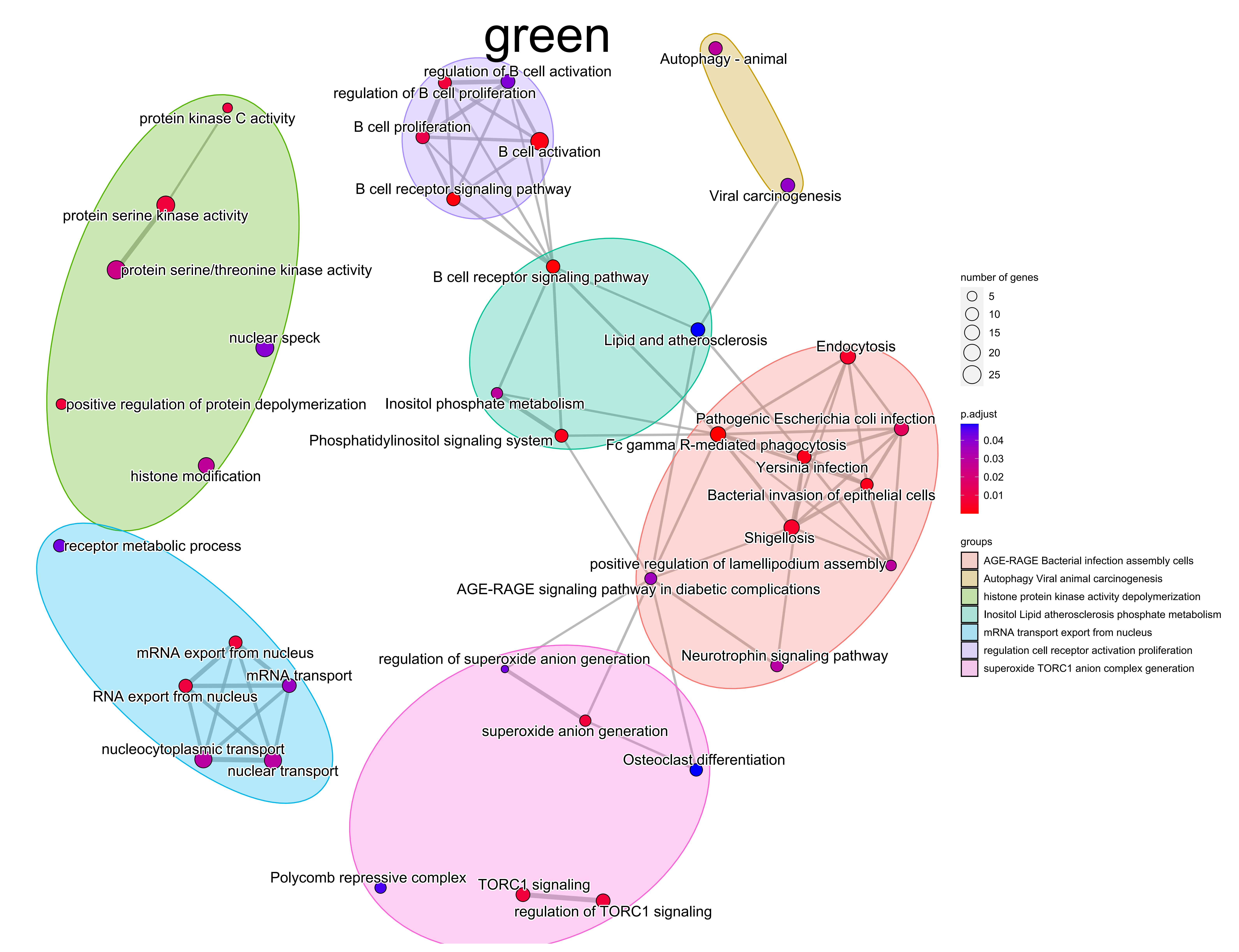
green
regulation of B cell activation
regulation of B cell proliferation
B cell proliferation
B cell activation
B cell receptor signaling pathway
Autophagy - animal
Viral carcinogenesis
protein kinase C activity
protein serine kinase activity
protein serine/threonine kinase activity
nuclear speck
positive regulation of protein depolymerization
histone modification
B cell receptor signaling pathway
Lipid and atherosclerosis
Inositol phosphate metabolism
Phosphatidylinositol signaling system
Endocytosis
Pathogenic Escherichia coli infection
Fc gamma R-mediated phagocytosis
Yersinia infection
Bacterial invasion of epithelial cells
Shigellosis
positive regulation of lamellipodium assembly
AGE-RAGE signaling pathway in diabetic complications
Neurotrophin signaling pathway
receptor metabolic process
mRNA export from nucleus
mRNA transport
RNA export from nucleus
nucleocytoplasmic transport
nuclear transport
regulation of superoxide anion generation
superoxide anion generation
Osteoclast differentiation
Polycomb repressive complex
TORC1 signaling
regulation of TORC1 signaling
number of genes
5
10
15
20
25
p.adjust
0.04
0.03
0.02
0.01
groups
AGE-RAGE Bacterial infection assembly cells
Autophagy Viral animal carcinogenesis
histone protein kinase activity depolymerization
Inositol Lipid atherosclerosis phosphate metabolism
mRNA transport export from nucleus
regulation cell receptor activation proliferation
superoxide TORC1 anion complex generation

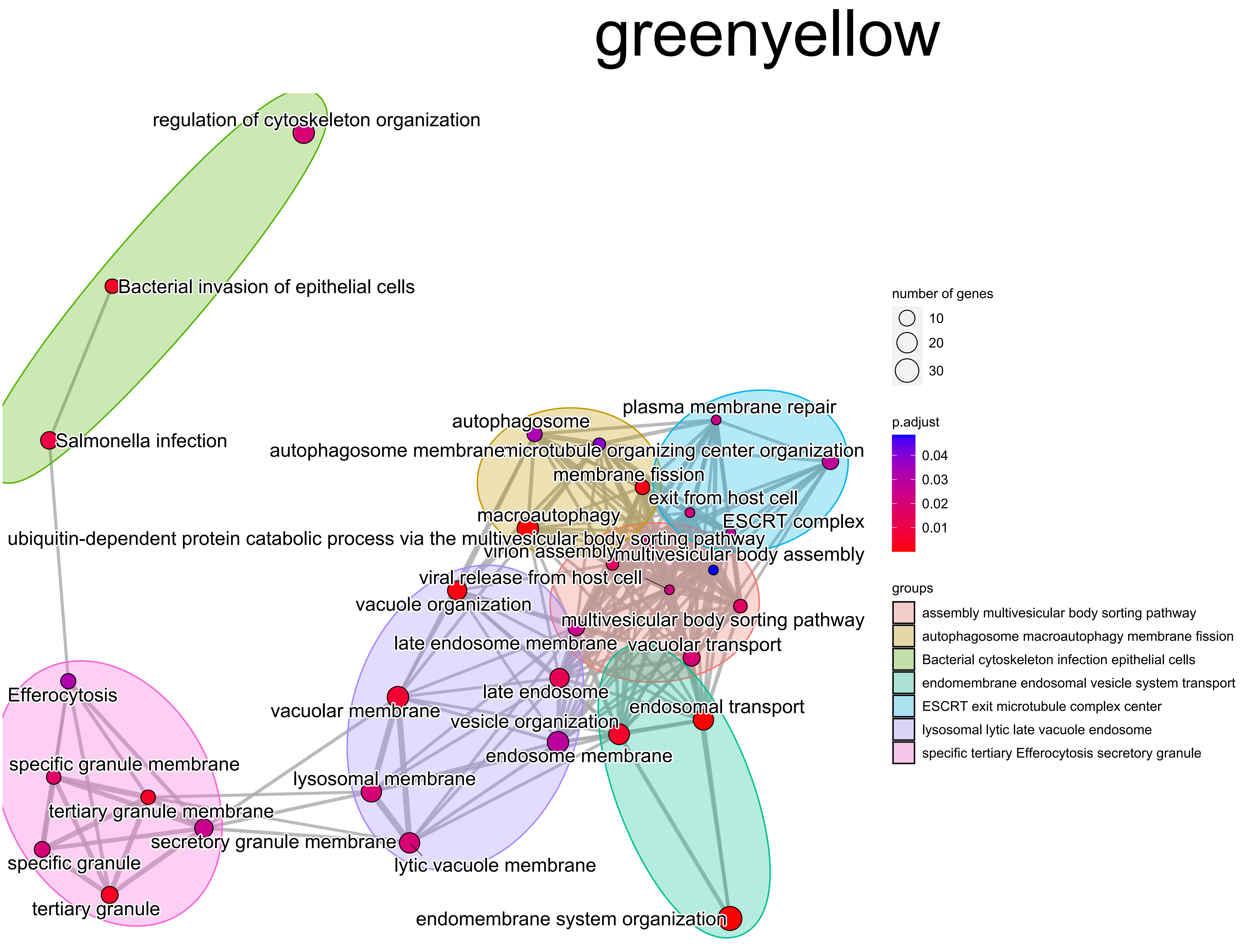

greenyellow
regulation of cytoskeleton organization
Bacterial invasion of epithelial cells
Salmonella infection
ubiquitin-dependent protein catabolic process via the multivesicular body sorting pathway
autophagosome
autophagosome membrane
microtubule organizing center organization
plasma membrane repair
membrane fission
exit from host cell
macroautophagy
ESCRT complex
virion assembly
multivesicular body assembly
viral release from host cell
vacuole organization
multivesicular body sorting pathway
late endosome membrane
vacuolar transport
late endosome
Efferocytosis
vacuolar membrane
vesicle organization
endosomal transport
endosome membrane
specific granule membrane
lysosomal membrane
tertiary granule membrane
secretory granule membrane
specific granule
lytic vacuole membrane
tertiary granule
endomembrane system organization
number of genes
10
20
30
p.adjust
0.04
0.03
0.02
0.01
groups
assembly multivesicular body sorting pathway
autophagosome macroautophagy membrane fission
Bacterial cytoskeleton infection epithelial cells
endomembrane endosomal vesicle system transport
ESCRT exit microtubule complex center
lysosomal lytic late vacuole endosome
specific tertiary Efferocytosis secretory granule

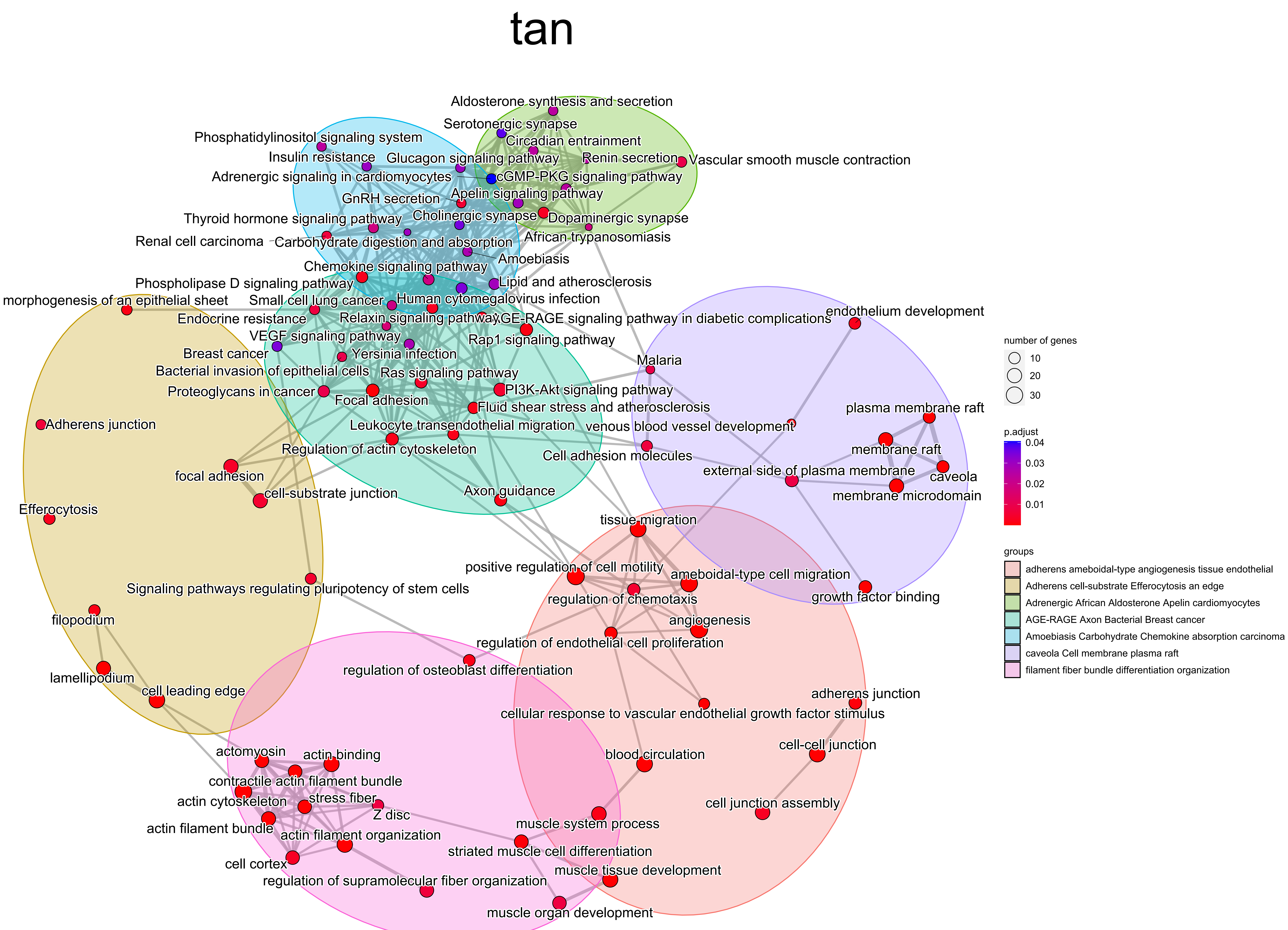

tan
Aldosterone synthesis and secretion
Serotonergic synapse
Circadian entrainment
Phosphatidylinositol signaling system
Insulin resistance
Glucagon signaling pathway
Renin secretion
Vascular smooth muscle contraction
Adrenergic signaling in cardiomyocytes
cGMP-PKG signaling pathway
GnRH secretion
Apelin signaling pathway
Thyroid hormone signaling pathway
Cholinergic synapse
Dopaminergic synapse
African trypanosomiasis
Renal cell carcinoma
Carbohydrate digestion and absorption
Amoebiasis
Chemokine signaling pathway
Phospholipase D signaling pathway
Lipid and atherosclerosis
morphogenesis of an epithelial sheet
Small cell lung cancer
Human cytomegalovirus infection
Endocrine resistance
Relaxin signaling pathway
AGE-RAGE signaling pathway in diabetic complications
endothelium development
VEGF signaling pathway
Rap1 signaling pathway
Breast cancer
Yersinia infection
Bacterial invasion of epithelial cells
Malaria
Ras signaling pathway
Proteoglycans in cancer
PI3K-Akt signaling pathway
Focal adhesion
Fluid shear stress and atherosclerosis
plasma membrane raft
Adherens junction
Leukocyte transendothelial migration
venous blood vessel development
Regulation of actin cytoskeleton
membrane raft
Cell adhesion molecules
external side of plasma membrane
caveola
focal adhesion
membrane microdomain
cell-substrate junction
Axon guidance
Efferocytosis
tissue migration
positive regulation of cell motility
ameboidal-type cell migration
Signaling pathways regulating pluripotency of stem cells
regulation of chemotaxis
growth factor binding
filopodium
angiogenesis
regulation of endothelial cell proliferation
lamellipodium
regulation of osteoblast differentiation
cell leading edge
adherens junction
cellular response to vascular endothelial growth factor stimulus
cell-cell junction
actomyosin
actin binding
blood circulation
contractile actin filament bundle
actin cytoskeleton
stress fiber
cell junction assembly
Z disc
actin filament bundle
muscle system process
actin filament organization
striated muscle cell differentiation
cell cortex
muscle tissue development
regulation of supramolecular fiber organization
muscle organ development
number of genes
10
20
30
p.adjust
0.04
0.03
0.02
0.01
groups
adherens ameboidal-type angiogenesis tissue endothelial
Adherens cell-substrate Efferocytosis an edge
Adrenergic African Aldosterone Apelin cardiomyocytes
AGE-RAGE Axon Bacterial Breast cancer
Amoebiasis Carbohydrate Chemokine absorption carcinoma
caveola Cell membrane plasma raft
filament fiber bundle differentiation organization

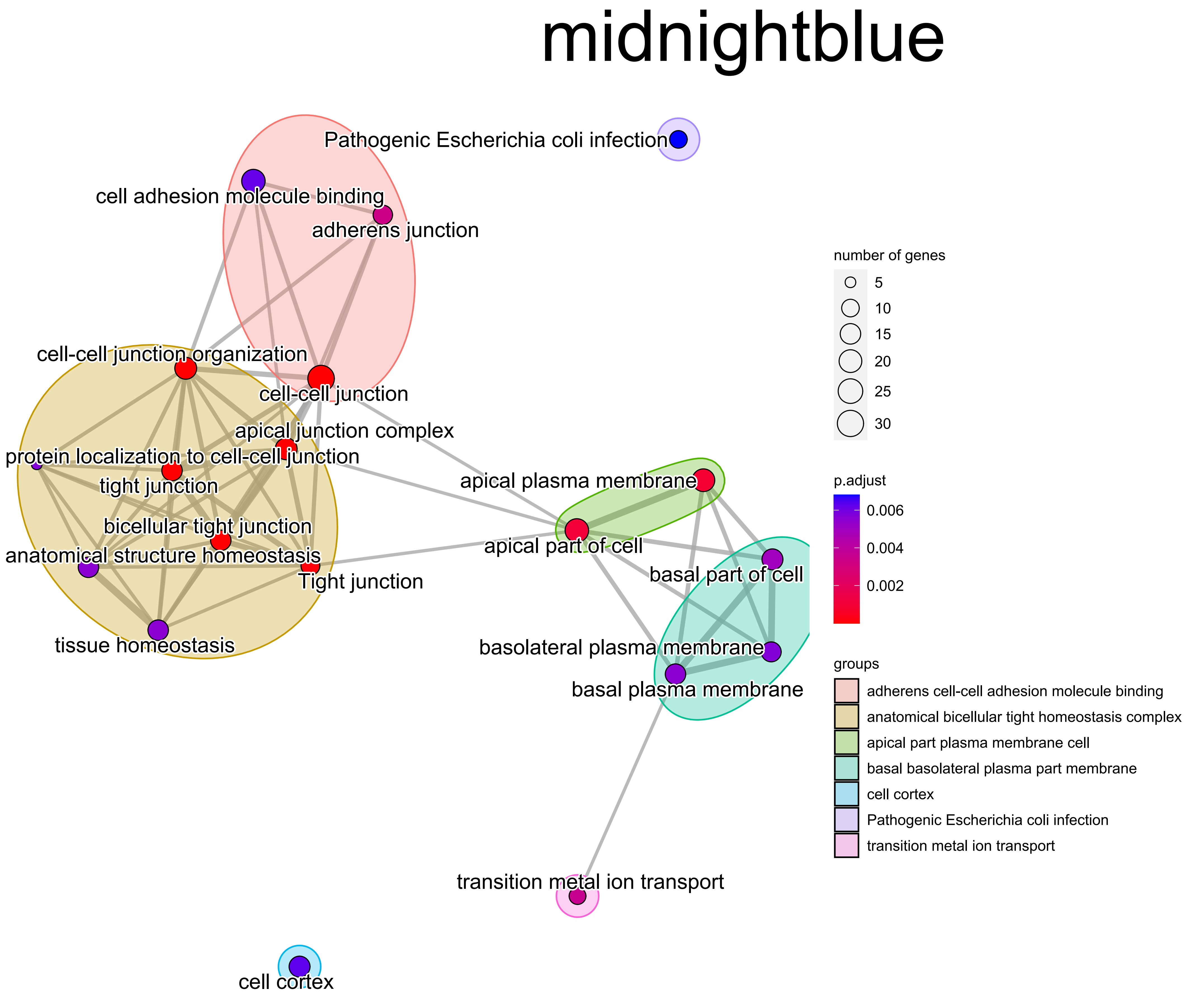
midnightblue
Pathogenic Escherichia coli infection
cell adhesion molecule binding
adherens junction
cell-cell junction organization
cell-cell junction
apical junction complex
protein localization to cell-cell junction
tight junction
bicellular tight junction
anatomical structure homeostasis
Tight junction
tissue homeostasis
apical plasma membrane
apical part of cell
basal part of cell
basolateral plasma membrane
basal plasma membrane
transition metal ion transport
cell cortex
number of genes
5
10
15
20
25
30
p.adjust
0.006
0.004
0.002
groups
adherens cell-cell adhesion molecule binding
anatomical bicellular tight homeostasis complex
apical part plasma membrane cell
basal basolateral plasma part membrane
cell cortex
Pathogenic Escherichia coli infection
transition metal ion transport

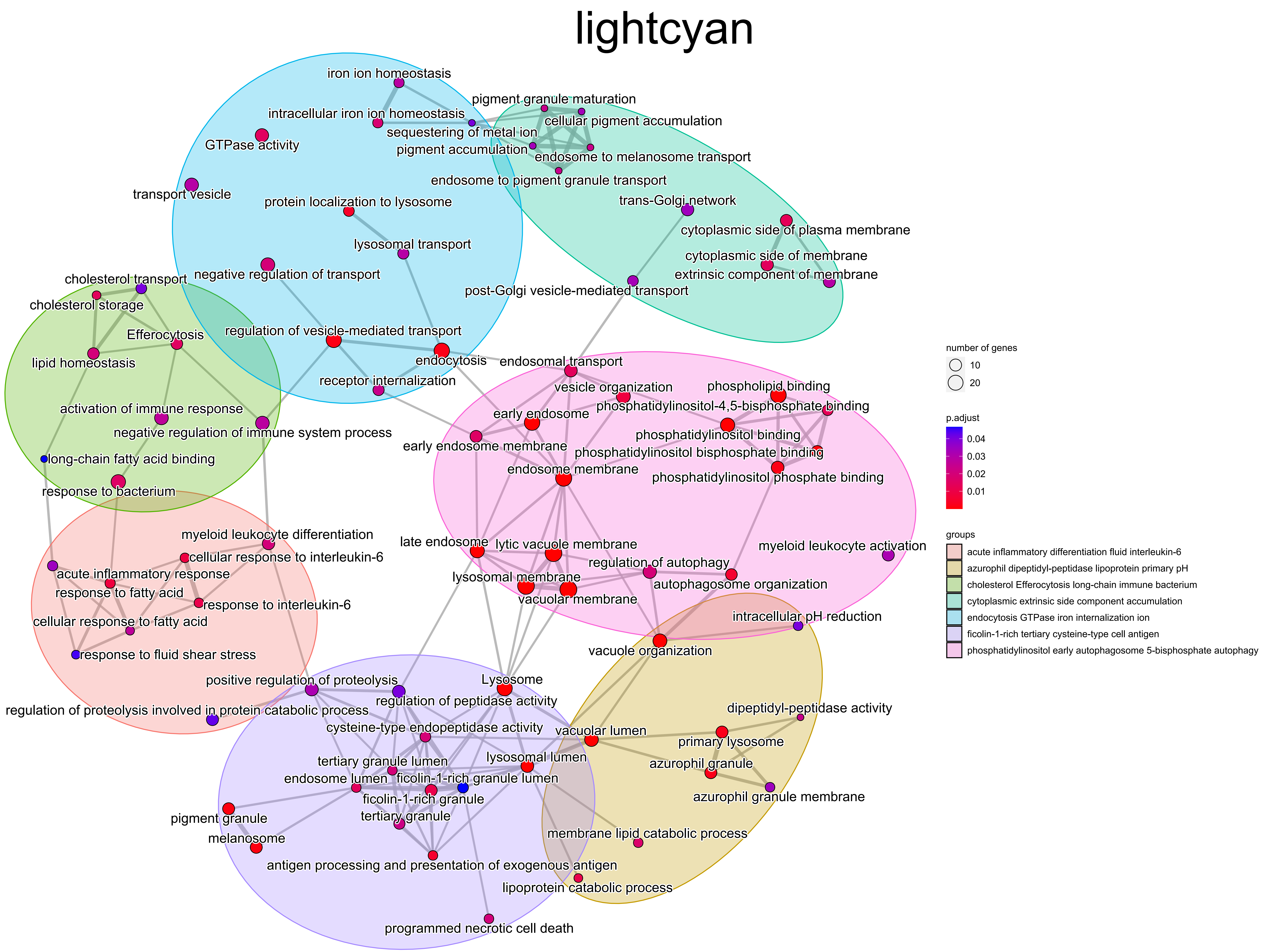
lightcyan
iron ion homeostasis
pigment granule maturation
intracellular iron ion homeostasis
cellular pigment accumulation
sequestering of metal ion
GTPase activity
pigment accumulation
endosome to melanosome transport
endosome to pigment granule transport
transport vesicle
protein localization to lysosome
trans-Golgi network
cytoplasmic side of plasma membrane
lysosomal transport
cytoplasmic side of membrane
negative regulation of transport
extrinsic component of membrane
cholesterol transport
post-Golgi vesicle-mediated transport
cholesterol storage
Efferocytosis
regulation of vesicle-mediated transport
lipid homeostasis
endocytosis
endosomal transport
receptor internalization
vesicle organization
phospholipid binding
activation of immune response
early endosome
phosphatidylinositol-4,5-bisphosphate binding
negative regulation of immune system process
phosphatidylinositol binding
early endosome membrane
phosphatidylinositol bisphosphate binding
long-chain fatty acid binding
endosome membrane
phosphatidylinositol phosphate binding
response to bacterium
myeloid leukocyte differentiation
late endosome
lytic vacuole membrane
myeloid leukocyte activation
cellular response to interleukin-6
regulation of autophagy
acute inflammatory response
lysosomal membrane
autophagosome organization
response to fatty acid
response to interleukin-6
vacuolar membrane
cellular response to fatty acid
intracellular pH reduction
vacuole organization
response to fluid shear stress
positive regulation of proteolysis
Lysosome
regulation of peptidase activity
regulation of proteolysis involved in protein catabolic process
dipeptidyl-peptidase activity
cysteine-type endopeptidase activity
vacuolar lumen
primary lysosome
tertiary granule lumen
lysosomal lumen
azurophil granule
endosome lumen
ficolin-1-rich granule lumen
azurophil granule membrane
ficolin-1-rich granule
pigment granule
tertiary granule
membrane lipid catabolic process
melanosome
antigen processing and presentation of exogenous antigen
lipoprotein catabolic process
programmed necrotic cell death
number of genes
10
20
p.adjust
0.04
0.03
0.02
0.01
groups
acute inflammatory differentiation fluid interleukin-6
azurophil dipeptidyl-peptidase lipoprotein primary pH
cholesterol Efferocytosis long-chain immune bacterium
cytoplasmic extrinsic side component accumulation
endocytosis GTPase iron internalization ion
ficolin-1-rich tertiary cysteine-type cell antigen
phosphatidylinositol early autophagosome 5-bisphosphate autophagy

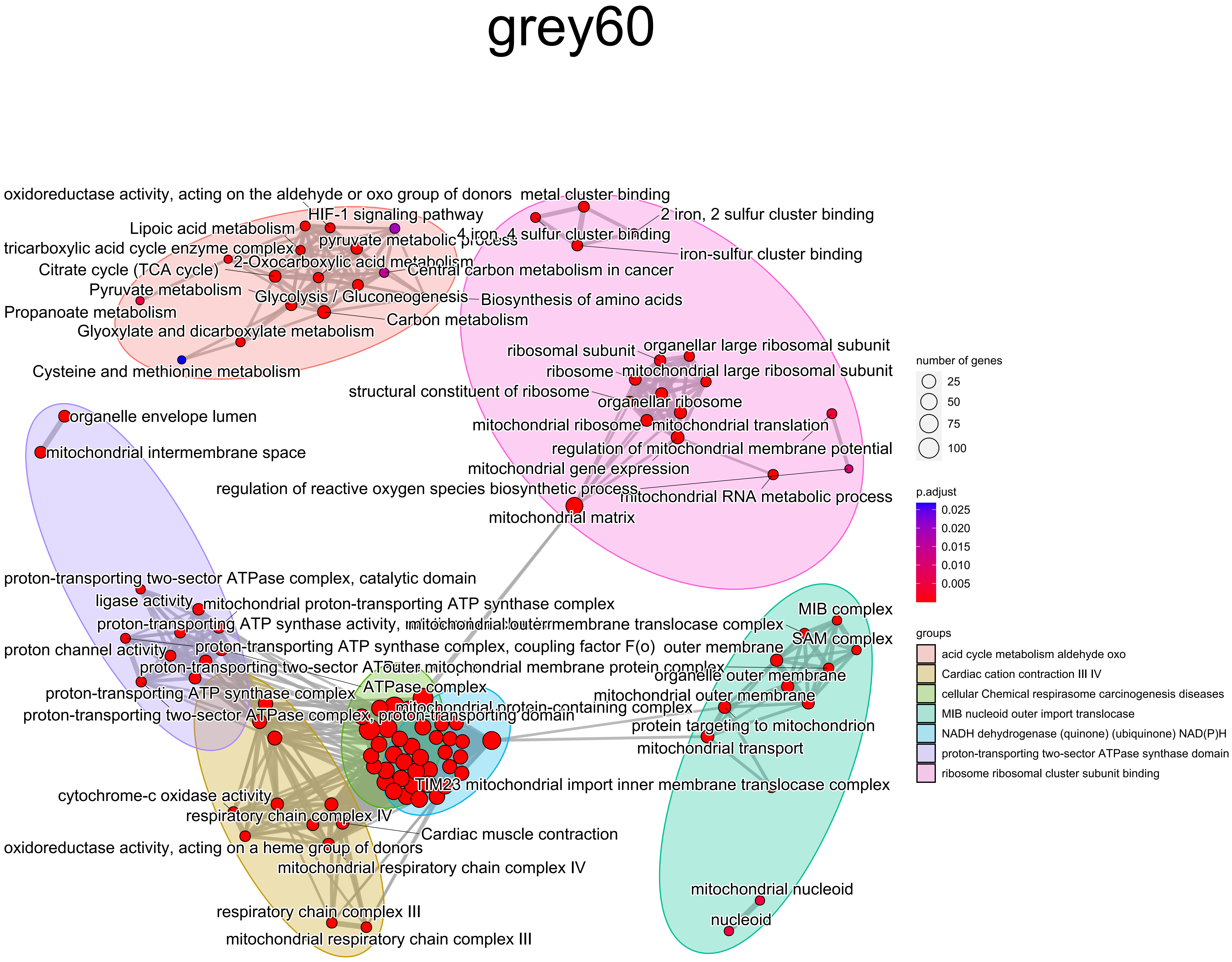

grey60
oxidoreductase activity, acting on the aldehyde or oxo group of donors
HIF-1 signaling pathway
Lipoic acid metabolism
tricarboxylic acid cycle enzyme complex
pyruvate metabolic process
Citrate cycle (TCA cycle)
2-Oxocarboxylic acid metabolism
Central carbon metabolism in cancer
Pyruvate metabolism
Glycolysis / Gluconeogenesis
Biosynthesis of amino acids
Propanoate metabolism
Carbon metabolism
Glyoxylate and dicarboxylate metabolism
Cysteine and methionine metabolism
metal cluster binding
2 iron, 2 sulfur cluster binding
4 iron, 4 sulfur cluster binding
iron-sulfur cluster binding
ribosomal subunit
organellar large ribosomal subunit
ribosome
mitochondrial large ribosomal subunit
structural constituent of ribosome
organellar ribosome
mitochondrial ribosome
mitochondrial translation
regulation of mitochondrial membrane potential
mitochondrial gene expression
regulation of reactive oxygen species biosynthetic process
mitochondrial RNA metabolic process
mitochondrial matrix
organelle envelope lumen
mitochondrial intermembrane space
proton-transporting two-sector ATPase complex, catalytic domain
ligase activity
mitochondrial proton-transporting ATP synthase complex
mitochondrial outer membrane translocase complex
proton channel activity
proton-transporting ATP synthase complex, coupling factor F(o)
outer mitochondrial membrane protein complex
ATPase complex
proton-transporting ATP synthase complex
mitochondrial protein-containing complex
proton-transporting two-sector ATPase complex, proton-transporting domain
MIB complex
SAM complex
outer membrane
organelle outer membrane
mitochondrial outer membrane
protein targeting to mitochondrion
mitochondrial transport
TIM23 mitochondrial import inner membrane translocase complex
cytochrome-c oxidase activity
respiratory chain complex IV
Cardiac muscle contraction
oxidoreductase activity, acting on a heme group of donors
mitochondrial respiratory chain complex IV
respiratory chain complex III
mitochondrial respiratory chain complex III
mitochondrial nucleoid
nucleoid
number of genes
25
50
75
100
p.adjust
0.025
0.020
0.015
0.010
0.005
groups
acid cycle metabolism aldehyde oxo
Cardiac cation contraction III IV
cellular Chemical respirasome carcinogenesis diseases
MIB nucleoid outer import translocase
NADH dehydrogenase (quinone) (ubiquinone) NAD(P)H
proton-transporting two-sector ATPase synthase domain
ribosome ribosomal cluster subunit binding

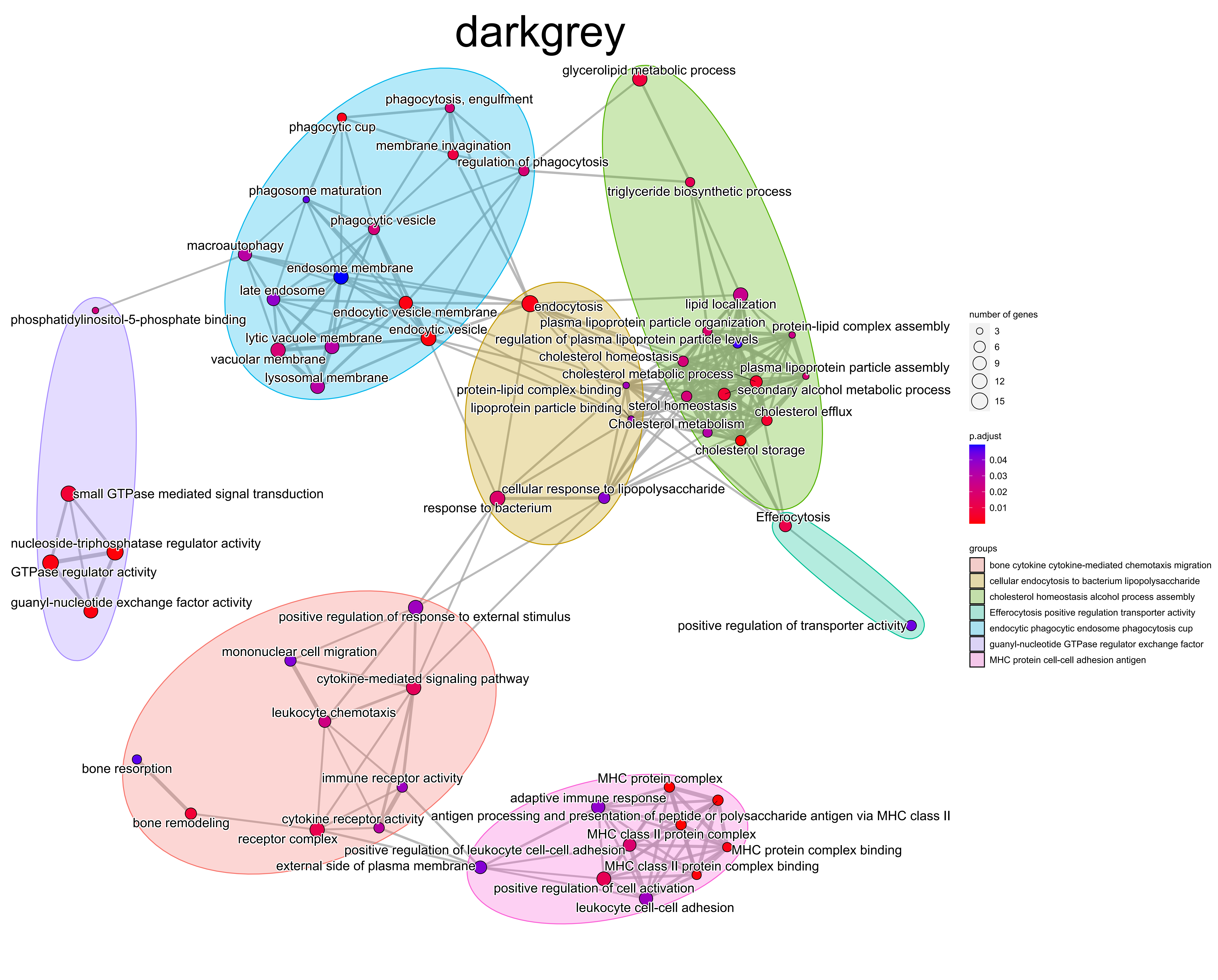
darkgrey
glycerolipid metabolic process
phagocytosis, engulfment
phagocytic cup
membrane invagination
regulation of phagocytosis
triglyceride biosynthetic process
phagosome maturation
phagocytic vesicle
macroautophagy
endosome membrane
late endosome
endocytic vesicle membrane
endocytosis
lipid localization
phosphatidylinositol-5-phosphate binding
endocytic vesicle
plasma lipoprotein particle organization
protein-lipid complex assembly
lytic vacuole membrane
regulation of plasma lipoprotein particle levels
vacuolar membrane
cholesterol homeostasis
lysosomal membrane
cholesterol metabolic process
plasma lipoprotein particle assembly
protein-lipid complex binding
secondary alcohol metabolic process
lipoprotein particle binding
sterol homeostasis
cholesterol efflux
Cholesterol metabolism
cholesterol storage
small GTPase mediated signal transduction
cellular response to lipopolysaccharide
response to bacterium
Efferocytosis
nucleoside-triphosphatase regulator activity
GTPase regulator activity
guanyl-nucleotide exchange factor activity
positive regulation of response to external stimulus
positive regulation of transporter activity
mononuclear cell migration
cytokine-mediated signaling pathway
leukocyte chemotaxis
bone resorption
immune receptor activity
MHC protein complex
adaptive immune response
antigen processing and presentation of peptide or polysaccharide antigen via MHC class II
bone remodeling
cytokine receptor activity
MHC class II protein complex
receptor complex
positive regulation of leukocyte cell-cell adhesion
MHC protein complex binding
external side of plasma membrane
MHC class II protein complex binding
positive regulation of cell activation
leukocyte cell-cell adhesion
number of genes
3
6
9
12
15
p.adjust
0.04
0.03
0.02
0.01
groups
bone cytokine cytokine-mediated chemotaxis migration
cellular endocytosis to bacterium lipopolysaccharide
cholesterol homeostasis alcohol process assembly
Efferocytosis positive regulation transporter activity
endocytic phagocytic endosome phagocytosis cup
guanyl-nucleotide GTPase regulator exchange factor
MHC protein cell-cell adhesion antigen

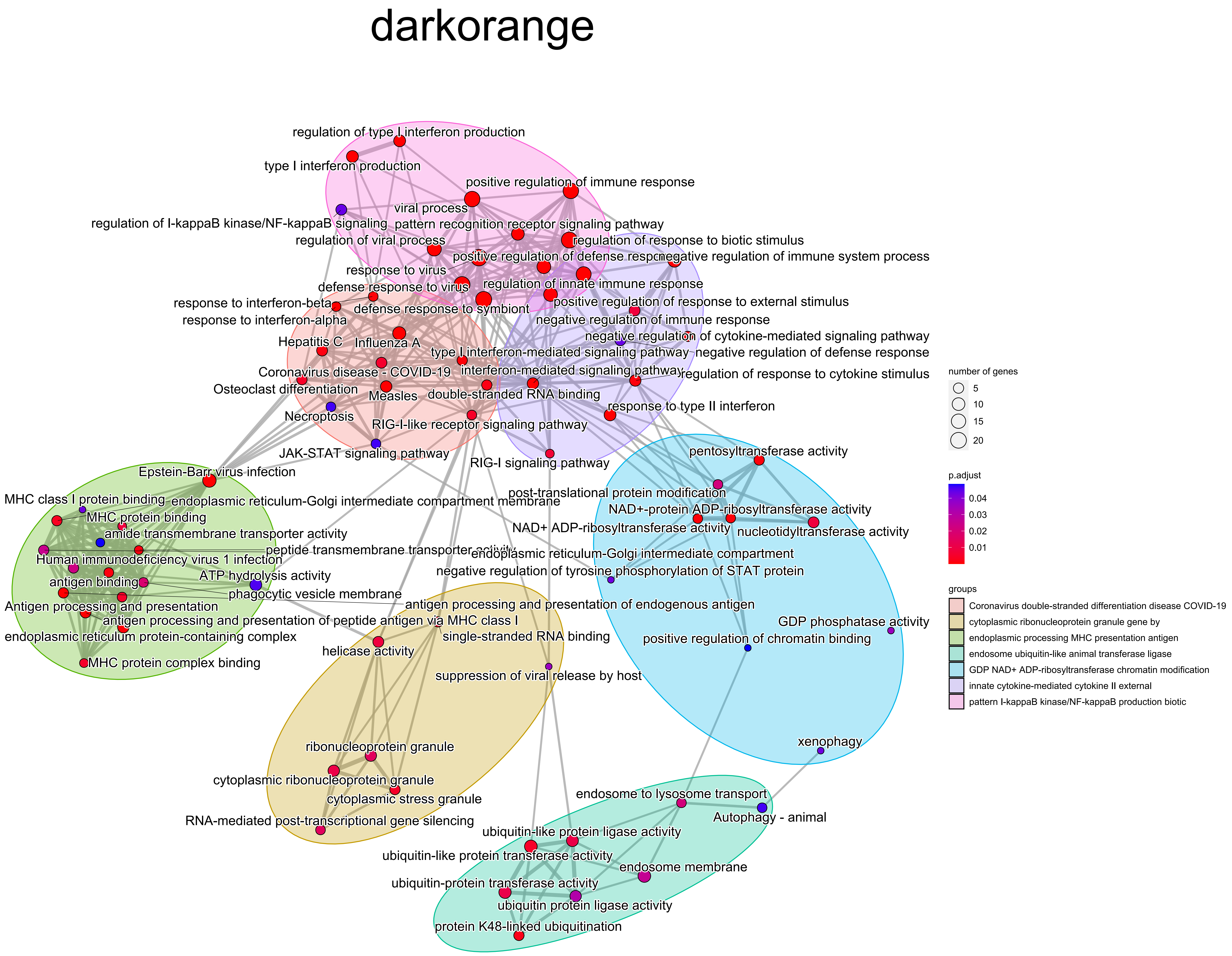
darkorange
regulation of type I interferon production
type I interferon production
positive regulation of immune response
viral process
regulation of I-kappaB kinase/NF-kappaB signaling
pattern recognition receptor signaling pathway
regulation of viral process
regulation of response to biotic stimulus
response to virus
positive regulation of defense respo
negative regulation of immune system process
defense response to virus
regulation of innate immune response
response to interferon-beta
positive regulation of response to external stimulus
response to interferon-alpha
defense response to symbiont
negative regulation of immune response
Hepatitis C
Influenza A
negative regulation of cytokine-mediated signaling pathway
type I interferon-mediated signaling pathway
negative regulation of defense response
Coronavirus disease - COVID-19
interferon-mediated signaling pathway
regulation of response to cytokine stimulus
Osteoclast differentiation
Measles
double-stranded RNA binding
Necroptosis
response to type II interferon
RIG-I-like receptor signaling pathway
JAK-STAT signaling pathway
RIG-I signaling pathway
pentosyltransferase activity
Epstein-Barr virus infection
MHC class I protein binding
endoplasmic reticulum-Golgi intermediate compartment membrane
post-translational protein modification
MHC protein binding
NAD+-protein ADP-ribosyltransferase activity
amide transmembrane transporter activity
NAD+ ADP-ribosyltransferase activity
nucleotidyltransferase activity
peptide transmembrane transporter activity
Human immunodeficiency virus 1 infection
endoplasmic reticulum-Golgi intermediate compartment
ATP hydrolysis activity
negative regulation of tyrosine phosphorylation of STAT protein
antigen binding
phagocytic vesicle membrane
antigen processing and presentation of endogenous antigen
Antigen processing and presentation
antigen processing and presentation of peptide antigen via MHC class I
GDP phosphatase activity
endoplasmic reticulum protein-containing complex
single-stranded RNA binding
positive regulation of chromatin binding
helicase activity
MHC protein complex binding
suppression of viral release by host
ribonucleoprotein granule
xenophagy
cytoplasmic ribonucleoprotein granule
cytoplasmic stress granule
endosome to lysosome transport
RNA-mediated post-transcriptional gene silencing
Autophagy - animal
ubiquitin-like protein ligase activity
ubiquitin-like protein transferase activity
endosome membrane
ubiquitin-protein transferase activity
ubiquitin protein ligase activity
protein K48-linked ubiquitination
number of genes
5
10
15
20
p.adjust
0.04
0.03
0.02
0.01
groups
Coronavirus double-stranded differentiation disease COVID-19
cytoplasmic ribonucleoprotein granule gene by
endoplasmic processing MHC presentation antigen
endosome ubiquitin-like animal transferase ligase
GDP NAD+ ADP-ribosyltransferase chromatin modification
innate cytokine-mediated cytokine II external
pattern I-kappaB kinase/NF-kappaB production biotic

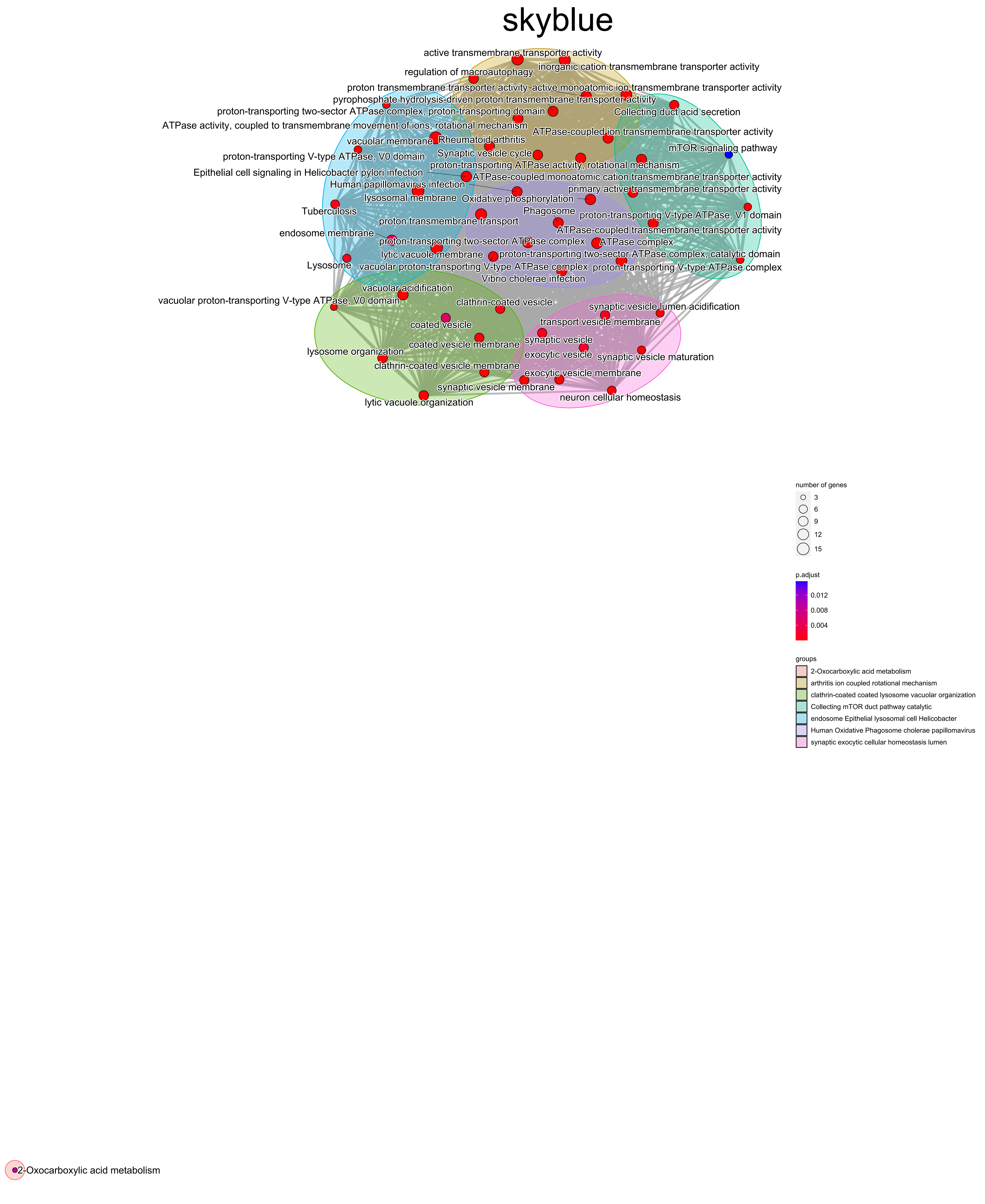

skyblue
active transmembrane transporter activity
inorganic cation transmembrane transporter activity
regulation of macroautophagy
proton transmembrane transporter activity
active monoatomic ion transmembrane transporter activity
pyrophosphate hydrolysis-driven proton transmembrane transporter activity
proton-transporting two-sector ATPase complex, proton-transporting domain
Collecting duct acid secretion
ATPase activity, coupled to transmembrane movement of ions, rotational mechanism
ATPase-coupled ion transmembrane transporter activity
vacuolar membrane
Rheumatoid arthritis
mTOR signaling pathway
proton-transporting V-type ATPase, V0 domain
Synaptic vesicle cycle
proton-transporting ATPase activity, rotational mechanism
Epithelial cell signaling in Helicobacter pylori infection
ATPase-coupled monoatomic cation transmembrane transporter activity
Human papillomavirus infection
primary active transmembrane transporter activity
lysosomal membrane
Oxidative phosphorylation
Tuberculosis
Phagosome
proton-transporting V-type ATPase, V1 domain
proton transmembrane transport
ATPase-coupled transmembrane transporter activity
endosome membrane
proton-transporting two-sector ATPase complex
ATPase complex
lytic vacuole membrane
proton-transporting two-sector ATPase complex, catalytic domain
Lysosome
vacuolar proton-transporting V-type ATPase complex
proton-transporting V-type ATPase complex
Vibrio cholerae infection
vacuolar acidification
vacuolar proton-transporting V-type ATPase, V0 domain
clathrin-coated vesicle
synaptic vesicle lumen acidification
coated vesicle
transport vesicle membrane
coated vesicle membrane
synaptic vesicle
lysosome organization
exocytic vesicle
synaptic vesicle maturation
clathrin-coated vesicle membrane
exocytic vesicle membrane
synaptic vesicle membrane
neuron cellular homeostasis
lytic vacuole organization
number of genes
3
6
9
12
15
p.adjust
0.012
0.008
0.004
groups
2-Oxocarboxylic acid metabolism
arthritis ion coupled rotational mechanism
clathrin-coated coated lysosome vacuolar organization
Collecting mTOR duct pathway catalytic
endosome Epithelial lysosomal cell Helicobacter
Human Oxidative Phagosome cholerae papillomavirus
synaptic exocytic cellular homeostasis lumen
2-Oxocarboxylic acid metabolism

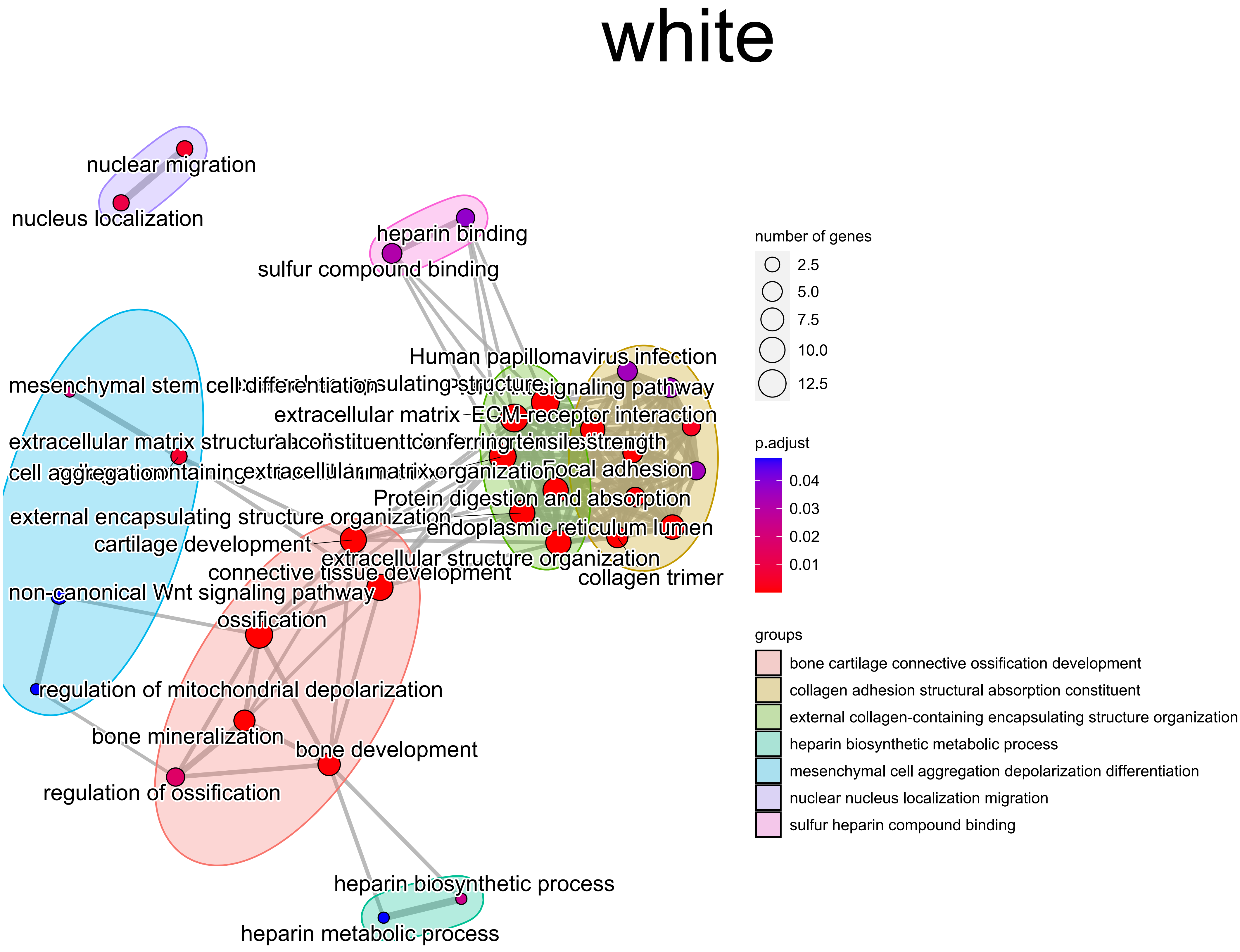
white
nuclear migration
nucleus localization
heparin binding
sulfur compound binding
Human papillomavirus infection
mesenchymal stem cell differentiation
signaling pathway
extracellular matrix
ECM-receptor interaction
Focal adhesion
Protein digestion and absorption
external encapsulating structure organization
endoplasmic reticulum lumen
cartilage development
extracellular structure organization
connective tissue development
collagen trimer
non-canonical Wnt signaling pathway
ossification
regulation of mitochondrial depolarization
bone mineralization
bone development
regulation of ossification
heparin biosynthetic process
heparin metabolic process
number of genes
2.5
5.0
7.5
10.0
12.5
p.adjust
0.04
0.03
0.02
0.01
groups
bone cartilage connective ossification development
collagen adhesion structural absorption constituent
external collagen-containing encapsulating structure organization
heparin biosynthetic metabolic process
mesenchymal cell aggregation depolarization differentiation
nuclear nucleus localization migration
sulfur heparin compound binding

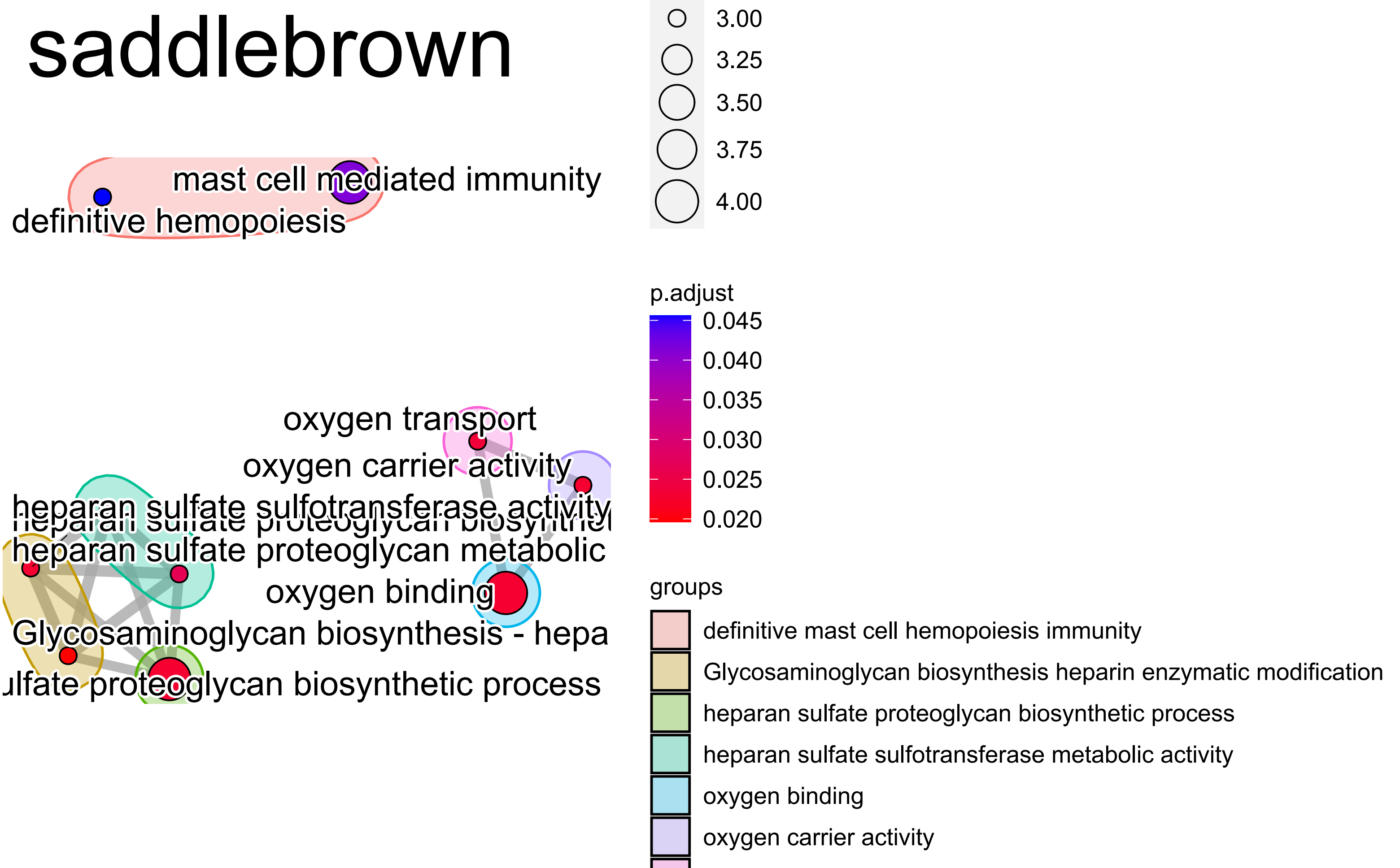
saddlebrown
mast cell mediated immunity
definitive hemopoiesis
oxygen transport
oxygen carrier activity
heparan sulfate sulfotransferase activity
heparan sulfate proteoglycan metabolic
oxygen binding
Glycosaminoglycan biosynthesis - hepa
ulfate proteoglycan biosynthetic process
3.00
3.25
3.50
3.75
4.00
p.adjust
0.045
0.040
0.035
0.030
0.025
0.020
groups
definitive mast cell hemopoiesis immunity
Glycosaminoglycan biosynthesis heparin enzymatic modification
heparan sulfate proteoglycan biosynthetic process
heparan sulfate sulfotransferase metabolic activity
oxygen binding
oxygen carrier activity

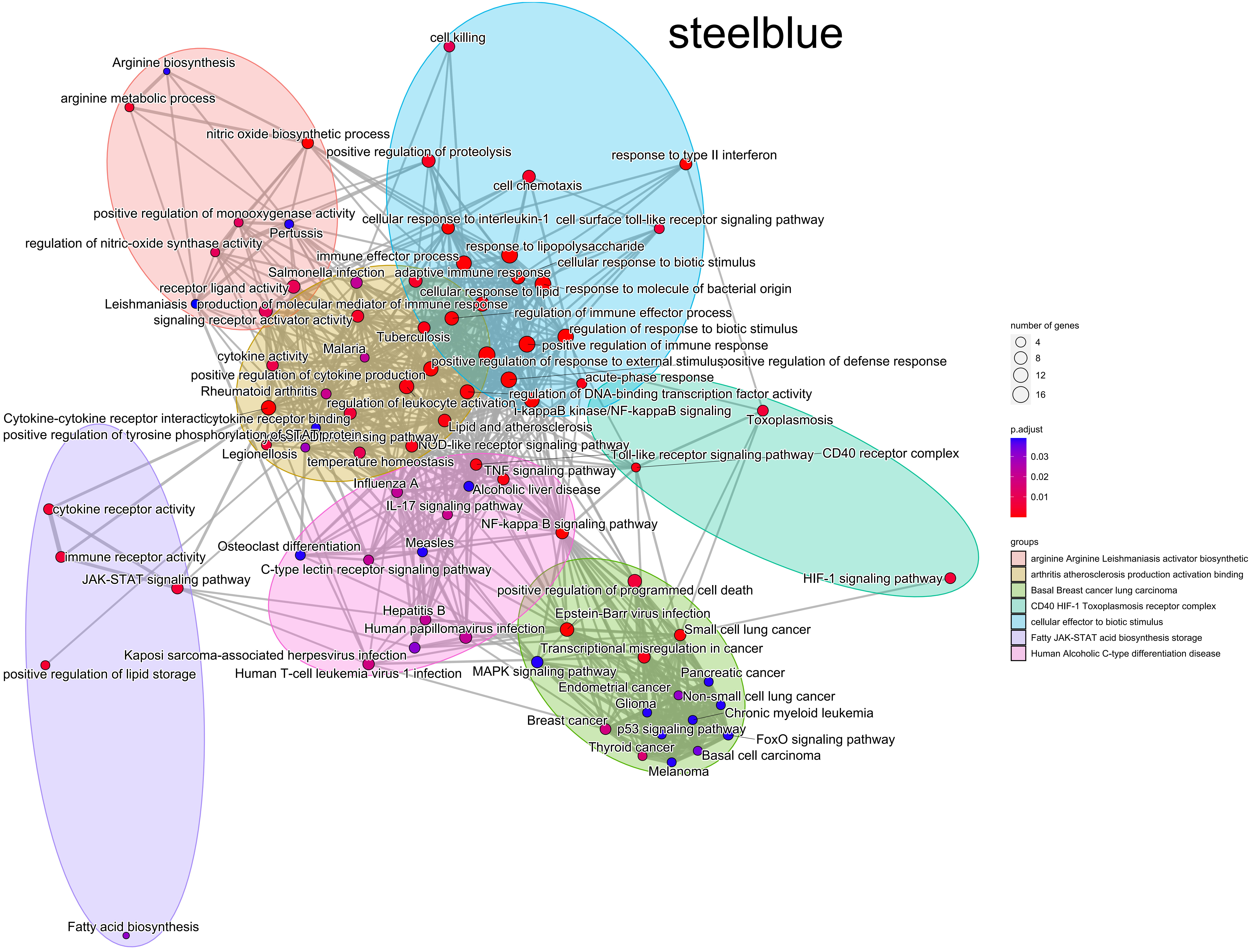
steelblue
cell killing
Arginine biosynthesis
arginine metabolic process
nitric oxide biosynthetic process
positive regulation of proteolysis
response to type II interferon
cell chemotaxis
positive regulation of monooxygenase activity
Pertussis
cellular response to interleukin-1
cell surface toll-like receptor signaling pathway
regulation of nitric-oxide synthase activity
response to lipopolysaccharide
immune effector process
cellular response to biotic stimulus
Salmonella infection
adaptive immune response
receptor ligand activity
cellular response to lipid
response to molecule of bacterial origin
Leishmaniasis
production of molecular mediator of immune response
signaling receptor activator activity
regulation of immune effector process
Tuberculosis
regulation of response to biotic stimulus
positive regulation of immune response
Malaria
cytokine activity
positive regulation of response to external stimulus
positive regulation of defense response
positive regulation of cytokine production
acute-phase response
Rheumatoid arthritis
regulation of DNA-binding transcription factor activity
regulation of leukocyte activation
I-kappaB kinase/NF-kappaB signaling
Toxoplasmosis
cytokine receptor binding
Lipid and atherosclerosis
positive regulation of tyrosine phosphorylation of STAT protein
NOD-like receptor signaling pathway
Legionellosis
Toll-like receptor signaling pathway
CD40 receptor complex
temperature homeostasis
TNF signaling pathway
Influenza A
Alcoholic liver disease
IL-17 signaling pathway
cytokine receptor activity
NF-kappa B signaling pathway
Osteoclast differentiation
Measles
immune receptor activity
C-type lectin receptor signaling pathway
JAK-STAT signaling pathway
HIF-1 signaling pathway
positive regulation of programmed cell death
Hepatitis B
Epstein-Barr virus infection
Human papillomavirus infection
Small cell lung cancer
Kaposi sarcoma-associated herpesvirus infection
Transcriptional misregulation in cancer
positive regulation of lipid storage
Human T-cell leukemia virus 1 infection
MAPK signaling pathway
Pancreatic cancer
Endometrial cancer
Non-small cell lung cancer
Glioma
Chronic myeloid leukemia
Breast cancer
p53 signaling pathway
FoxO signaling pathway
Thyroid cancer
Basal cell carcinoma
Melanoma
Fatty acid biosynthesis
number of genes
4
8
12
16
p.adjust
0.03
0.02
0.01
groups
arginine Arginine Leishmaniasis activator biosynthetic
arthritis atherosclerosis production activation binding
Basal Breast cancer lung carcinoma
CD40 HIF-1 Toxoplasmosis receptor complex
cellular effector to biotic stimulus
Fatty JAK-STAT acid biosynthesis storage
Human Alcoholic C-type differentiation disease

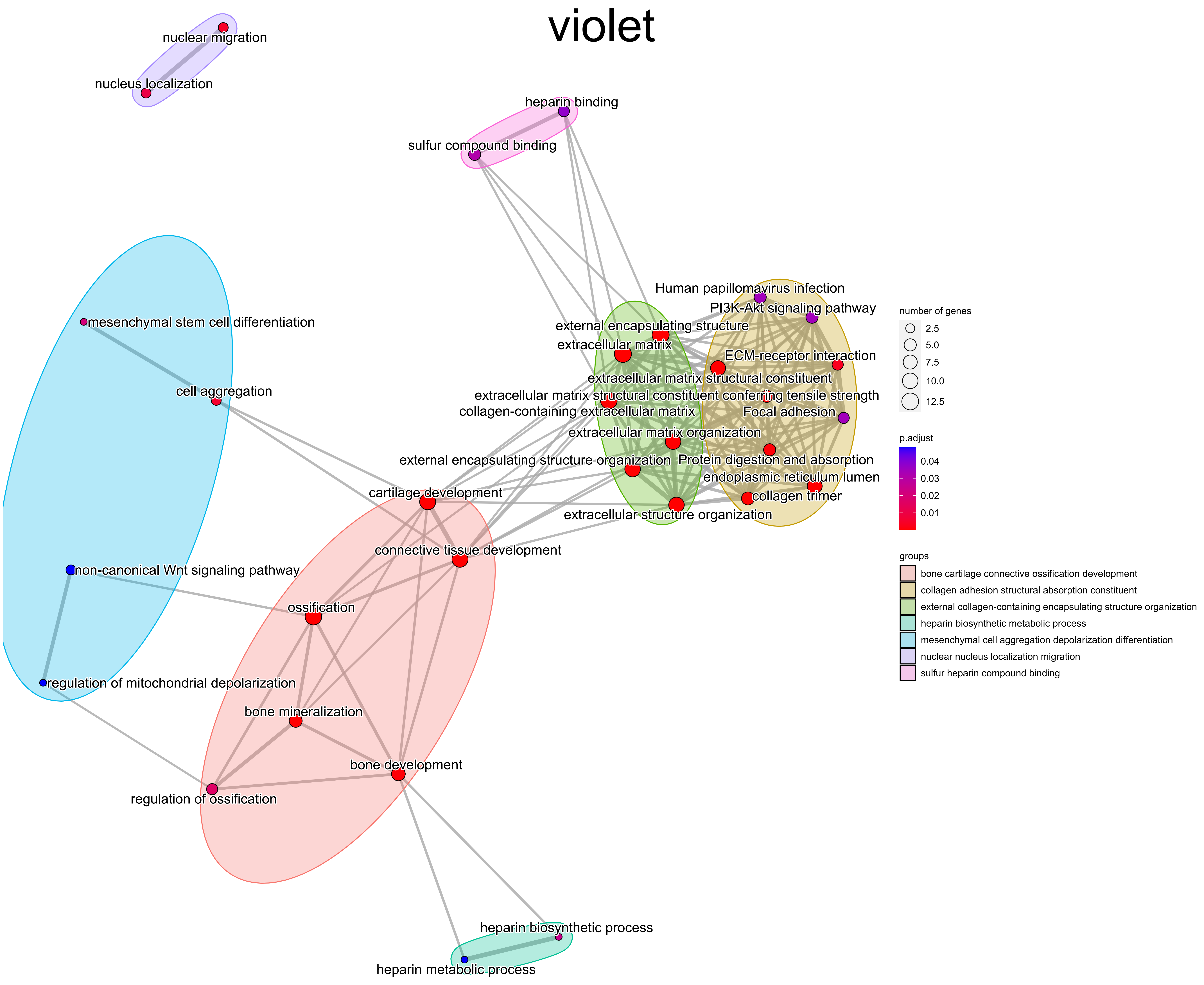
violet
nuclear migration
nucleus localization
heparin binding
sulfur compound binding
Human papillomavirus infection
PI3K-Akt signaling pathway
external encapsulating structure
extracellular matrix
ECM-receptor interaction
extracellular matrix structural constituent
extracellular matrix structural constituent conferring tensile strength
collagen-containing extracellular matrix
Focal adhesion
extracellular matrix organization
external encapsulating structure organization
Protein digestion and absorption
endoplasmic reticulum lumen
collagen trimer
extracellular structure organization
mesenchymal stem cell differentiation
cell aggregation
cartilage development
connective tissue development
non-canonical Wnt signaling pathway
ossification
regulation of mitochondrial depolarization
bone mineralization
bone development
regulation of ossification
heparin biosynthetic process
heparin metabolic process
number of genes
2.5
5.0
7.5
10.0
12.5
p.adjust
0.04
0.03
0.02
0.01
groups
bone cartilage connective ossification development
collagen adhesion structural absorption constituent
external collagen-containing encapsulating structure organization
heparin biosynthetic metabolic process
mesenchymal cell aggregation depolarization differentiation
nuclear nucleus localization migration
sulfur heparin compound binding
