# Supplementary material for: Deciphering infected cell types, hub gene networks and cell-cell communication in infectious bronchitis virus via single-cell RNA sequencing
Source: PLoS Pathog. 2024 May 14;20(5):e1012232. doi: 10.1371/journal.ppat.1012232 (PMC11125504; doi:10.1371/journal.ppat.1012232)
Supplement: S14 Fig — (PDF) [file ppat.1012232.s014.pdf]

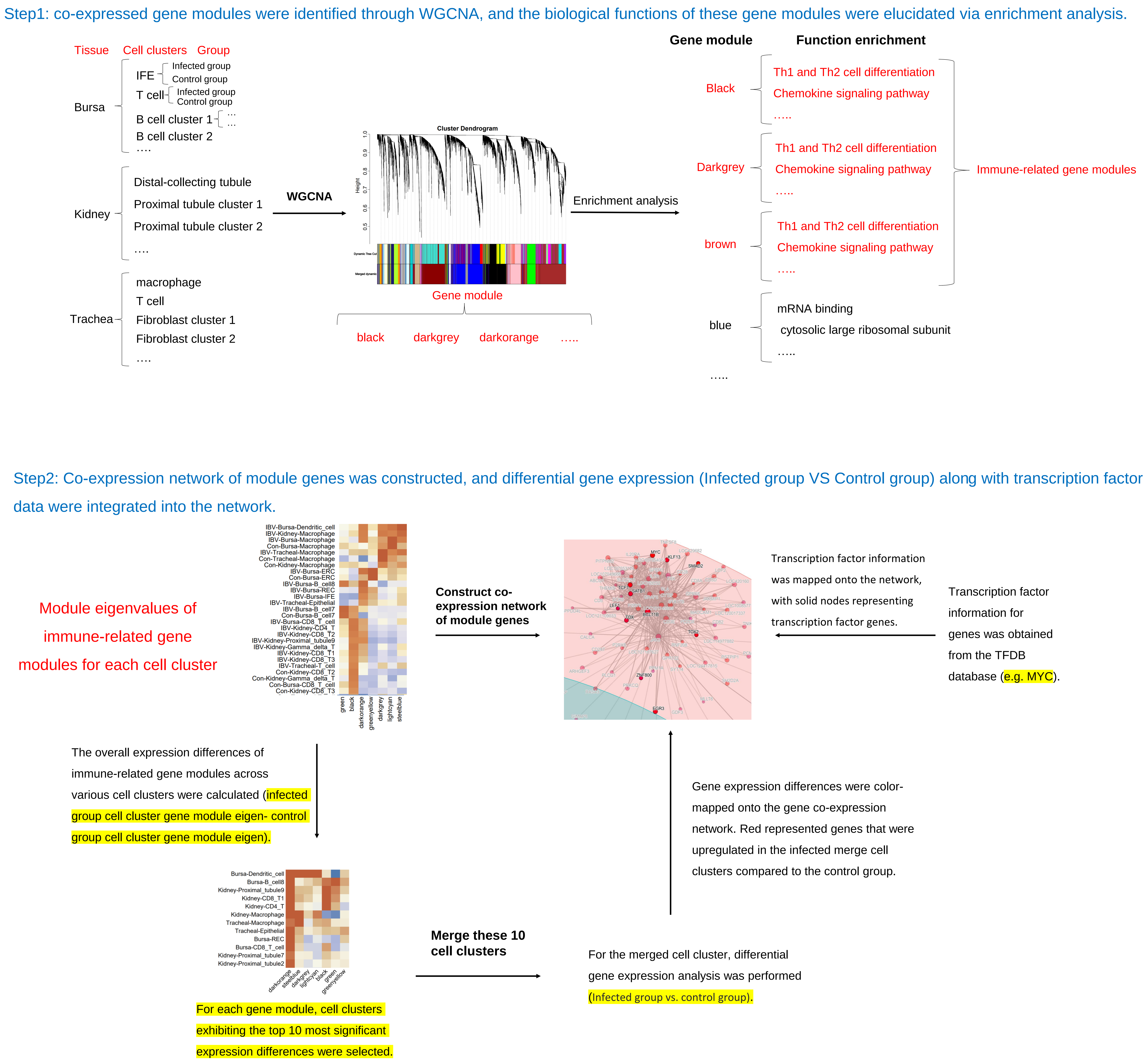
Step1: co-expressed gene modules were identified through WGCNA, and the biological functions of these gene modules were elucidated via enrichment analysis.
Tissue
Cell clusters
Group
Bursa
IFE
Infected group
Control group
T cell
Infected group
Control group
B cell cluster 1
B cell cluster 2
....
Kidney
Distal-collecting tubule
Proximal tubule cluster 1
Proximal tubule cluster 2
....
Trachea
macrophage
T cell
Fibroblast cluster 1
Fibroblast cluster 2
....
WGCNA
Cluster Dendrogram
Height
Gene module
black
darkgrey
darkorange
.....
Enrichment analysis
Gene module
Function enrichment
Black
Th1 and Th2 cell differentiation
Chemokine signaling pathway
.....
Darkgrey
Th1 and Th2 cell differentiation
Chemokine signaling pathway
.....
brown
Th1 and Th2 cell differentiation
Chemokine signaling pathway
.....
Immune-related gene modules
blue
mRNA binding
cytosolic large ribosomal subunit
.....
.....
Step2: Co-expression network of module genes was constructed, and differential gene expression (Infected group VS Control group) along with transcription factor data were integrated into the network.
Module eigenvalues of immune-related gene modules for each cell cluster
IBV-Bursa-Dendritic_cell
IBV-Kidney-Macrophage
IBV-Bursa-Macrophage
Con-Bursa-Macrophage
IBV-Tracheal-Macrophage
Con-Tracheal-Macrophage
Con-Kidney-Macrophage
IBV-Bursa-ERC
Con-Bursa-ERC
IBV-Bursa-B_cell8
IBV-Bursa-REC
IBV-Bursa-IFE
IBV-Tracheal-Epithelial
IBV-Bursa-B_cell7
Con-Bursa-B_cell7
IBV-Bursa-CD8_T_cell
IBV-Kidney-CD4_T
IBV-Kidney-CD8_T2
IBV-Kidney-Proximal_tubule9
IBV-Kidney-Gamma_delta_T
IBV-Kidney-CD8_T1
IBV-Kidney-CD8_T3
IBV-Tracheal-T_cell
Con-Kidney-CD8_T2
Con-Kidney-Gamma_delta_T
Con-Bursa-CD8_T_cell
Con-Kidney-CD8_T3
green
black
darkorange
greenyellow
darkgrey
lightcyan
steelblue
Construct co-expression network of module genes
Transcription factor information was mapped onto the network, with solid nodes representing transcription factor genes.
Transcription factor information for genes was obtained from the TFDB database (e.g. MYC).
The overall expression differences of immune-related gene modules across various cell clusters were calculated (infected group cell cluster gene module eigen- control group cell cluster gene module eigen).
Gene expression differences were color-mapped onto the gene co-expression network. Red represented genes that were upregulated in the infected merge cell clusters compared to the control group.
Bursa-Dendritic_cell
Bursa-B_cell8
Kidney-Proximal_tubule9
Kidney-CD8_T1
Kidney-CD4_T
Kidney-Macrophage
Tracheal-Macrophage
Tracheal-Epithelial
Bursa-REC
Bursa-CD8_T_cell
Kidney-Proximal_tubule7
Kidney-Proximal_tubule2
darkorange
steelblue
darkgrey
lightcyan
black
green
greenyellow
For each gene module, cell clusters exhibiting the top 10 most significant expression differences were selected.
Merge these 10 cell clusters
For the merged cell cluster, differential gene expression analysis was performed (Infected group vs. control group).
