# Supplementary material for: Deciphering infected cell types, hub gene networks and cell-cell communication in infectious bronchitis virus via single-cell RNA sequencing
Source: PLoS Pathog. 2024 May 14;20(5):e1012232. doi: 10.1371/journal.ppat.1012232 (PMC11125504; doi:10.1371/journal.ppat.1012232)
Supplement: Attachment — Submitted filename: Response letter.docx [file ppat.1012232.s033.docx]

**Response to Reviewer’s Comment:** Manuscript ID PPATHOGENS-D-23-02210

**Manuscript Title:** Deciphering Infected Cell Types, Hub Gene Networks and Cell-Cell Communication in Infectious Bronchitis Virus via Single-Cell RNA Sequencing

**Part I - Summary**

Please use this section to discuss strengths/weaknesses of study, novelty/significance, general execution and scholarship.

**Reviewer #1:** In this manuscript, Liukang et al describe gene expression in chickens infected with infectious bronchitis virus. IBV is understudied adding to the significance of this submission. This manuscript uses single cell sequencing to describe gene expression in the organs from infected animals. The study is comprehensive and the data are discussed in good detail. It is however, largely a descriptive study lacking mechanistic insight. It would be appropriate to consider the manuscript as a Resource article. Specific comments are below.

**Reviewer #2:** Liukang et al. present a detailed analysis employing immunohistochemistry and scRNA-seq to evaluate the target cells and inflammatory responses induced by IBV in chickens. The study has novelty in investigating a coronavirus of high importance to the poultry industry, and in the approaches employed, including detailed RNA-seq analyses of the Bursa of Fabricus and the chicken kidney which are novel. The study identifies unique target cells of infection in both tissues, though much was already known about infection of tracheal ciliated cells (not seen here) and infection renal tubular epithelial cells. The study identified acute inflammation in the distal nephrons with evidence for disrupted water-salt balance which could contribute to renal urate accumulation and mortality. Communication analysis also confirmed inflammatory signaling from infected cells in the distal nephron to immune cells. Overall this is a fairly thorough characterization of viral tropisms and the host response. The therapeutic implications for poultry are unclear, but this does represent an important piece of understanding animal and human health. However, there are some concerns, particularly with the analyses of infected cells, that limit the ability to draw biologically significant or novel conclusions. These concerns about that analytic approach significantly detract from this paper. Another limitation is that not all tissues were sampled, so an important host response could have been missed, even if it was not directly induced by infection.

**Part II – Major Issues: Key Experiments Required for Acceptance**

**Reviewer #2:**

Thank you for your comments and valuable insights to improve the quality of our manuscript. The detailed corrections are listed below.

*1. It is unclear how the timepoint of 5 days post-infection was chosen, and somewhat unfortunate in that they were unable to detect any infection in the tracheal epithelium—presumably the first site of infection. Certainly a variety of time points may not be feasible but some justification and understanding would be helpful to orient the reader.*

**Response:** Thank you for your valuable comment. The sampling timepoint of 5 days post-infection (dpi) for 1-day-old SPF chickens was selected because IBV initially replicates in the trachea before disseminating to the kidneys and Fabricius bursa. Since IBV causes a transient infection in the trachea, an excessively early sampling time would preclude virus detection in the kidneys and bursa, whereas a delayed timepoint would result in viral clearance from the trachea. At 5 dpi, the trachea, kidneys and bursa are concurrently infected, enabling single-cell sequencing analysis from the same infected bird, ensuring data consistency. While some infected tracheal cells had been cleared by this stage, we indeed detected virus-infected cells in the trachea. Although not prominently visible in Fig. 1b, we re-performed immunofluorescence assays on archived paraffin-embedded tissues and detected viral antigen, as illustrated in the revised Fig. 1b. Viral infections were also detected in the single-cell sequencing data (S4 Figure 1 and Table 1-3). (see Lines 760-765)

*2. A major concern is that the detection of infected cells is not nearly as rigorous at is needs to be. For instance, in 2b the virus UTR is detected everywhere? Is it tropic for T cells. Clear demonstration of how many viral reads/cell and the background ambient amounts of viral UTR detection are needed. This is critical to evaluate the degree of background noise from ambient RNA amplification, etc. and should be controlled for in analyses. Algorithms such as Cellbender would allow the authors to control for ambient RNA detection and more definitively identify infected cells. The viral reads detected across the different cell types after controlling for this background detection should be provided. This is critical to any interpretation of the paper.*

**Response:** Thank you for your valuable comment. Indeed, viral RNA contamination can occur during the single-cell sequencing library preparation, leading to potential false-positive results as viral RNAs may adhere to uninfected cells. Additionally, immune cells possess antigen uptake and presentation capabilities, enabling viral RNA detection even in the absence of productive infection due to phagocytosis of viral particles. We have re-analyzed the data using the SoupX algorithm to correct for ambient RNA contamination, and the virus gene expression profiles across cell clusters have been included in the supplementary materials (S4 Table 1-3) and figure 2b, 4b and 6c.

*3. Figure 2f is uninterpretable despite trying to find a description in text, figure itself, legend, and methods. Is this cells from infected chickens compare to the same types from uninfected chickens? A volcano plot would make more sense? The same problem is noted in Figure. 7d*

**Response:** Thank you for your insightful comment. We appreciate the opportunity to clarify any confusion regarding the figures. Regarding Figure 2f, it presents the differential gene expression analysis between infected and control groups for the same cell types. The size of the dots represents the log2 fold change in the proportion of cells expressing a given gene within a particular cell cluster in the infected group compared to the corresponding cell cluster in the control group. The color of the dots indicates the log2 fold change in the average expression level of that gene within the cell cluster in the infected group relative to the control group. As per your suggestion, we have included volcano plots in the supplementary materials for a more intuitive representation of the differential expression data (S1 Fig 4).

For Figure 7d, the heatmap colors depict the difference between the gene module expression signatures in a given cell cluster from the infected group and the corresponding cell cluster from the control group (infected group eigen - control group eigen). We have revised the figure legend and the methods section to provide a clearer explanation of the data representation. (see Lines 912-913, 1456-1460)

*4. The authors state that they validated the RNA data using immunohistochemistry by noting colocalization between the N protein and the collecting duct marker AQP2 or distal tubule marker CALB1. However, in Fig. 2, it is unclear if there really is overlap between the collecting duct specific AQP2 and the IBV N protein as it looks to me they are proximal, but non-overlapping in panel a. There also appears to be some non-specific staining with the N-protein in both panels, but it’s odd that the IBV panel alone in panel b shows no staining whatsoever yet there is visible red staining in the overlay panel suggesting that the same exposure/filtering may not have been performed. Thus, it is hard to trust the conclusion that this corroborates the scRNA-seq data. The lack of data showing the infected cells also appears to be a lost opportunity to use the single cell data to explore how infected cells signal and communicate in comparison with uninfected cells. Such an analysis would significantly strengthen the manuscript.*

**Response:** Thank you for your valuable comment. We appreciate the opportunity to clarify the points raised regarding the figures. If we understand correctly, are you referring to Figure 3?

1) Regarding Fig. 3, AQP2 is a specific marker for collecting duct cells, functioning to transport water molecules from the collecting duct lumen into the cells. AQP2 is localized specifically to the apical membrane of collecting duct cells, facing the lumen. On the other hand, the IBV N protein localizes to the cytoplasm during viral replication. Therefore, the lack of co-localization between AQP2 and the N protein is expected, as their subcellular localizations differ. However, in the merged image, it is evident that the IBV N protein resides within AQP2-expressing cells, supporting the hypothesis that IBV can infect collecting duct cells. We have revised the results description to clarify this observation. (see Lines 161-166, 1365-1368)

2) In Fig. 3b, we used green fluorescence to label the distal convoluted tubule marker CALB1 and red fluorescence for the IBV N protein. Conversely, in Fig. 3a, red fluorescence labeled the collecting duct marker AQP2, while green fluorescence labeled the IBV N protein. This difference in fluorescence labeling may have caused confusion (the variation in labeling patterns is due to the optimization required for multiplex immunofluorescence experiments). Upon re-examining Fig. 3b, we observed positive green staining for CALB1 in both infected and control groups, while positive red staining for the IBV N protein was only present in the infected group. This finding supports the hypothesis that IBV can infect distal convoluted tubule cells. We have updated the figure legend to provide clearer explanations. (see Lines 1365-1370)

3) As per your suggestion, after correcting for ambient viral RNA using the SoupX algorithm, we have performed differential gene expression analyses on IBV-infected cells, enhancing the manuscript. (see Lines 510-541,1476-1483)

We sincerely appreciate your feedback, as it has allowed us to improve the clarity and interpretability of our findings.

*5. Fig. 1F is highly unclear, as it is not stated what is being compared. Presumably this is infected relative to uninfected, but this is not explained. It would also be helpful to provide context in terms of how many genes are differentially expressed and what other genes might be different, as it’s unclear if these examples were cherry-picked.*

**Response:** Thank you for your valuable comment. If we understand correctly, are you referring to Figure 2f? Following your earlier suggestions, we have revised the figure caption to enhance clarity and facilitate interpretation (see Lines 1359-1363). Additionally, we have included volcano plots in the supplementary materials (S1 Fig 4). In the supplementary materials, we have also provided detailed information on the gene expression differences for all cell subpopulations between the infected and control groups (see S1 Table 1-3).

*6. In the bursa, the results of the scRNA-seq and IHC are diffuclt to reconcile. The authors detect viral RNA in macrophages, but cannot confirm this by RNA-seq. They also detect viral protein in FAE cells, but do not report whether these cells have RNA. The nomenclature between FAE cells and interfollicular epithelial cells is very confusing and needs to be clarified, with clear indication for which cells have both viral RNA and protein.*

**Response:** We sincerely appreciate your valuable feedback. In response to your comments, we have made the following clarifications and updates in our manuscript:

1) In the single-cell sequencing data from bursa, we detected viral RNA within macrophages, with detailed information provided in S4 Table 2. However, in our dual-immunofluorescence experiments, we did not observe viral protein localization in TIMD4^+^ macrophages.

2) In the single-cell sequencing data, follicle-associated epithelium (FAE) cells were not clustered into a individual cell cluster. This may be attributed to their gene expression patterns being more similar to reticular epithelial cells (ERCs), leading to their classification into the ERC cluster during the dimensionality reduction and clustering process. Notably, both FAE and ERCs originate from mesenchyme. During development, ERCs extrude from the epithelial basement membrane in the follicle, with the uppermost layer of ERCs differentiating into FAE^[1, 2]^. We also found a few cells expressing the FAE marker gene CSF1R in the ERC cluster. We have included this observation in the discussion section of our manuscript. (see Lines 606-615)

3) We have further clarified the terminology used for cell types and the specific conditions of viral-infected cells, incorporating these modifications into the main text of our manuscript. (see Lines 297-317)

*7. Several communication platforms also weight interactions by whether there is evidence for downstream signaling, and such an approach could be beneficial in finding ‘true’ receptor ligand interactions. This is important because just because a receptor and ligand are expressed does not necessarily mean that the interaction is active unless there is evidence of downstream signaling. It is unclear whether the signaling strength panels in* *Figs. 3e and 5e and 6g, it is unclear how the signaling strength of different pathways has been weighted. The figure legend needs more detail. Is this downstream signaling from these pathways or just the receptor/ligand? I could not find this description in the methods either.*

**Response:** We sincerely appreciate your valuable feedback and comment.

1) Cell communication analysis is commonly used to assess signaling interactions among different cell types in single-cell sequencing data. There are two main approaches to this analysis: The first is based solely on the expression patterns of ligand genes in signaling cells and receptor genes in receiving cells, known as the Ligand-receptor method. The cellchat algorithm is a prime example of this approach. The second, the Ligand-receptor target method, builds upon the first by also considering the expression of genes in the downstream signaling pathways of the receiving cells. Although the latter incorporates downstream target genes of ligands, its robustness is somewhat limited compared to the Ligand-receptor method due to the networked nature of intracellular signaling pathways, where different pathways can influence each other^[3]^. These methods are complementary, providing a more comprehensive picture of cell communication. Following your advice, we employed a novel approach called multinichenet^[4]^, which constructs a matrix of ligands and their activated target genes and weights the expression of ligands, receptors, and downstream target genes. This method allows for the assessment of whether downstream signaling pathways in target cells are activated, facilitating the construction of a cell-level cascade communication network. We have added the results of the multinichenet cell communication analysis as a complement to the cellchat analysis in the supplementary material, offering readers a more complete view of cellular communication (S2 Fig. 1-3 and Table 10-12). This methodological addition is also detailed in the methods section. (see Lines 236-266, 347-371, 402-418, 859-881)

2) In the CellChat algorithm^[5]^, when calculating the structural similarity between two signaling pathways, features considered include differences in the average path length distribution, network diameter, and centrality measures between the networks. Each feature is weighted equally in the calculation of structural similarity. In brief, CellChat operates under the assumption that there is insufficient a priori knowledge to prioritize or weigh the importance of different pathways, treating all pathways as equally important to avoid introducing bias. In the standard output of cellchat for cell communication, the communication probability (p) for all ligand/receptor pairs (i.e., signaling pathways) is first calculated, with p < 0.05 considered indicative of active signaling communication. This leads to the summation of the number of pathways showing signaling communication for each cell subpopulation (e.g., Cell Cluster A, as a signaling cell, engages in 5 signaling communications; as a receiving cell, it engages in 4). Based on this, the signaling output strength and signaling receiving strength for specific pathways in each cell cluster can be determined. For a particular signaling pathway, the sum of its signaling output strength across all cell clusters equals its total signaling activation strength in the sample. For a specific cell subpopulation, the sum of its signaling output strengths across all pathways constitutes its overall signaling output strength, and similarly for signaling receiving strength (For instance, if the signaling pathway 'a' has an output strength of 0.1 in Cell Cluster A and 0.2 in Cell Cluster B, then the total activation strength of pathway 'a' is 0.1 + 0.2 = 0.3. If Cell Cluster A has an output strength of 0.1 on pathway 'a' and 0.2 on pathway 'b', then the total activation strength of Cell Cluster A is 0.1 + 0.2 = 0.3). The CellChat algorithm allows for statistical testing of cell communication across different groups, identifying statistically significant differences in signaling pathways. By subtracting the signaling communication strengths between groups, we can delineate the differences in signaling strength for specific cell populations or pathways between groups (in our data, this is infected group - control group). In Figures 3e, 5e, and 6g, we calculated the differences in signaling receiving and output strengths across all cell subpopulations and pathways between groups (infected - control), represented in the heatmap by the upper left and lower right triangles, with color intensity indicating the strength of the difference. Additionally, the overall strength difference of different signaling pathways between infected and control groups is displayed as stacked bar charts to the right of the heatmap. All underlying data for signaling communication strength used in the plots are provided in Supplementary Material S2. The CellChat algorithm calculates cell communication based solely on the expression patterns of ligand and receptor genes in signaling and receiving cells, respectively, meaning the signaling communication strengths depicted are derived from the expression levels of these genes. We have included a more detailed description of this process in the text. (see Lines 830-858, 1370-1390, 1408-1420, 1433-1445)

*8. Similarly in Fig. 5b, the co-localization between TIMD4 and N-protein is not apparent.*

**Response:** Thank you for your valuable feedback. Actually, we found no colocalization between TIMD4 and the N protein of IBV; the IBV N protein was not detected in macrophages expressing TIMD4. Additionally, positive staining for the IBV N protein was absent in the macrophage-rich follicles of bursa. Although viral RNA was indeed detected in macrophages within single-cell sequencing data, this could be attributed to the phagocytosis of viral particles or infected cells rather than direct infection. While some literature indicates that certain IBV strains may potentially infect macrophages, the infectivity of the strain used in our study towards macrophages requires further investigation to confirm. We have elaborated on this point in both the results and discussion sections of our manuscript. (see Lines 307-317)

*9. The layout and description of Figs. 7 and 8 is difficult to interpret. The full parameters for the WGCNA analysis are not described but are critical to interpretability and reproducibility. The rigor with which the modules are defined is not clear. For instance, a Jaccard index heatmap depicting the overlap in gene membership between all discovered modules would be more interpretable as in Kazer et al. PMID XX. The the expression of these modules across cell types and tissues could be displayed. Unclear what is meant by “enrichment of immune-related gene modules”. Rather that describing the modules by color, it might be nice to understand what these different modules described in terms of biology.*

**Response:** Thank you for your valuable feedback. In response to your comments, we have made several revisions to improve the clarity and understandability of our manuscript, particularly concerning Figures 7 and 8, and the methods section.

1) We have revised the figure captions and methods section to facilitate a better understanding of Figures 7 and 8. Additionally, we have added a flow chart of our Weighted Gene Co-expression Network Analysis (WGCNA) process in the supplementary material (S3 Fig. 7). In brief, building upon the classical WGCNA approach, we incorporated differential gene expression data (infected group vs. control group) and transcription factor information into the co-expression network. (see Lines 1456-1475)

2) Within the methods section, we detailed the parameters used in our WGCNA analysis. The POWER value was set to 8. After identifying gene modules, we conducted t-tests on these modules to determine their correlation coefficients, merging modules with a correlation coefficient greater than 0.7. The merged modules were then subject to a further round of correlation analysis. In the supplementary materials (S3 Fig. 4), the relationships between various modules are illustrated through a Jaccard index heatmap, and the expression patterns of module eigengenes across different cell subpopulations are presented in a heatmap format in S3 Fig 5. All other parameters were set to the defaults of the R package WGCNA. Following the identification of gene modules and the genes within them, we conducted enrichment analyses to ascertain the biological functions of these gene modules (S3 Fig. 3). Among these modules, we identified seven whose biological functions are predominantly related to immune responses. We have designated these as immune-related gene modules and presented the results of their enrichment analyses in Figure 7c. (see Lines 760-765)

**Part III – Minor Issues: Editorial and Data Presentation Modifications**

**Reviewer #1：**

*1. Line 36-STAT-This should probably be* *STAT1. If so, it is associated with more than apoptosis.*

**Response:** Thank you for your valuable comment. STAT indicates STAT1 and STAT3. STAT gene plays an important role in the process of immune response, as a transcription factor regulating the expression of interferon, interleukin and other cytokines.

*2. Line 77, line 601-1 day old chicks were used in this study. Justification for use of this age animals should be provided. Also more description of the IBV strain used in this study should be included. Not all strains infect the kidney, and chicks of this age commonly succumb to a respiratory infection, rather than renal disease. The infectious model should be clarified.*

**Response:** Thank you for your insightful comment. The choice to sample at 5 days post-infection (dpi) from 1-day-old SPF chickens inoculated with IBV was strategic, based on the virus's replication pattern. Initially, IBV replicates in the trachea before infecting the kidneys and bursa. Sampling too early would miss the infection in the kidneys and bursa, while sampling too late might result in the clearance of the virus from the trachea. At 5 dpi, tissues from the trachea, kidneys, and bursa are concurrently infected, allowing for sampling and single-cell sequencing analysis from the same infected chicken, thus ensuring consistency in the sequencing data. The IBV strain used in our study has been previously demonstrated to possess the capability to infect the kidneys, a fact we have corroborated through immunofluorescence in our manuscript. The infection was administered via oculonasal eye dropper inoculation, a common method for IBV infection models^[6]^. We have provided a more detailed explanation of these aspects in the methods section of our manuscript. (see Lines 760-765)

*3. Line 180-“In conclusion our study demonstrates…”-This sentence should be modified since the data do not show a disruption of water-salt balance but rather are consistent with physiological studies showing this result.*

**Response:** Thank you for your valuable comment. We modified the relevant content to reconcile the description and analysis results. (see Lines 182-188)

*4. Line 185-Much of the manuscript discusses cell-cell communication. These types of analyses should be discussed in more detail for those readers not familiar with the concept or approach.*

**Response:** Thank you for your valuable comment. We have added details about the methodology of this analysis to the methods and results to make this part easier to understand. (see Lines 190-201)

*5. Line 240-“Considering that bursa…”-Is there independent support for this hypothesis? If so, a reference should be provided.*

**Response:** Thank you for your valuable feedback. The details regarding the development of bursa have indeed been extensively described in previous research^[1, 2]^. Within avian immunology, the cells covering the mucosal surface of bursa and partitioning the follicles are defined as interfollicular epithelial cells (IFE). Consequently, there is no distinct classification as rectal epithelial cells (REC) in this context, making the categorization of this cell cluster (REC) as IFE more accurate. We have made revisions to this section and the subsequent analysis to reflect this clarification. We apologize for any confusion caused and appreciate your guidance in this matter.

*6. Lines 259-267-Quantitative analyses, using Pearson’s coefficient should be provided to support these conclusions.*

**Response:** Thank you for your insightful feedback. Indeed, the N protein of IBV does not colocalize in the true sense with these proteins. Our intention in the manuscript was to indicate that the IBV N protein was detected within cells expressing specific markers, thereby identifying the cell types infected by IBV. For instance, the presence of the IBV N protein in cells expressing AQP2 demonstrates that IBV can infect collecting duct cells. However, it is important to clarify that the IBV N protein does not colocalize with AQP2; they merely co-occur within the same cell. We have updated the manuscript to more accurately reflect our findings and apologize for any confusion our previous wording may have caused. (see Lines 161-166, 307-317, 1404-1408)

**Reviewer #2:**

*Small point. Figure 3d refers to panel a but should refer to panel c. Also text says 1b when it refers to 2b*

**Response:** Thank you for your valuable comment. We have revised the relevant part.

**References:**

[1] Nagy N, Magyar A, Dávid C, Gumati MK, Oláh I. Development of the follicle-associated epithelium and the secretory dendritic cell in the bursa of fabricius of the guinea fowl (Numida meleagris) studied by novel monoclonal antibodies. Anat Rec. 2001;262(3):279-92. doi: 10.1002/1097-0185(20010301)262:3<279::Aid-ar1038>3.0.Co;2-i. PubMed PMID: 11241196.
[2] Yvernogeau L, Nagy N, Dunon D, Robin C, Jaffredo T. Chapter 3 - Development of the avian hematopoietic and immune systems. In: Kaspers B, Schat KA, Göbel TW, Vervelde L, editors. Avian Immunology (Third Edition). Boston: Academic Press; 2022. p. 45-69.
[3] Luo J, Deng M, Zhang X, Sun X. ESICCC as a systematic computational framework for evaluation, selection, and integration of cell-cell communication inference methods. Genome Res. 2023;33(10):1788-805. Epub 20231012. doi: 10.1101/gr.278001.123. PubMed PMID: 37827697; PubMed Central PMCID: PMCPMC10691505.
[4] Browaeys R, Gilis J, Sang-Aram C, Bleser PD, Hoste L, Tavernier S, et al. MultiNicheNet: a flexible framework for differential cell-cell communication analysis from multi-sample multi-condition single-cell transcriptomics data. bioRxiv. 2023:2023.06.13.544751. doi: 10.1101/2023.06.13.544751.
[5] Jin S, Guerrero-Juarez CF, Zhang L, Chang I, Ramos R, Kuan CH, et al. Inference and analysis of cell-cell communication using CellChat. Nat Commun. 2021;12(1):1088. Epub 20210217. doi: 10.1038/s41467-021-21246-9. PubMed PMID: 33597522; PubMed Central PMCID: PMCPMC7889871.
[6] You R, Liu K, Huang M, Tang L, Zhang X, Huang Y, et al. Identification and Comparison of the Sialic Acid-Binding Domain Characteristics of Avian Coronavirus Infectious Bronchitis Virus Spike Protein. J Virol. 2023;97(5):e0048923. Epub 20230425. doi: 10.1128/jvi.00489-23. PubMed PMID: 37097156; PubMed Central PMCID: PMCPMC10231253.
